# Single-atom cobalt array bound to distorted 1T MoS_2_ with ensemble effect for hydrogen evolution catalysis

**DOI:** 10.1038/s41467-019-12997-7

**Published:** 2019-11-19

**Authors:** Kun Qi, Xiaoqiang Cui, Lin Gu, Shansheng Yu, Xiaofeng Fan, Mingchuan Luo, Shan Xu, Ningbo Li, Lirong Zheng, Qinghua Zhang, Jingyuan Ma, Yue Gong, Fan Lv, Kai Wang, Haihua Huang, Wei Zhang, Shaojun Guo, Weitao Zheng, Ping Liu

**Affiliations:** 1 State Key Laboratory of Automotive Simulation and Control, Department of Materials Science, Key Laboratory of Automobile Materials of MOE, Jilin University, Changchun, 130012 China; 2 Key Laboratory for Renewable Energy, Beijing Key Laboratory for New Energy Materials and Devices, Laboratory of Advanced Materials and Electron Microscopy, Beijing National Laboratory for Condensed Matter Physics, Institute of Physics, Chinese Academy of Sciences, Beijing, 100190 China; 3 Department of Materials Science and Engineering, Peking University, Beijing, 100871 China; 4 4BIC-ESAT, College of Engineering, Peking University, Beijing, 100871 China; 5 Beijing Synchrotron Radiation Facility, Institute of High Energy Physics, Chinese Academy of Sciences, Beijing, 100049 China; 6 Shanghai Synchrotron Radiation Facility, Shanghai Institute of Applied Physics, Chinese Academy of Sciences, Shanghai, 200120 China; 7 Department of Chemistry, Brookhaven National Laboratory, Upton, NY 11973 USA

**Keywords:** Catalytic mechanisms, Electrocatalysis, Two-dimensional materials

## Abstract

The grand challenge in the development of atomically dispersed metallic catalysts is their low metal-atom loading density, uncontrollable localization and ambiguous interactions with supports, posing difficulty in maximizing their catalytic performance. Here, we achieve an interface catalyst consisting of atomic cobalt array covalently bound to distorted 1T MoS_2_ nanosheets (SA Co-D 1T MoS_2_). The phase of MoS_2_ transforming from 2H to D-1T, induced by strain from lattice mismatch and formation of Co-S covalent bond between Co and MoS_2_ during the assembly, is found to be essential to form the highly active single-atom array catalyst. SA Co-D 1T MoS_2_ achieves Pt-like activity toward HER and high long-term stability. Active-site blocking experiment together with density functional theory (DFT) calculations reveal that the superior catalytic behaviour is associated with an ensemble effect via the synergy of Co adatom and S of the D-1T MoS_2_ support by tuning hydrogen binding mode at the interface.

## Abstract (web summary)

While single-atom catalysis offers an efficient materials usage, the ambiguous interactions with supports poses a difficulty in understanding catalytic performances. Here, authors show an ensemble effect via synergy of Co adatoms and the S of MoS_2_ supports to boost hydrogen evolution activities.

## Introduction

Hydrogen (H_2_), as a zero-emission, renewable energy source, has attracted increasingly extensive attention owing to its important role in solving the environmental issues^1,2^. Water splitting catalysis is one of the most efficient approaches for H_2_ generation because of its high-efficiency energy conversion^3^. Platinum (Pt) is the best catalyst for the hydrogen evolution reaction (HER); however, the high cost and limited earth abundance of Pt expose tremendous limitations for large-scale implementation^4^. Therefore, the critical determinant for energy storage in electrolytic systems is the development of robust and efficient alternative catalysts that are cheap and earth-abundant. Remarkable advances have been made in developing efficient non-noble materials as Pt substitutes for HER^5^. Unfortunately, a large gap in their HER catalytic performance still exists for completely substituting Pt owing to their lack of more efficient active sites and difficulty in maximizing the intrinsic activity of their active sites^6,7^. Atomically dispersed catalysts with single metal atoms or mononuclear metal complexes anchored on supports represent the lowest size limit to achieve maximum atom efficiency, providing the most ideal platform for catalysis^8–10^. However, the biggest issue is that previously reported atomically dispersed catalysts have been primarily in the form of atomic clusters, especially at high loading amounts, owing to the ambiguous interactions between the metal atoms and supports, posing a difficulty in maximizing the catalytic efficiency^11–15^.

Herein, we report the procedure for making single-atom cobalt (Co) array covalently bound onto distorted 1T MoS_2_ nanosheets (denoted as SA Co-D 1T MoS_2_) via Co-S bonds through electrochemical cyclic voltammetry (CV) leaching of Co nanodisks (NDs)-MoS_2_ nanosheet hybrids. The strain induced by lattice mismatch and the formation of Co-S covalent bond between Co and MoS_2_ in the Co ND-MoS_2_ nanosheet hybrids are shown to be critical for achieving the phase transformation of MoS_2_ from the semiconductive 2H to metallic distorted 1T (D-1T) phase^16^. The SA Co-D 1T MoS_2_ catalyst exhibits the Pt-like electrocatalytic activity for HER in acid electrolyte with a very low overpotential of 42 mV at 10 mA cm^−2^, low Tafel slope of 32 mV dec^−1^ and excellent long-term and cycling stability. The HER activity of SA Co-D 1T MoS_2_ is very close to that of commercial Pt^17^. The ultrahigh activity is proved to be the ensemble effect through the synergy of single Co atom and S in distorted 1T phase of MoS_2_ that possesses the optimal hydrogen-binding energy.

## Results

### Synthesis and characterization of SA Co-D 1T MoS_2_

**Fig. 1:**
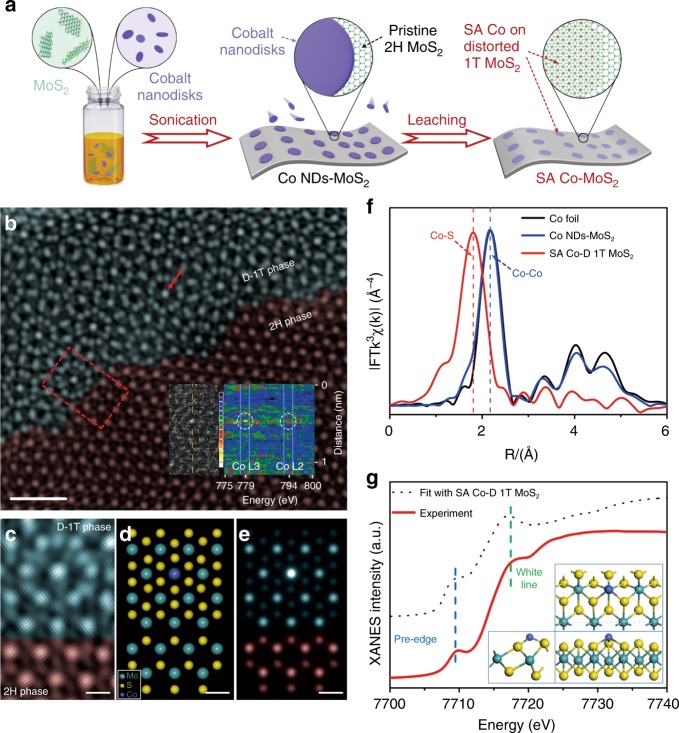
Schematic illustration of synthetic method for SA Co-D 1T MoS_2_ and its characterization. **a** Schematic diagram of the fabrication process for SA Co-D 1T MoS_2_. **b** Aberration-corrected HAADF-STEM image of SA Co-D 1T MoS_2_, showing the obvious junction between SA Co-D 1T MoS_2_ (dark cyan) and pristine 2H MoS_2_ (wine). The inset shows the HRTEM and EELS spectrum of SA Co-D 1T MoS_2_ (scale bar: 1 nm). **c** Enlarged HAADF-STEM image in the red square area of **b** (scale bar: 2 Å). **d** Theoretical model and **e** simulated STEM images using QSTEM simulation software (scale bar: 2 Å). **f** FT-EXAFS spectra of SA Co-D 1T MoS_2_ and bulk cobalt foil at the Co K-edge. **g** Co K-edge XANES of SA Co-D 1T MoS_2_ and fitted curve. The inset shows the atomic structure of SA Co-D 1T MoS_2_

Figure 1a shows the schematic illustration of the preparation of the SA Co-D 1T MoS_2_ catalysts using an assembly/leaching process, which ensures the successful introduction of Co atoms on the basal plane other than on the defect or edged sites of MoS_2_. Ordered Co NDs with an average diameter of 12 nm and height of 4.5 nm (Supplementary Fig. 1) were first assembled onto the surface of MoS_2_ nanosheets by sonication-induced Co-S bonding, in which the assembling amount is highly dependent on the sonication power (Supplementary Fig. 2). Phonons at ultrasonic power may help to closely contact Co nanodisks with MoS_2_ nanosheets, and provide the energy for the Co-S bond formation through ultrasonic cavitation (Supplementary Figs. 3 and 4, Supplementary Table 1)^18,19^. The Co ND-modified MoS_2_ heterostructures (Co NDs/MoS_2_) were further treated by electrochemical CV cycles (Supplementary Fig. 5)^20^. This electrochemical CV leaching resulted in the disappearance of Co–Co bond and remaining of Co-S bond in the resultant SA Co-D 1T MoS_2_ (Supplementary Figs 6 and 7 and Supplementary Table 2). Single-atom Co array was formed and in situ anchored onto the MoS_2_ surface, where the Co NDs were assembled. Transmission electron microscopy (TEM) image, selected area electron diffraction pattern (Supplementary Fig. 8) and X-ray diffraction (XRD) pattern (Supplementary Fig. 9) show that the Co nanodisks is well contacted with the MoS_2_ by forming heterostructures, and there is no Co nanocrystal after the electrochemical leaching. Energy-dispersive X-ray spectroscopy mapping analysis reveals that Co is evenly dispersed in SA Co-D 1T MoS_2_ (Supplementary Fig. 8f–h). High-resolution TEM (HRTEM) image shows that MoS_2_ is intensely disordered after bonding with Co atoms (Supplementary Fig. 8i). High-angle annular dark-field scanning transmission electron microscopy (HAADF-STEM) was further used to intuitively observe the atomic dispersion of Co and the phase transition of MoS_2_ (Fig. 1b–e). The disordered structure of SA Co-D 1T MoS_2_ is totally different from the Moiré patterns when two or few layers of MoS_2_ aggregate with random orientation (Supplementary Fig. 10). The atomically isolated Co species (bright spots marked by the red arrow) are dispersed on the D-1T MoS_2_ matrix, and the obvious interface between SA Co-D 1T MoS_2_ and the 2H MoS_2_ is confirmed by both the HAADF-STEM image (Fig. 1c) and simulated pattern (Fig. 1d, e). Atomically electron energy loss spectroscopy (EELS) line scanning was also conducted (inset of Fig. 1b), showing two Co peaks at 779 eV and 794 eV, which correspond to Co L3 and Co L2, respectively^21^. The statistics for the size of bright dots (Co atom) were determined to be in the range of ~ 2–3 Å, close to that of a single Co atom (Supplementary Fig. 11)^22^. These results indicate that single Co atoms are uniformly bound to the top of the Mo atoms on the MoS_2_ slab (Supplementary Fig. 12)^23^. The Co mass loading amount on SA Co-D 1T MoS_2_ is determined to be 3.54 wt.% by inductively coupled plasma mass spectrometry technique, being in agreement with those determined by EDX technique (3.60 wt.%, Supplementary Fig. 13) and XAFS spectrum simulation (4.07 wt.%, Supplementary Fig. 14). In addition, using the same assembly/leaching strategy, we also successfully prepared SA Ni D-1T MoS_2_ and SA Fe D-1T MoS_2_ catalysts (Supplementary Figs 15–25, Supplementary Table 3 and 4) on the premise that the Ni and Fe nanoplates were used to ensure their large area sufficient contact with MoS_2_ instead of their nanoparticles counterparts, further indicating that the method is universal and can be extended to any 2D metals or metal oxides, which meets the criterions.

### Coordination environment confirmation of SA Co-D 1T MoS_2_

To verify the coordination environment of atomic Co dispersed on SA Co-D 1T MoS_2_, the extended X-ray absorption fine structure (EXAFS) and X-ray absorption near-edge structure (XANES) spectroscopies were performed (Fig. 1f–g)^24^. A single strong shell at 1.79 Å in R-space of the EXAFS spectrum indicates the exclusive existence of Co-S bond for SA Co-D 1T MoS_2_ (Fig. 1f)^25,26^. The charge density difference from the first-principle calculation also confirms the existence of the Co-S covalent bond (Supplementary Fig. 26)^27^. We also characterized the cross-section structure and interactions for the side view of the heterostructures. Supplementary Fig. 27 shows the TEM and HRTEM images of the Co NDs-MoS_2_. The crystal alignment between Co nanodisks and MoS_2_ nanosheets is that the contact layer of Co was covalently bonded with the S atom on the surface of MoS_2_, and also the contact layer of MoS_2_ nearest to Co nanodisks shows a phase transformation from 2H to distorted 1T phase. We can also observe from the cross-section views of Co nanodisks-MoS_2_ that there is a well-defined covalently bounded interface between the counterparts. Using the SA Co-D 1T MoS_2_ (Co atom was coordinated with three adjacent sulfur atoms, and located at the site directly above the center Mo atom) as a model, we also simulated it’s XANES spectrum (dotted lines in Fig. 1g)^28^. The fitting results for SA Co-D 1T MoS_2_ with a Co concentration of 3.70% show the same two main energy features at 7710 eV (pre-edge) and 7718 eV (white line) as those of the experimental results, further proving that the Co atom is right on the top of Mo atom instead of the replacement of Mo atom. All these extensive experimental and simulation results reveal that, in SA Co-D 1T MoS_2_, a single Co atom is coordinated to three adjacent sulfur atoms and located at the site directly above the Mo atom.

### Phase transition observation during the synthesis of SA Co-D 1T MoS_2_

**Fig. 2:**
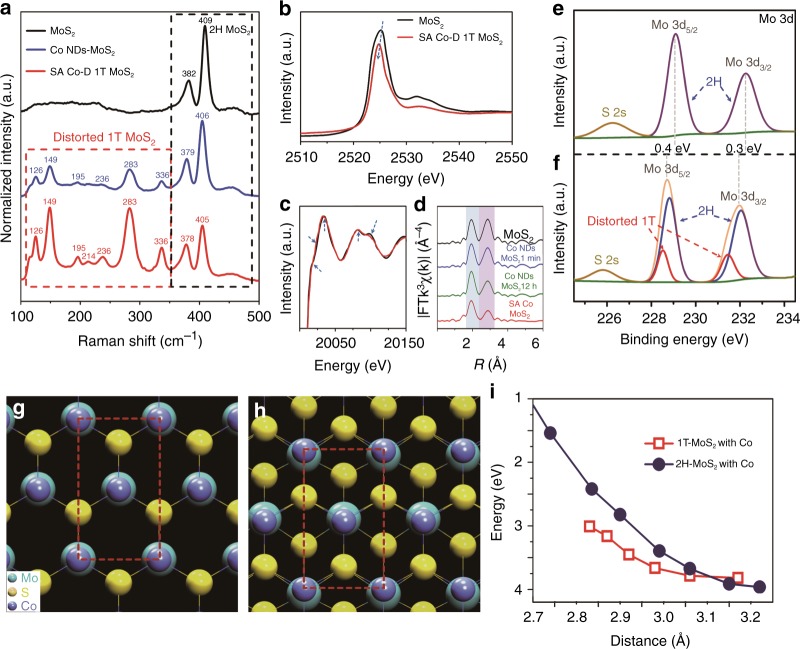
Characterization techniques and DFT calculation for the phase transformation of MoS_2_. **a** Raman spectra of pristine MoS_2_, Co NDs/MoS_2_, and SA Co-D 1T MoS_2_. **b**, **c** Mo L_3_-edge and K-edge XANES spectra of SA Co-D 1T MoS_2_ and pristine MoS_2_. **d** Mo K-edge EXAFS spectra variation during the preparation of SA Co-D 1T MoS_2_. **e**, **f** Mo 3d XPS spectra show the surface-binding state variation before **e** and **f** after the MoS_2_ phase transformation. **g** 2H and **h** 1T atomic structures of MoS_2_ assembled with Co atomic layer calculated by first-principles. **i** Energies of 2H MoS_2_ and 1T MoS_2_ assembled with Co atomic layer as a function of Co–Co distance calculated by first-principles method based on the single layer 2H MoS_2_ and atomic Co as the reference state with the formula ΔE = E_*2H-MS*_ + E_*Co*_—E_*MS-Co*_

Raman spectra of Co ND/MoS_2_ and SA Co-D 1T MoS_2_ show peak shifts of E^1^_2g_ and A_1g_, and new peaks between 100 and 350 cm^−1^ (i.e., 126, 149, 195, 214, 236, 283, and 336 cm^−1^) compared with those of pristine MoS_2_ (Fig. 2a), resulting from the phase transformation of the MoS_2_^29^. The results from time-dependent evolution of Raman spectra reveal that the phase transformation happened during the assembly process of Co NDs on MoS_2_ (Supplementary Fig. 28). The Mo L_3_-edge XANES spectrum (Fig. 2b) of SA Co-D 1T MoS_2_ shows a decreased peak intensity and blue shift to ~ 2525.12 eV, indicating the phase transformation from 2H to D-1T MoS_2_^30^. The Mo K-edge XANES spectrum (Fig. 2c) of SA Co-D 1T MoS_2_ also indicates a considerably widened energy-state distribution related to the Mo 4d electrons with the appearance of some unoccupied Mo 4d states after bonding with the SA Co (as labeled by the arrows)^31^. Fig. 2d shows that the second shell intensity of Mo K-edge EXAFS spectrum decreases after the Co NDs assembly. When SA Co-D 1T MoS_2_ was produced after CV leaching, the second shell intensity decreases more, indicating the further phase transformation from pristine 2H to D 1T of MoS_2_^32^.

The two polymorphs of MoS_2_ (pristine 2H phase and distorted 1T phase) can be identified by the XPS spectra of Mo 3d and S 2p. The binding energies of Mo 3d for the pristine 2H MoS_2_ (Fig. 2e) are obviously different from those of SA Co-D 1T MoS_2_ (Fig. 2f). The peaks at ~ 229.1 and 232.3 eV, corresponding to the binding energies of Mo^4+^ 3d_5/2_ and 3d_3/2_, shift to lower energy by 0.4 and 0.3 eV, respectively, and also a new doublet of Mo 3d spectrum located at 228.5 and 231.5 eV appears, which indicate the production of distorted 1T MoS_2_. The S 2p spectrum also shows a new doublet at 161.6 and 162.7 eV (Supplementary Fig. 29). These new doublets in Mo 3d and S 2p spectra can be assigned to the characteristic peaks of distorted 1T phase MoS_2_, further confirming the phase transformation from the pristine 2H to D 1T^33^. The evolution of Co 2 P spectrum during the assembly and CV leaching process also confirms the formation of the Co-S bond (Supplementary Fig. 30).

### Phase transition mechanism of MoS_2_ during the synthesis of SA Co-D 1T MoS_2_

Density functional theory (DFT) calculations were conducted to reveal the mechanism of the phase transition of MoS_2_ after assembling Co NDs. On the basal plane of MoS_2_ surface, it is found that there is strong interaction between the Co adatom and S atom. As shown in Fig. 2g, the Co atom prefers to occupy the top site of 2H MoS_2_. The adsorption energy is 3.96 eV/Co by the formula, ΔE_*ad*_ = E_*MS*_ + E_*Co*_ − E_*MS-Co*_, where E_*MS*_, E_*Co*_, and E_*MS-Co*_, are the energies of the isolated MoS_2_, isolated Co atom and MoS_2_ with adsorbed Co atom, respectively. Interestingly, the adsorption energy (4.37 eV/Co) of Co on 1T MoS_2_ (Fig. 2h) is larger than that on 2H MoS_2_. This means that Co atoms prefer to bond on 1T MoS_2_ than 2H MoS_2_. With the adsorption of Co atoms, the energy difference between 2H and 1T (ΔE = E_MS(1T)_ − E_MS(2H)_) is 184 meV/atom. That is, the phase transformation of MoS_2_ from 2H to 1T is still not thermodynamically favorable, but should be driven by the external force. According to the previous theoretical work, the strain can be used to modulate the phase transition from 2H phase to 1T^34^. In Fig. 2i, the stability of 2H and 1T phases varies with the coverage of Co or the Co–Co distance in the nearest neighbor. Only under the Co–Co distance lesser than 3.10 Å with a strain of ~3.70%, the 1T phase becomes to be more stable (Fig. 2i). It is noticed that the Co–Co distance on (111) facet of Co is about 2.51 Å and the lattice constant of MoS_2_ is ~3.16 Å. The mismatch between the lattice of metallic Co and pristine 2H MoS_2_ is large enough for the strain generation (Supplementary Fig. 31). In this case, the anchoring of Co atoms induces the coordination reconstruction of the MoS_2_ support to D-1T MoS_2_ owing to a charge density wave (CDW) state^35^. Such CDW state of D-1T MoS_2_ can trap the system into the deeper energy minimum on the potential energy surface. The CDW structures with different Co coverages are illustrated in Supplementary Fig. 32. These DFT results strongly support the experimental observation that phase transition happens after the assembly of Co NDs on MoS_2_ surface, during which the Co-S bond was formed with the assistance of sonication.

### HER activity of SA Co-D 1T MoS_2_

**Fig. 3:**
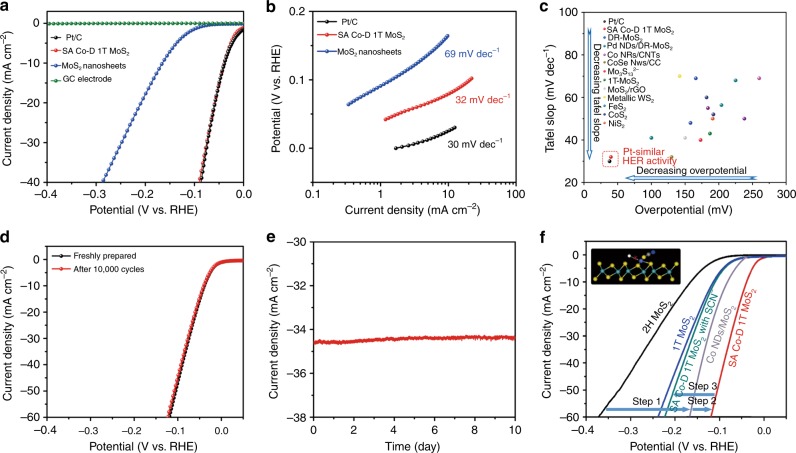
HER performance of SA Co-D 1T MoS_2_. **a** Polarization curves of different catalysts tested in Ar-saturated 0.5 m H_2_SO_4_. **b** Tafel plots for the catalysts derived from **a**. **c** HER activity comparison using the Tafel slope (mV dec^−1^) vs. overpotential at a current density of 10 mA cm^−2^. **d** Polarization curves of the SA Co-D 1T MoS_2_ after 10,000 CV cycles. **e** Time dependence of the current density for SA Co-D 1T MoS_2_ at a static overpotential of 100 mV vs. RHE. **f** HER polarization plots of MoS_2_, 1T MoS_2_ prepared by lithiation, Co NDs/MoS_2_ and SA Co-D 1T MoS_2_ without and with SCN^−^ ions. The inset shows that the cobalt HER active centers are blocked by SCN^−^ ions

The HER activities of the catalysts were studied in 0.5 m H_2_SO_4_ solution. SA Co-D 1T MoS_2_ with 3.54% Co loading amount exhibits an extremely small onset overpotential (*η*) of 42 mV vs. the reversible hydrogen electrode (RHE) for HER, much smaller than those of other non-precious metal catalysts and even comparable to that of 10% Pt/C (Fig. 3a, Supplementary Fig. 33, Supplementary Table 5 and 6). To gain a quantitative insight into the HER activity, the turnover frequency (TOF), which can reveal the intrinsic catalytic activity of a single active site of the catalyst, was calculated (Supplementary Fig. 34). SA Co-D 1T MoS_2_ shows a TOF of 7.82 s^−1^ at an overpotential of 100 mV vs. RHE. Also, after the SA Co atom bonding, the catalyst shows a lower EIS response than the MoS_2_ (Supplementary Fig. 35), indicating a fast kinetics for hydrogen evolution^36^. The Tafel slope of SA Co-D 1T MoS_2_ is 32 mV dec^−1^ (Fig. 3b), similar to that of Pt/C (30 mV dec^−1^), and surpassing those of previously reported non-Pt-based materials (Fig. 3c) that using a graphite rod as the counter electrode instead of Pt foil to avoid Pt contamination^37^. The low Tafel slope indicates that SA Co-D 1T MoS_2_ processes a Tafel rate-determining-step mechanism for HER, instead of the common Volmer reaction^38^. In addition, a series of control experiments and DFT calculations prove that other MoS_2_/Co catalysts prepared by using Co clusters, nanoparticles, or ions on MoS_2_ cannot achieve such high HER performance (Supplementary Figs 36–45, Supplementary Table 7). The SA Ni D-1T MoS_2_ and SA Fe D-1T MoS_2_ catalysts also show enhanced HER activity relative to pristine MoS_2_, but lower than SA Co-D-1T MoS_2_ (Supplementary Fig. 46 and Supplementary Table 8).

### HER stability of SA Co-D 1T MoS_2_

SA Co-D 1T MoS_2_ shows excellent cycling stability and long-term durability for HER in acidic media. The linear sweep voltammetry (LSV) curves measured for SA Co-D 1T MoS_2_ before and after 10,000 CV cycles between 0.1 and − 0.3 V *vs*. RHE exhibit a negligible current density loss (Fig. 3d). Figure 3e shows the long-term durability measured by performing continuous HER at a static overpotential of 100 mV. Consistent H_2_ generation was observed during the HER process (inset of Fig. 3e), and the current density remained without any decrease as the reaction proceeded for 10 days. The high stability of SA Co-D 1T MoS_2_ catalyst was also confirmed by HAADF-STEM (atomic structure stability), XPS (valence state and phase stability), XANES, and FT-EXAFS (coordination environment stability) after accelerating stability measurements, long-term air storage and heating treatment (Supplementary Figs 47–52 and Supplementary Table 9).

### The identification of reaction active site

The nature of the high activity of SA Co-D-1T MoS_2_ was disclosed by comparing the HER polarization plots of different catalysts (Fig. 3f). The HER activity of MoS_2_ was first improved by the assembly of Co NDs (Step 1, Co NDs/MoS_2_) that induced the phase transformation from 2H to D-1T (onset overpotential decreases from 162 mV to 85 mV at 10 mA cm^−2^) because the metallic 1T MoS_2_ can give higher conductivity and more active sites for better HER^39^. The HER performance of MoS_2_ was further improved to a Pt-like activity (onset overpotential decreases from 85 mV to 42 mV, similar to the Pt/C, by CV leaching to form single-atom Co sites on D-1T MoS_2_ (Step 2, SA Co-D-1T MoS_2_). Thiocyanate ion (SCN^−^, poisoning agent) was then used to block the Co active sites^40^. The addition of 10 mm SCN^−^ resulted in an increase of onset overpotential from 42 mV to 112 mV (Step 3, SA Co-D 1T MoS_2_ with SCN^−^), close to that of the 1T MoS_2_ prepared by lithiation. Therefore, the high HER activity of the SA Co-D 1T MoS_2_ catalyst is a synergetic effect of both the single Co atom and the D-1T MoS_2_, that is to say, both single Co atom site and 2H to D-1T phase transformation of MoS_2_ are contributory. Nevertheless, single-atom Co sites give the dominant contribution to Pt-like HER activity.

### Ensemble effect enhancing HER activity

**Fig. 4:**
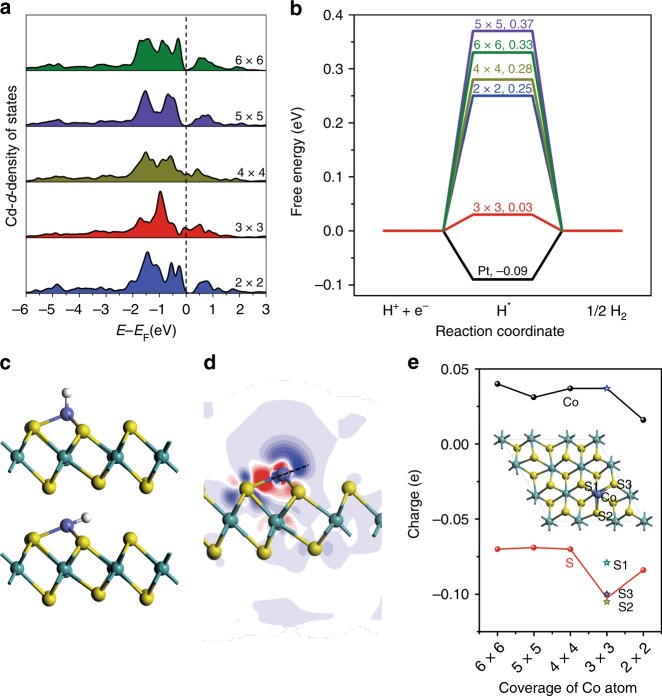
Theoretical calculation of SA Co-D 1T MoS_2_ for HER. **a** Calculated Co projected d-density of states for different coverage. **b** Calculated free-energy diagram for HER at a potential of U = 0 relative to the standard hydrogen electrode at pH = 0 for different atomic Co loading amounts. **c** Hydrogen adsorption modes on the single-atom Co-MoS_2_ 3 × 3 case. **d** The electron density difference of 3 × 3 case and **e** the electron charge of Co and S adjacent to Co as a function of Co coverage

DFT calculations were further performed to explore the origin of the high electrocatalytic activity of SA Co-D 1T MoS_2_ for the HER in acidic solution. According to the DFT calculations, the PDOS of Co 3d near the Fermi level for 3 × 3 and 4 × 4 cases are remarkably different from the others (Fig. 4a). It indicates that the Co adatoms are transformed from cationic to metallic with the coverage increasing to 2.08 and 3.70% (corresponding to 4 × 4 and 3 × 3 models) because of their transition CDW structures. In particular, the resulted increase in the empty state of Co close to the Fermi level for the 3 × 3 case is the most significant among the systems studies, being able to promote the hybridization between Co adatom and the hydrogen atom, and thus enhance the corresponding hydrogen binding. In addition, the d-peak at the Fermi level for the 3 × 3 case is stronger than that for 4 × 4 case, indicating the stronger hydrogen adsorption on cobalt. Such variation in Co 3d with the coverage strongly depends on the Co-MoS_2_ interaction, which corresponds to the so called electronic or ligand effect. The |ΔG_H*_| value is dependent on the Co coverage on D-1T MoS_2_ (Fig. 4b). SA Co-D 1T MoS_2_ with a Co coverage of 3.70% (3 × 3 superstructure) shows the lowest |ΔG_H*_| value of 0.03 eV, enabling the HER energy profile even closer to the ideal than Pt (111) with the |ΔG^Pt^_H*_| = 0.09 eV. Thus, a superior HER activity of SA Co-D 1T MoS_2_ catalyst over Pt is expected from a thermodynamics viewpoint (Supplementary Figs 53–55).

Most importantly, the prominent HER activity of SA Co-D 1T MoS_2_ in a 3 × 3 superstructure is attributed to the ensemble effect of D-1T MoS_2_ support and Co single-atom. Our calculation results show that the hydrogen adsorption at the upright position of Co on MoS_2_ (111) is not as favorable as that at the tilted top site toward the adjacent S atom on the surface (Fig. 4c) by 0.68 eV/unit. The preference for the tilted top site is associated with the ensemble effect at the Co-MoS_2_ (111) interface. The energy gain by moving from the upright to the tilted position is associated with the electrostatic attraction between the positively charged proton and negatively charged S adjacent to Co. Figure 4e plots the variation in Hirshfeld charge of Co and adjacent S as a function of Co coverage^41^. The positive Co atom is beneficial to the protonation process. Among the S ions, the one in a 3 × 3 superstructure displays the most negative charge, and therefore contributes the most to stabilize adsorbed H via the electrostatic interaction. Our DFT calculations highlight the importance of ensemble effect via the synergy of Co adatom and S of the MoS_2_ (111) support in tuning hydrogen-binding mode at the interface and thus achieving superior HER activity.

## Discussion

In summary, we report a unique designed single-atom array catalyst, SA Co-D 1T MoS_2_, with Pt-like HER performance by the top–down assembly/leaching strategy. During the assembly process, the strain induced by lattice mismatch and the formation of the covalent Co-S bond between Co ND and 2H MoS_2_ nanosheet are identified as the main reasons for 2H to D 1T phase transformation. The 2D morphology of Co NDs is the key in the preparation of SA Co-D IT MoS_2_ because that the large area contact of Co with the basal plane of MoS_2_ is essential to generate enough compressive strain for phase transformation. The active site blocking experiment reveals that the single-atom Co in SA Co-D 1T MoS_2_ is the principal catalytic center, although both single Co atom and 2H to D-1T phase transformation of MoS_2_ are contributory for HER catalysis. DFT calculations confirm that the high HER activity of this single-atom catalyst is mainly owing to an ensemble effect via the synergy of Co adatom and S of the MoS_2_ (111) support by tuning hydrogen-binding mode at the interface. The discovery of this SA Co-D 1T MoS_2_ catalyst highlights the area of tuning the structure and functionality of metal-TMD catalysts at the atomic scale, which holds promise for applications in large-scale water splitting electrolyzers.

## Methods

### Synthesis of MoS_2_ nanosheets

MoS_2_ was synthesized using standard solvothermal procedures^42^. In a typical synthesis, 24.7 mg of ammonium molybdate tetrahydrate ((NH_4_)_6_Mo_7_O_24_·4H_2_O, ACS Grade, Sigma Aldrich) and 53.2 mg of thiourea (CH_4_N_2_S, ACS Grade, Sigma Aldrich) were dissolved in 20 mL of Millipore water (18.2 MΩ cm) under vigorous stirring to form a homogeneous solution. The solution was then transferred into a 30 mL Teflon-lined stainless-steel autoclave and maintained at 220 °C for 18 h before the reaction system was allowed to cool down to room temperature. The final product was washed several times with water and ethanol to remove any possible ions and finally suspended in ethanol.

### Synthesis of Co nanodisks

Co nanodisks were synthesized using standard air-free procedures^18^. In a typical synthesis, 0.1 g of trioctylphosphine oxide (TOPO, Technical Grade, Sigma Aldrich) was degassed with argon (High purity) for 20 min in a three-neck flask, followed by the introduction of 15 mL of anhydrous o-dichloride benzene (DCB, Technical Grade, Sigma Aldric) and 0.1 mL of oleylamine (OA, Technical Grade, Sigma Aldric) under argon, heating to reflux (182 °C) and then rapidly injecting 0.54 g of cobalt carbonyl (Co_2_(CO)_8_, Co content ≥90%, Sigma Aldrich) containing 1–5% hexane diluted in 3 mL of DCB (precursor solution). After 10 min, the reaction was stopped by quenching in an ice water bath. The final products were processed by extracting the solution, washing several times with water and methanol to remove the possible ions and organic component, and finally suspending in argon-saturated water for storage.

### Synthesis of Ni nanodisks

Ni nanodisks were synthesized using an standard air-free procedure^43^. In a typical synthesis, 0.257 g of nickel(II) acetylacetonate (Ni(acac)_2_, Technical Grade, Sigma Aldrich) and 0.109 g of tungsten hexacarbonyl (W(CO)_6_, 97%, Sigma Aldrich) were added to 7 mL of oleylamine in a two neck flask. Then the mixture was pre-heated to 60 °C under an argon atmosphere with strong magnetic stirring for the air degassing. After 30 min, the solution mixture was slowly heated up to 160 °C, and then immediately cooled down to room temperature in a water bath. Subsequently, the dark solution obtained was washed with a mixture of n-hexane and ethanol twice. The obtained product was separated by centrifugation, further washed twice and dried under ambient conditions to acquire dark powders. Finally, the Ni nanodisks powder was stored in an argon-saturated glass bottle for further use.

### Synthesis of Fe_2_O_3_ nanodisks

Fe_2_O_3_ nanodisks were synthesized using standard hydrothermal procedures^44^. In a typical synthetic procedure, 0.8 g of Iron(III) nitrate nonahydrate (Fe(NO_3_)_3_·9H_2_O, ACS Grade, Sigma Aldrich) and 1.0 g of urea (NH_2_CONH_2_, Sigma Aldrich) were dissolved in 70 ml of Millipore water. Then, 8.28 g of Tween 80 (C_24_H_44_O_6_, AR, Adamas-beta Chemical Co.) and 1.3 g of P123 (Poly(ethylene glycol)-block-poly(propylene glycol)-block-poly(ethylene glycol), Mav ~ 5800, Sigma Aldrich) were added under magnetic stirring. When Tween 80 and Pluronic amphiphilic triblock copolymer were dissolved completely, the obtained red solution was carefully transferred into a 100 mL Teflon-lined stainless-steel autoclave, then sealed and maintained at 180 °C for 24 h. After cooled to room temperature, the dark-red colloidal suspension was obtained. The precipitate was collected by centrifugation at 8000 rpm for 10 min, and then resuspended in the isometric mixture of distilled water and ethanol. The washing centrifugation process was repeated for at least three times. Finally, the Fe_2_O_3_ nanodisks powder was stored in an argon-saturated glass bottle for further use.

### Assembly of Co/Ni/Fe_2_O_3_ nanodisks on MoS_2_

For the assembly of Co/Ni/Fe_2_O_3_ nanodisks onto MoS_2_. The Co/Ni/Fe_2_O_3_ nanodisks and MoS_2_ solution with an appropriate mass ratio were mixed together in a 20 mL bottle. The reaction system was degassed with argon for at least 20 min. Then, it was ultrasonically treated for 24 h in a thermostat reaction system with a constant temperature of 4 °C, power of 800 W and frequency of 40 KHz. The final product was collected by extraction from the solution, washed with water several times to remove the possible ions and organic component, and finally suspended in argon-saturated water for storage.

### Synthesis of SA Co/Ni/Fe D 1T MoS_2_

The Co/Ni/Fe_2_O_3_ ND-MoS_2_ solution was drop casted onto a carbon fiber paper electrode. Then, the electrode was immersed in a 0.5 m H_2_SO_4_ solution, and the electrochemical leaching between 0.1 V to −0.4 V was performed for 50 cycles. The electrode was then dipped into an ethanol solution and sonicated for 30 min. The suspension was separated after being left to stand for 6 h. The entire process was repeated twice.

### Synthesis of Co NPs/MoS_2_ by self-assembly

Co NPs were obtained by a standard air-free procedures^45^. In the typical synthesis, 0.6 g of octadecylamine (ODA, Technical Grade, Acros Organics) and 0.2 g of TOPO were degassed with argon, and then 15 mL of DCB was added under argon. The resulting solution was heated to 182 °C, and 0.45 g Co_2_(CO)_8_ dissolved in 3 mL of DCB was rapidly injected into the hot solution. After 5 min heating at 182 °C, a black colloidal solution was obtained. The particles were isolated by centrifugation and washed with alcohol. Then, the obtained Co NPs and MoS_2_ solution with an appropriate mass ratio were mixed together in a 20 mL conical flask. The reaction system was degassed with argon for at least 20 min. Then, it was ultrasonically treated for 24 h in a thermostat reaction system with a constant temperature of 4 °C, power of 800 W and frequency of 40 KHz. The final product was collected by extraction from the solution, washed several times with water to remove the possible ions and organic component, and finally suspended in argon-saturated water for storage.

### Synthesis of Co NPs/MoS_2_ by in situ growth

For the Co NPs/MoS_2_ synthesized by a refined in situ growth method^46^, 0.1 mg of MoS_2_ was suspended in 10 mL water, followed by adding 0.03 mg of cobalt (II) chloride hexahydrate (CoCl_2_·6H_2_O, Technical Grade, Sigma Aldrich). Then, 5 mL of freshly prepared sodium borohydride (NaBH_4_, Technical Grade, Adamas-beta Chemical Co.) solution was injected. The solution was shaken for 5 s and stayed for aging. After 1 h reaction, a black colloidal solution was obtained. The nanostructures were isolated by centrifugation and washed with water. Then, the obtained Co NPs and MoS_2_ solution with an appropriate mass ratio were mixed together in a 20 mL conical flask. The reaction system was degassed with argon for at least 20 min. Then, it was ultrasonically treated for 24 h in a thermostat reaction system with a constant temperature of 4 °C, power of 800 W and frequency of 40 kHz. The final product was collected by extraction from the solution, washed several times with water to remove the possible ions and organic component, and finally suspended in argon-saturated water for storage.

### Synthesis of Co clusters/MoS_2_

Co clusters/MoS_2_ was synthesized according to a refined reported procedure^47^. In a typical synthesis, 24.7 mg of (NH_4_)_6_Mo_7_O_24_·4H_2_O, 53.2 mg of CH_4_N_2_S and 0.3 mg of CoCl_2_·6H_2_O were dissolved in 20 mL of Millipore water under vigorous stirring to form a homogeneous solution. The solution was then transferred into a 30 mL Teflon-lined stainless-steel autoclave and maintained at 220 °C for 18 h before the reaction system was allowed to cool to room temperature. The final product was washed several times with water and ethanol to remove any possible ions and finally suspended in ethanol.

### Materials characterization

TEM, HRTEM, and energy-dispersive X-ray (EDX) spectroscopy were performed on the FEI Tecnai G^2^ F20 TEM operating at 200 kV. The samples were prepared by dropping ethanol dispersions of the samples onto 300 mesh carbon-coated copper grids and then evaporating the solvent. Fast Fourier transform (FFT) masked contrast refined HRTEM images were obtained using Gatan digital micrograph software. HRTEM images were acquired using a JEOL-ARM200F TEM operated at 200 kV. The attainable spatial resolution of the microscope was 78 pm with a probe spherical-aberration corrector. High-angle annular dark-field (HAADF) images were acquired with an illumination semi-angle of 25 mrad and probe current of 100 pA. The dwell time for image acquisition was set at 10 μs per pixel to ensure a desirable signal-to-noise ratio. The collection angles for the HAADF images were fixed at 90–250 mrad. To obtain high-quality scanning transmission electron microscopy (STEM) images with atomic resolution, SA Co-D 1T MoS_2_ was pre-treated at 80 °C in a vacuum oven for 4 h to remove any organic ligands on its surface. The high-resolution STEM simulations were performed using the multi-slice JEMS software (copyright P.|A. Stadelmann, EPFL, Switzerland). A supercell of ~15 × 8 Å was built using the coordinates supplied in Fig. 1d. The pixel sampling used for the supercell was 2048 × 2048. The electron optical parameters used in the simulation were consistent with experimental conditions, including an electron energy of 200 kV, spherical-aberration coefficient of 78 pm, illumination semi-angle of 25 mrad and probe current of 100 pA. The FFT-filtered images were obtained by transposition of the HRTEM images into reciprocal space. To form the FFT patterns, reciprocal spots from the patterns (typically-type reflections) were selected, masked with a five pixel filter, and then transformed into real space with inverse FFT. The powder XRD experiments were conducted on a Bragg-Brentano diffractometer (D8-tools, Germany), and the source was a Cu-Kα line at 0.15418 nm. The scanning speed was set to 8° per min. Inductively coupled plasma mass spectrometry (ICP-MS) data were determined using an ELAN 9000/DRC ICP-MS system (Perkinelmer, USA). X-ray photoelectron spectroscopy (XPS) was performed using an ESCALAB-250 instrument (Thermo Fisher Scientific, USA), performed with a monochromatic Al-Kα (1486.6 eV) radiation source and a hemisphere detector with an energy resolution of 0.1 eV. Peak positions were all corrected by the C 1 s spectrum at 284.8 eV. Atomic force microscope images were obtained by the deposition of sample on freshly cleaved mica and investigated by using tapping mode in air (Dimension Icon, Veeco Instruments/Bruker, Germany). The Raman measurements with an excitation laser line of 532 nm were performed using a WITEC alpha 300 R Confocal Raman system and power of <0.1 mW to prevent the phase transition during measurement under ambient conditions.

### Electrochemical measurements

The electrochemical measurements were performed using a glassy carbon (GC) rotating disk electrode (RDE, Pine research instrumentation) connected to a CHI760D potentiostat (Shanghai, Chenhua Co., China) in a three-electrode cell. The working electrode (WE) was prepared by loading the ink containing 20 μg of the catalyst onto a GC RDE (3 mm in diameter). An Ag/AgCl (KCl saturated) electrode and graphite rod (3 cm in diameter) were used as the reference and counter electrodes, respectively. All potentials were calibrated to the RHE by the equation:

1 $${\mathit{E}}_{{\mathrm{RHE}}} \, = {\mathit{E}}_{{\mathrm{Ag}}/{\mathrm{AgCl}}} + 0.059\,{\mathrm{pH}} + 0.197$$

LSV was conducted in Ar-saturated 0.5 m H_2_SO_4_ solution between 0.1 and −0.6 V vs RHE to investigate the HER activity. The electrochemical impedance measurements were performed in the frequency range from 100 kHz to 0.1 Hz at an overpotential of 250 mV. All the impedance data were fitted by a simplified Randles circuit to extract the series and charge-transfer resistance. CV was conducted between 0.1 and −0.3 V vs RHE at 50 mV s^−1^ to investigate the cycling stability. A long-term stability test was recorded by taking a chronoamperometric curve at a constant overpotential of 150 mV. The onset overpotential was determined based on the potential when the current density reached 10 mA cm^−2^. All data are presented without IR-compensation. All the electrochemical tests were performed at room temperature.

### XAFS measurements

X-ray absorption spectra were collected at Beijing Synchrotron Radiation Facility (BSRF) on beamline 1W1B and Shanghai Synchrotron Radiation Facility (SSRF) on beamline BL14W1. The storage ring is operated at electron energy of 2.5 GeV with a beam current of 250 mA. A Si (111) double-crystal monochromatic was applied. The beam size used at the sample position was ~900 × 300 μm^2^. All the data were collected at ambient temperature applied in the transmission mode. Curve fitting and data analysis were performed with Artemis and IFEFFIT software^48,49^. The energy resolution (ΔE/E) for the incident X-ray photons was estimated to be 2 × 10^−4^. Conventional transmission mode was adopted for the Co, Ni and Fe K-edge EXAFS measurements. To ascertain the reproducibility of the experimental data, at least two scan sets were collected and compared for each sample.

### XAFS analysis

Each sample of XAS data was aligned, and processed using the Athena program. Spectra were baseline corrected using a linear pre-edge function between −200 and −50 eV and normalized using a linear or quadratic function between 150 and 700 eV, including a flattening function in the post-edge region. The XAFS signal was isolated from the adsorption edge background using a fit to a cubic spline with nodes defined by the AUTOBKG function in IFEFFIT, with a *k*-weight of 3 and with the Rbkg parameter set to 1. Fourier transformations of *k*^*3*^-weighted spectra were using a Kaiser–Bessel window with a 1 Å^−1^ sill width. The magnitude parts of the Fourier transformed spectra are shown throughout this manuscript with a radial distance scale that is not corrected for phase shift.

For EXAFS fitting, theoretical scattering paths were calculated with FEFF6 using Artemis. All EXAFS spectra were fit for distances (ΔR), CN, and mean-square displacement of interatomic distance (*σ*^2^) using the Artemis interface with a fixed amplitude reduction factor (S_0_^2^) of 0.707 ~ 1.000. The parameters such as interatomic distance (*R*), coordination number (CN), the difference in threshold energy (ΔE_0_) and Debye–Waller factor (*σ*^2^) were first established with reasonable guesses, and then were fitted in R-space. The error in the overall fits was determined using the R-factor, the goodness-of-fit parameter, in which R-factor = Σ(χ_data_ – χ_fit_)^2^/Σ(χ_data_)^2^ and good fits occur for R-factor < 0.05.

### Mechanism of phase transition of MoS_2_

The first-principle calculations about structural stability and phase transition were carried out by using accurate frozen-core full-potential projector augmented-wave pseudopotentials method on the basis of DFT, as implemented in the VASP code^50–52^. The 3d and 4 s electrons of Co atom, 4p, 4d, and 5 s electrons of Mo atom, and 3 s and 3p electrons of S atom were treated as the valence electrons. We used the generalized gradient approximation (GGA) with Perdew-Burke-Ernzerhof (PBE) parameterization for the exchange and correlation potential^53^. A kinetic energy cutoff of 500 eV for the plane wave expansion and a Monkhorst-Pack grid with a k-point spacing of 0.01 Å^−1^ were found to be sufficient to ensure that the total energy was converged at 1 meV/atom level. The slab model with a vacuum layer larger than 20 Å was used to avoid the artificial coupling between the periodically repeated cells along z direction. The structures were fully optimized by using the conjugate gradient algorithm until the maximum energy difference converge to 10^−6^ eV. The effect of spin polarization was considered in our calculation.

### Hydrogen binding free-energy calculations

All theoretical calculations about hydrogen-binding free energy were performed using DFT by DMOL3 code^54,55^. In the DFT calculations, the all-electron Kohn–Sham wave functions were expanded in the double numerical polarized atomic orbital basis, and GGA with PBE for describing the exchange and correlation energy was employed. Effective core potentials were considered during calculations. Self-consistent field procedure was done until the change of energy was > 10^−6^ Hartree, and the geometrical optimization of the structure was done with an energy convergence criterion of 10^−5^ Hartree. Long range nonlocal effects were taken into account by applying semi-empirical dispersion-correction approach through the DFT-D scheme with Grimme parameters^56^. In our models, the experimental in-plane lattice constant of 3.16 Å for the in-plane unit cell of MoS_2_ monolayers has been used. A vacuum region of 20 Å along the z direction is enough to ensure negligible interaction between the MoS_2_ monolayer and its periodic images. We used one cobalt dopant in 2 × 2, 3 × 3, 4 × 4, 5 × 5 and 6 × 6 MoS_2_ supercell as the models to present different Co doping coverage, corresponding to 8.33%, 3.70%, 2.08%, 1.33%, and 0.93%, respectively. The spin-unrestricted calculations were carried out. The Brillouin zone was sampled by Monkhorst-Pack 10 × 10 × 1, 7 × 7 × 1, 5 × 5 × 1, 4 × 4 × 1, and 3 × 3 × 1 *k*-point grids for 2 × 2, 3 × 3, 4 × 4, 5 × 5, and 6 × 6 MoS_2_ supercell, respectively^57^.

### Charge density difference calculations

The charge density difference (Δ*ρ*) for Co adsorbed MoS_2_ system was obtained using the following equation: Δ*ρ* = *ρ*(MoS_2_ + Co) − *ρ*(MoS_2_)  − *ρ*(Co), where *ρ*(MoS_2_ + Co), *ρ*(MoS_2_) and *ρ*(Co) are the total charge density of the Co adsorbed MoS_2_ system, MoS_2_ substrate and single Co atom, respectively. The charge density difference quantifies the redistribution of electron charge due to the interaction between adatom Co and MoS_2_ substrate.

## Supplementary information

Supplementary Information

Peer Review File

## Data Availability

The data that support the findings of this study are available from the corresponding authors upon reasonable request.
