# Supplementary material for: Single-atom cobalt array bound to distorted 1T MoS2 with ensemble effect for hydrogen evolution catalysis
Source: Nat Commun. 2019 Nov 19;10:5231. doi: 10.1038/s41467-019-12997-7 (PMC6863867; doi:10.1038/s41467-019-12997-7)
Supplement: Supplementary file 1 — Supplementary Information [file 41467_2019_12997_MOESM1_ESM.pdf]

# Supplementary Information

## Single-Atom Cobalt Array Bound to Distorted 1T-$MoS_2$ with Ensemble Effect for Hydrogen Evolution Catalysis

**Qi et al.**

## Supplementary Note 1

The morphology and structure of as-prepared Co NDs were further confirmed by the TEM and AFM images, as illustrated in Supplementary Figure 1. The statistics analysis of Co NDs shows an average thickness of 4.5 nm and diameter of 12 nm.

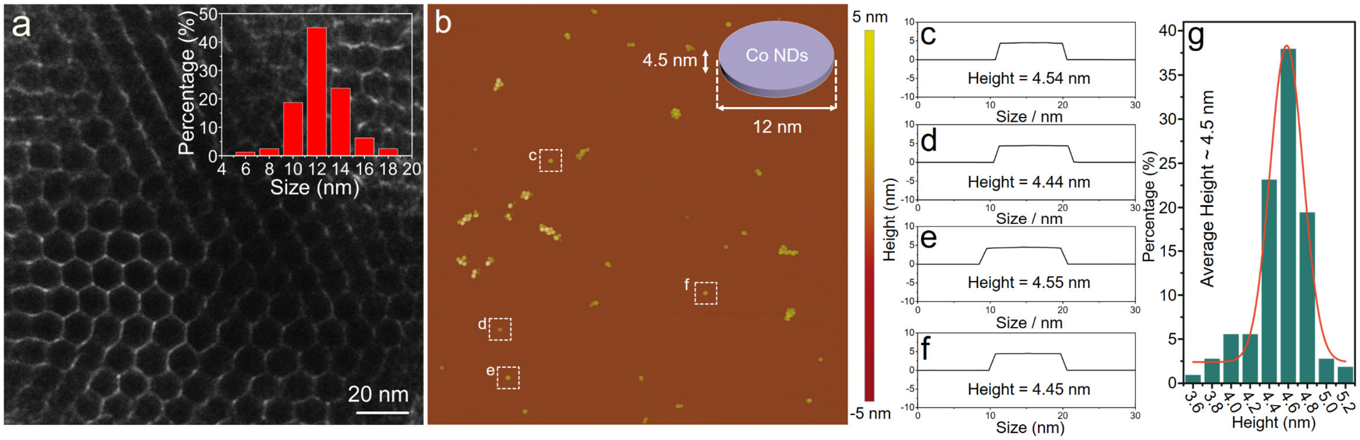


**Supplementary Figure 1.** Characterization of the Co nanodisks. **a,** TEM image of Co nanodisks loaded on carbon-coated copper grids and **b,** AFM image of Co nanodisks on mica. *Inset* of b shows the section analysis of the thickness of Co nanodisks.

## Supplementary Note 2

Supplementary Figure 2 shows that the loading amount is highly dependent on the sonication power, which is essential to form Co-S bond and thus triggers the phase transformation of $MoS_2$.

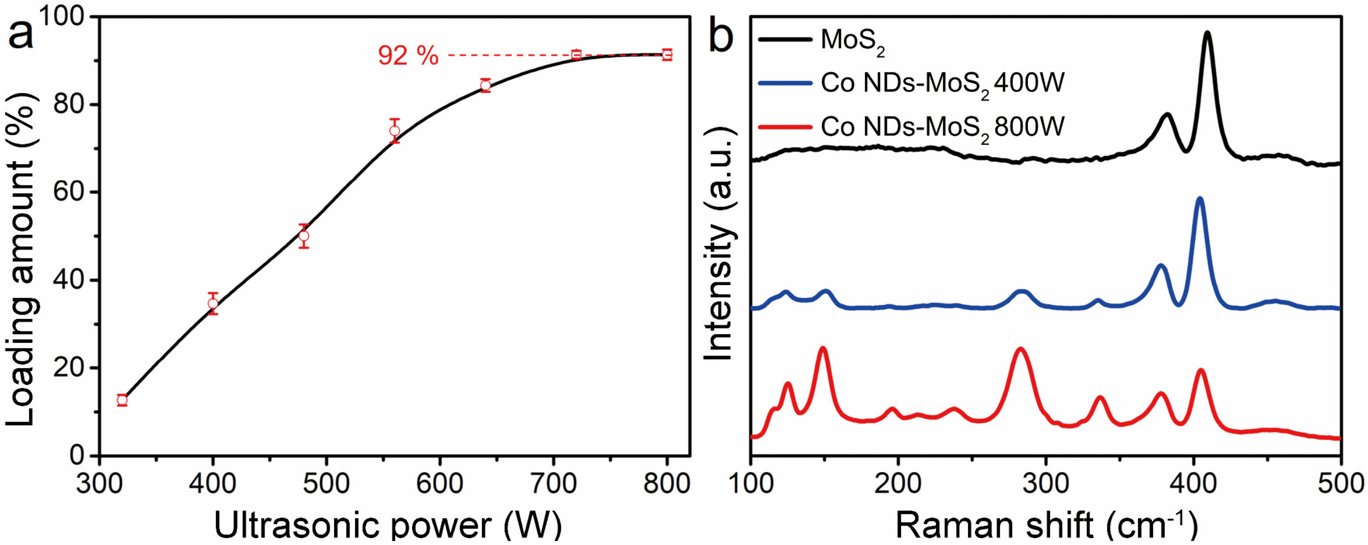


**Supplementary Figure 2. a,** Relationship curve between the ultrasonic power and the Co nanodisks loading amount for the Co NDs/$MoS_2$. **b,** Raman spectra of Co NDs/$MoS_2$ under different sonication power.

## Supplementary Note 3

Supplementary Figure 3 shows that elevating the reaction temperature will not achieve the effective assembly of Co nanodisks on $MoS_2$ nanosheets.

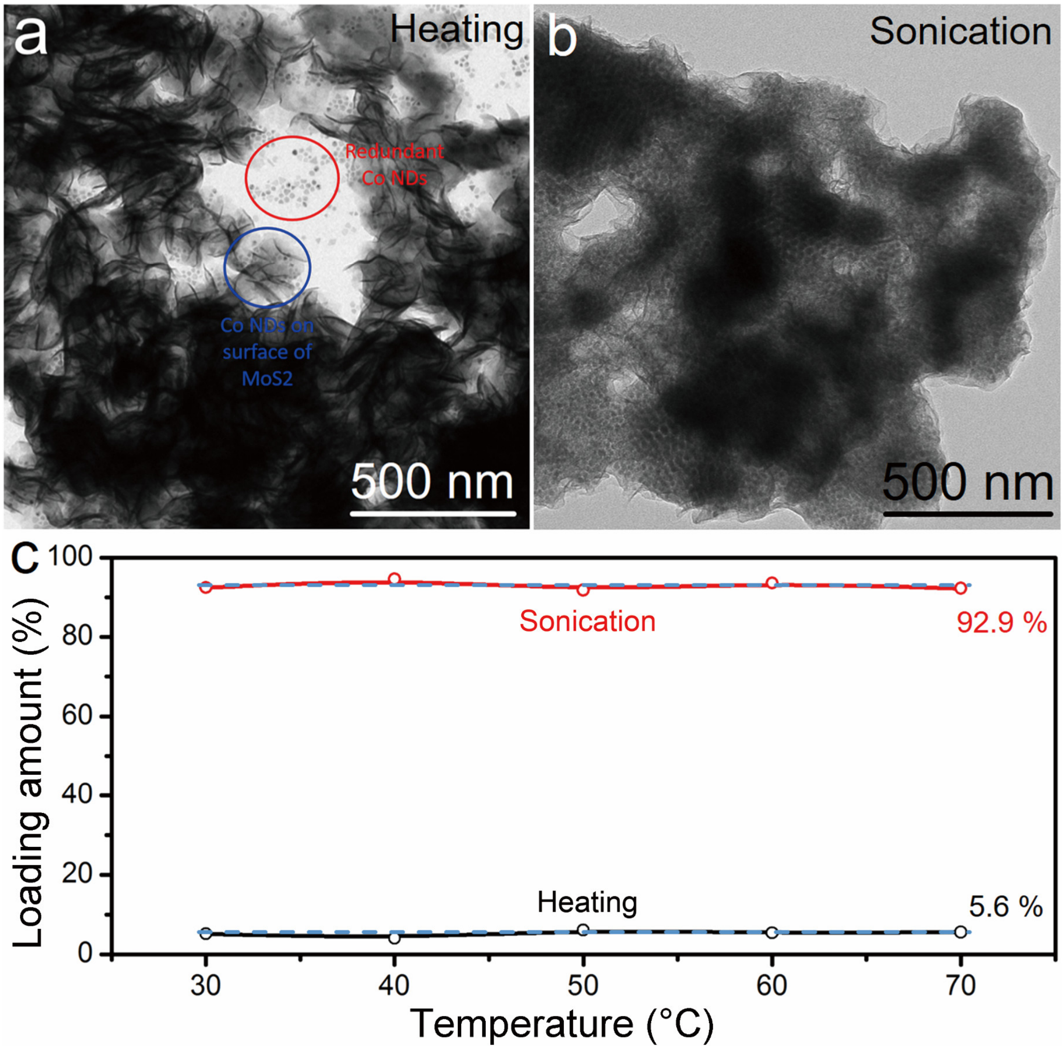


**Supplementary Figure 3. a-b,** TEM image of Co NDs/$MoS_2$ synthesized by heating and sonication. **c,** Relationship between reaction temperature and loading amount for Co NDs/$MoS_2$.

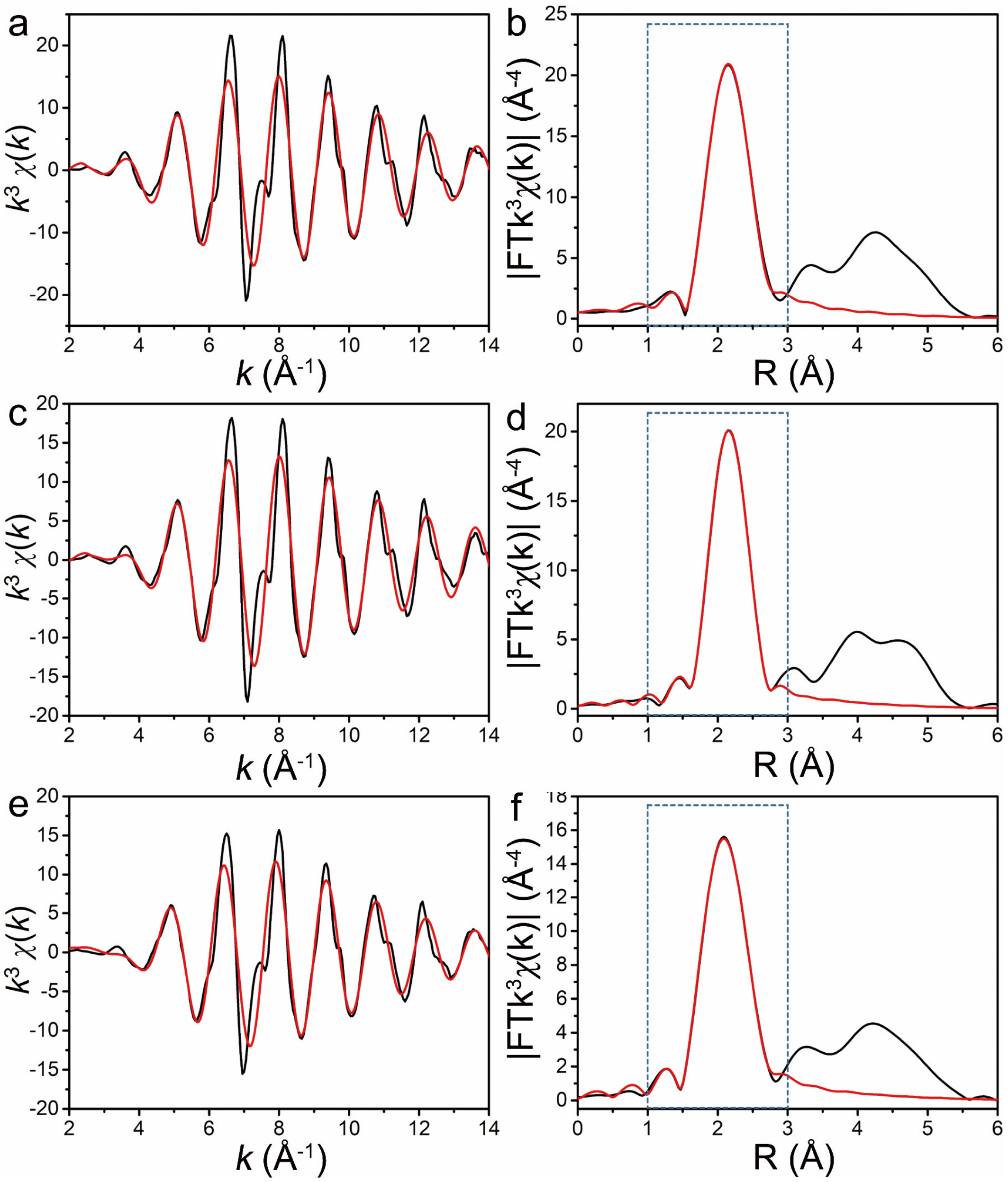


**Supplementary Figure 4**. Fourier-transformed magnitude of Co K-edge EXAFS spectra in *k* and R space for Co NDs/$MoS_2$ prepared by **a-b,** simply mixing, **c-d,** 2 h sonication and **e-f,** 10 h sonication. The experimental spectra are well simulated for all samples. The best-fitting parameters are shown in Supplementary Table 1.

## Supplementary Note 4

Supplementary Figure 4 and Supplementary Table 1 show that sonication can induce the formation of Co-S bond between Co NDs and $MoS_2$ nanosheets.

**Supplementary Table 1.** Co K-edge EXAFS curve Fitting Parameters obtained from Supplementary Figure 4 for the sonication induced bonding process of the Co NDs/$MoS_2$ heterostructures.[a]

| Sample | Path | CN | R(Å) | $\sigma^2$($\times 10^{-3}$Å$^2$) | $\Delta E_0$ (eV) | R,% |
|---|---|---|---|---|---|---|
| Co NDs/$MoS_2$ mixture[b] | Co-Co | 9.5 | 2.489 | 6.1 | 6.0 | 0.20 |
| Sonication 2h[c] | Co-Co | 6.9 | 2.496 | 5.0 | 7.9 | 0.10 |
| | Co-S | 0.4 | 2.201 | 3.6 | | |
| Sonication 10h[d] | Co-Co | 7.0 | 2.491 | 6.1 | 1.2 | 0.10 |
| | Co-S | 0.8 | 2.210 | 12.4 | | |

[a]N, coordination number; R, distance between absorber and backscatter atoms; $\sigma^2$, Debye-Waller factor to account for both thermal and structural disorders; $\Delta E_0$, inner potential correction; R factor (%) indicates the goodness of the fit. Error bounds (accuracies) that characterize the structural parameters obtained by EXAFS spectroscopy were estimated as N ± 20%; R ± 1%; $\sigma^2$ ± 20%; $\Delta E_0$ ± 20%. $S_0^2$ was fixed to 0.707 as determined from Co foil fitting. [b]Fitting range: $3.0 \leqslant k$ (Å$^{-1}$) $\leqslant 10.0$ and $1.0 \leqslant R$ (Å) $\leqslant 3.0$. [c]Fitting range: $3.0 \leqslant k$ (Å$^{-1}$) $\leqslant 10.0$ and $1.0 \leqslant R$ (Å) $\leqslant 3.0$. [d]Fitting range: $3.0 \leqslant k$ (Å$^{-1}$) $\leqslant 10.0$ and $1.0 \leqslant R$ (Å) $\leqslant 3.0$.

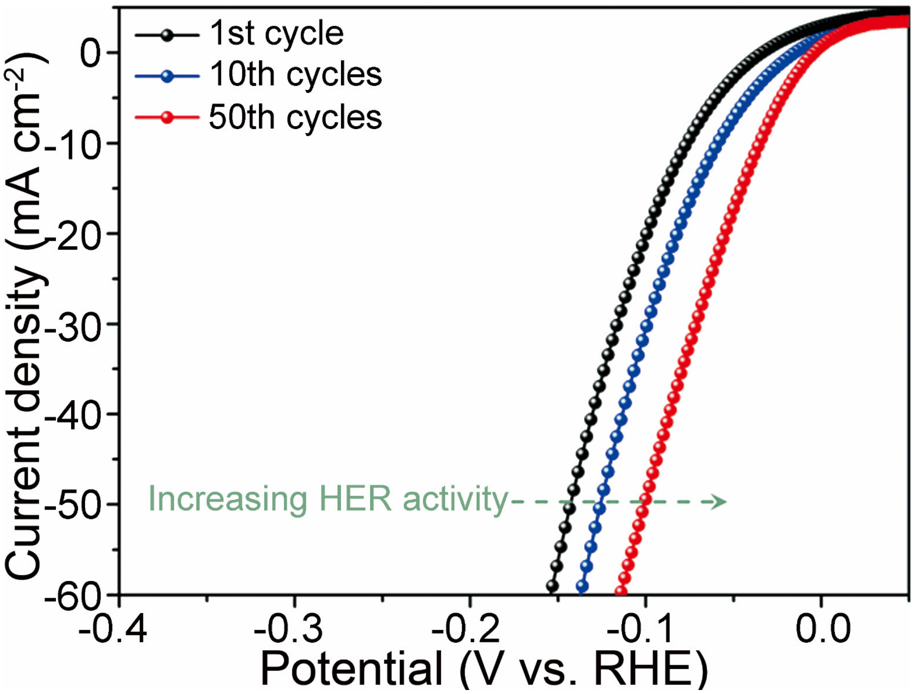


**Supplementary Figure 5.** HER polarization curves of the Co NDs/$MoS_2$ with different cycles between 0.1 and -0.4 V at a rate of 50 mV·$s^{-1}$.

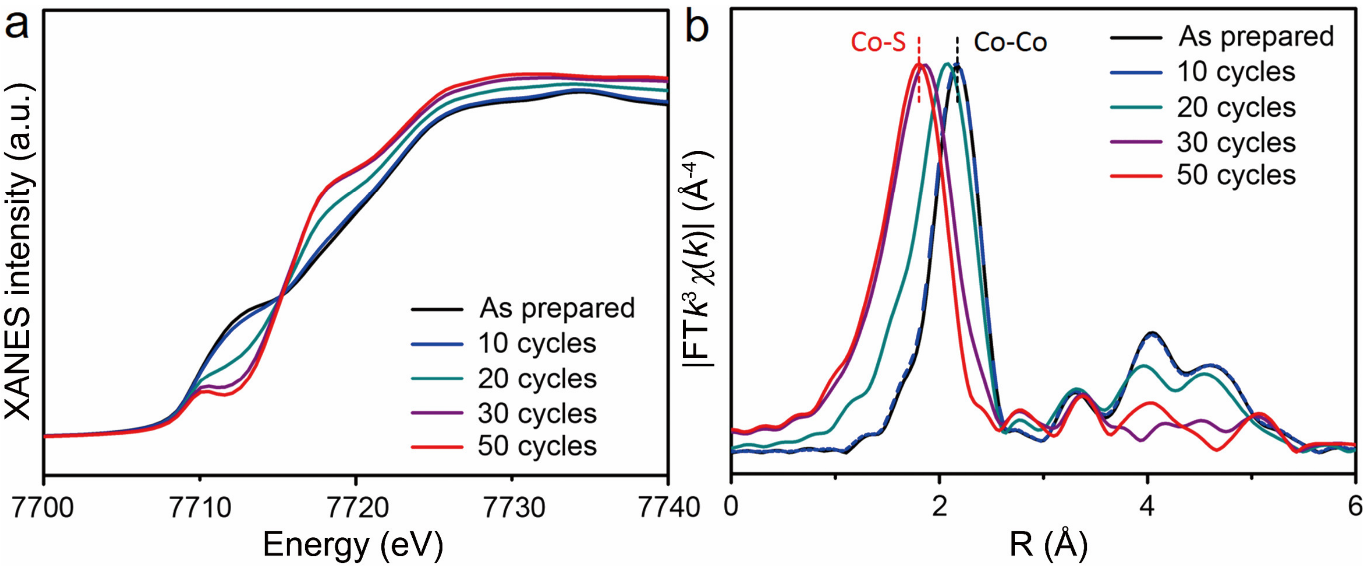


**Supplementary Figure 6.** *In-situ* Co K-edge X-ray absorption spectra during the electrochemical leaching for the preparation of SA Co-D 1T $MoS_2$ by 10, 20, 30, and 50 cycles. **a,** XANES spectra and **b,** EXAFS spectra.

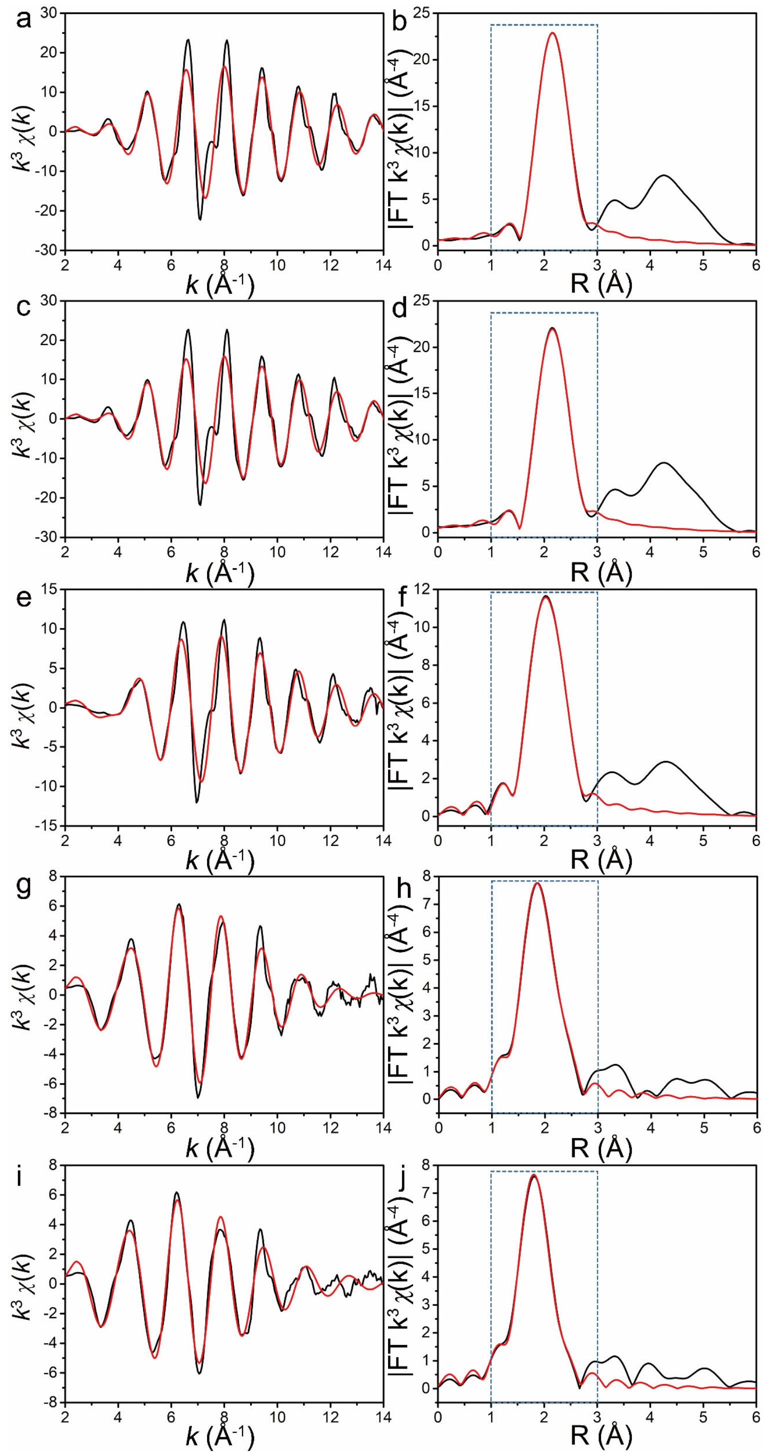


**Supplementary Figure 7.** Fourier-transformed magnitude of Co K-edge EXAFS spectra in k and R space for **a-b,** as prepared Co NDs/$MoS_2$ after 10 h sonication, **c-d,** Co NDs/$MoS_2$ electrochemical (EC) leaching for 10 cycles, **e-f,** Co NDs/$MoS_2$ electrochemical (EC) leaching for 20 cycles, **g-h,** Co NDs/$MoS_2$ electrochemical (EC) leaching for 30 cycles and **i-j,** Co NDs/$MoS_2$ electrochemical (EC) leaching for 50 cycles. Measured and calculated spectra are well matched for all samples. The best-fit parameters are shown in Supplementary Table 2.

## Supplementary Note 5

Supplementary Figure 5-7 and Supplementary Table 2 show that during the CV leaching process, the percentage of Co-Co bond was decreased whereas the percentage of Co-S bond increases, and finally transformed into pure Co-S bond in single atom form.[1]

**Supplementary Table 2.** Co K-edge EXAFS curve Fitting Parameters obtained from Supplementary Figure 7 for the electrochemical leaching process of the Co NDs/$MoS_2$.[a]

| Sample | Path | CN | R(Å) | $\sigma^2$($\times10^{-3}$Å$^2$) | $\Delta E_0$ (eV) | R,% |
|---|---|---|---|---|---|---|
| As prepared Co NDs/$MoS_2$[b] | Co-Co | 7.2 | 2.490 | 5.9 | 6.9 | 0.11 |
| | Co-S | 0.2 | 2.200 | 4.7 | | |
| 10 cycles[c] | Co-Co | 6.6 | 2.492 | 5.7 | 7.3 | 0.07 |
| | Co-S | 0.4 | 2.202 | 11.7 | | |
| 20 cycles[d] | Co-Co | 4.0 | 2.481 | 6.6 | -1.7 | 0.16 |
| | Co-S | 2.7 | 2.276 | 15.2 | | |
| 30 cycles[e] | Co-Co | 2.8 | 2.486 | 8.7 | -2.9 | 0.26 |
| | Co-S | 3.0 | 2.250 | 9.7 | | |
| 50 cycles[f] | Co-S | 3.6 | 2.260 | 9.1 | -2.0 | 0.28 |

[a]N, coordination number; R, distance between absorber and backscatter atoms; $\sigma^2$, Debye-Waller factor to account for both thermal and structural disorders; $\Delta E_0$, inner potential correction; R factor (%) indicates the goodness of the fit. Error bounds (accuracies) that characterize the structural parameters obtained by EXAFS spectroscopy were estimated as N ± 20%; R ± 1%; $\sigma^2$ ± 20%; $\Delta E_0$ ± 20%. $S_0^2$ was fixed to 0.707 as determined from Co foil fitting. [b]Fitting range: 3.0 ⩽ k (Å$^{-1}$) ⩽ 10.0 and 1.0 ⩽ R (Å) ⩽ 3.0. [c]Fitting range: 3.0 ⩽ k (Å$^{-1}$) ⩽ 10.0 and 1.0 ⩽ R (Å) ⩽ 3.0. [d]Fitting range: 3.0 ⩽ k (Å$^{-1}$) ⩽ 10.0 and 1.0 ⩽ R (Å) ⩽ 3.0. [e]Fitting range: 3.0 ⩽ k (Å$^{-1}$) ⩽ 10.0 and 1.0 ⩽ R (Å) ⩽ 3.0. [f]Fitting range: 3.0 ⩽ k (Å$^{-1}$) ⩽ 10.0 and 1.0 ⩽ R (Å) ⩽ 3.0.

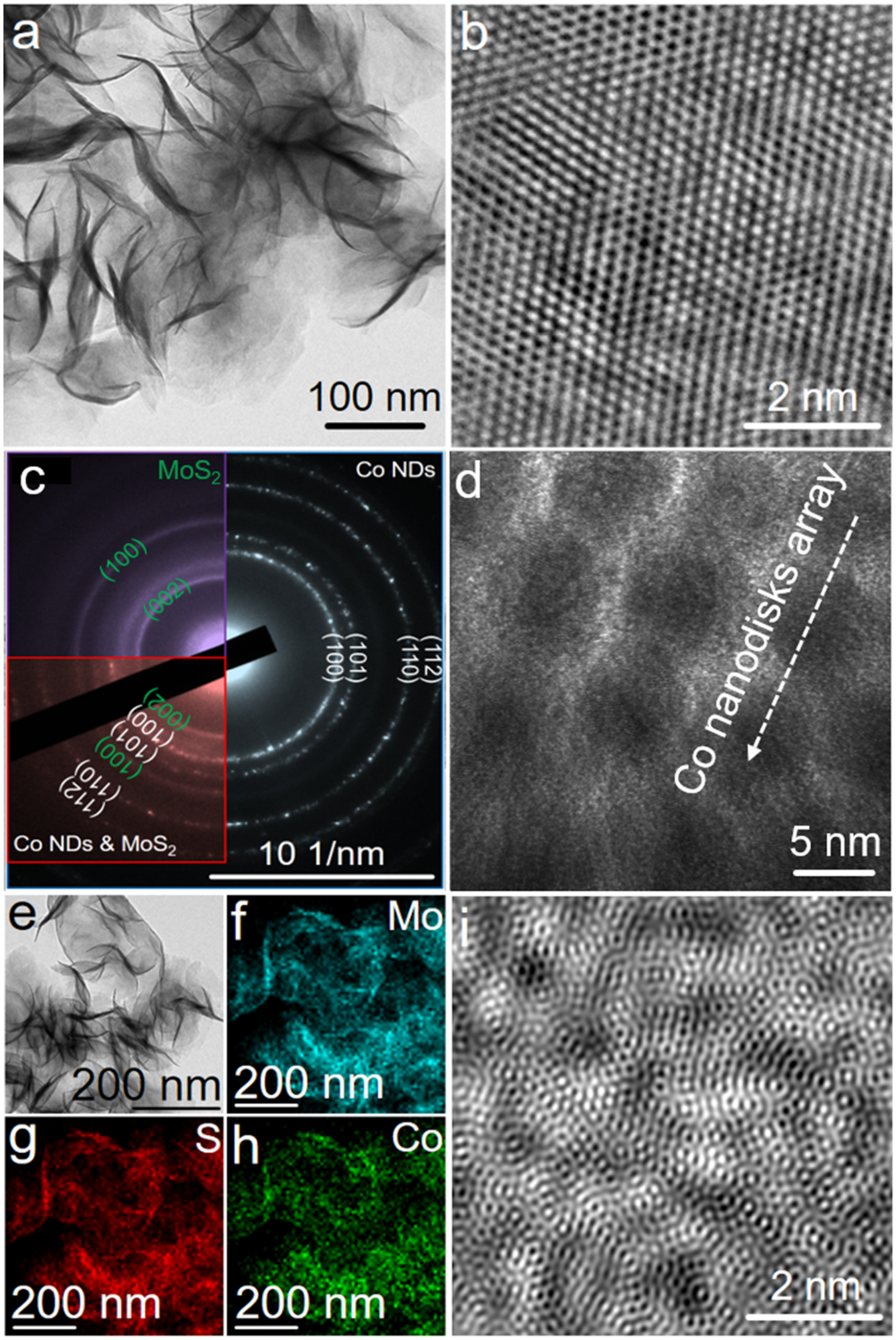


**Supplementary Figure 8.** Characterization of different nanostructures during the fabrication process for SA Co-D 1T $MoS_2$. **a-b,** TEM and HRTEM images of the $MoS_2$. **c,** SAED pattern of $MoS_2$, Co NDs and Co NDs/$MoS_2$. **d,** TEM images of the Co ND/$MoS_2$ shows that Co NDs were periodically arranged by magnetic effects among each other. **e-i,** TEM, EDX-mapping, and HRTEM images of the SA Co-D 1T $MoS_2$. No Co nanoparticles or clusters were observed in e and i.

## Supplementary Note 6

As can be seen from the XRD pattern, with the single atom Co modification, there is no obvious peak for the Co nanoparticles and clusters, which suggests that the Co is in the single atom condation.[2] Also, there is a very small shoulder peak below 10 degree, which can be aligned to the formation of disorder and the phase transition in the $MoS_2$ lattice.[3]

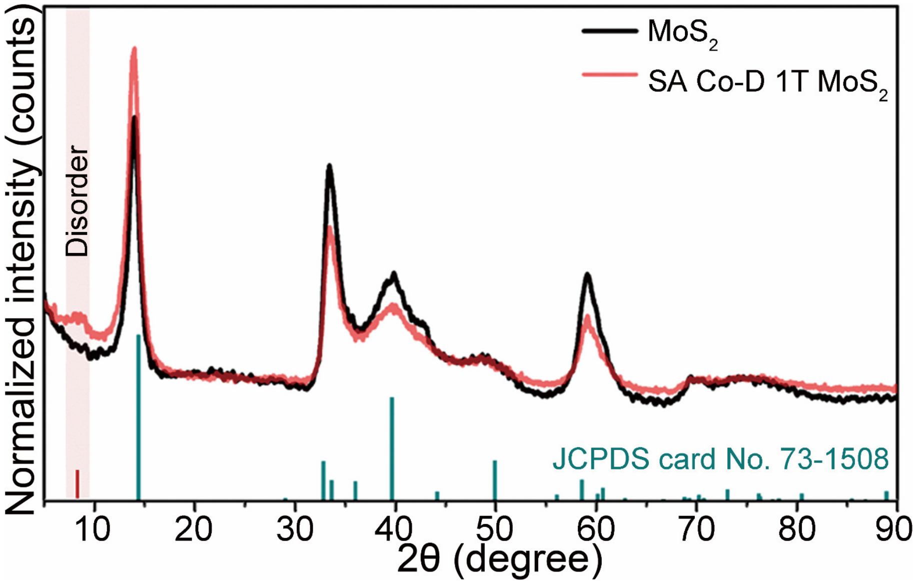


**Supplementary Figure 9.** XRD patterns of pristine $MoS_2$ and SA Co-D 1T $MoS_2$. The standard pattern of $MoS_2$ (JCPDS card No. 73-1508) is shown as a reference.

## Supplementary Note 7

The *Z*-contrast intensity in HAADF-STEM is $I \sim Z^{1.6\text{-}2.0}$ (*I* and *Z* are the image contrast and atomic number, respectively).[4] Therefore, Mo, Co and S atoms can be unambiguously discriminated with Mo (*Z*=42) being bright contrast and the two superposed S (*Z*=16) and Co (*Z*=27) atoms being dim contrast. However, the Co atoms in SA Co-D 1T $MoS_2$ show brighter contrast than the Mo atoms, indicating that the Co atoms in Supplementary Figure 10 should be located right above the Mo atoms.

The possibility of Moiré pattern is excluded for the disordered structure of SA Co-D 1T $MoS_2$ by the comparison of the experimental results of SA Co-D 1T $MoS_2$ and calculation results of the Moiré patterns when two layer or multilayer of $MoS_2$ aggregate with relative orientations from 0 to 90 degrees.[5,6]

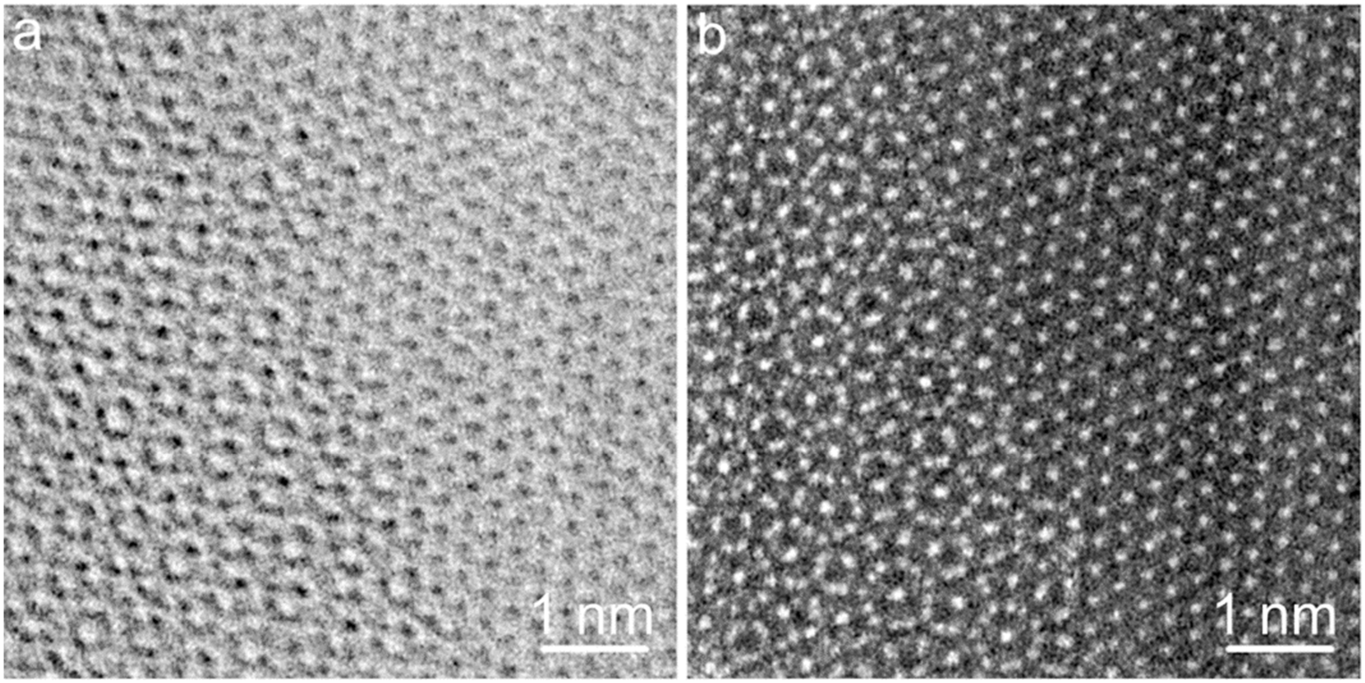


**Supplementary Figure 10.** Aberration-corrected STEM images of the SA Co-D 1T $MoS_2$. **a,** BF-STEM image and **b,** DF-STEM image.

## Supplementary Note 8

The size distribution in Supplementary Figure 11 reveals that the size of 100% Co species on $MoS_2$ is among 0.2 to 0.3 nm, demonstrating that Co exists exclusively as isolated single atoms.[7]

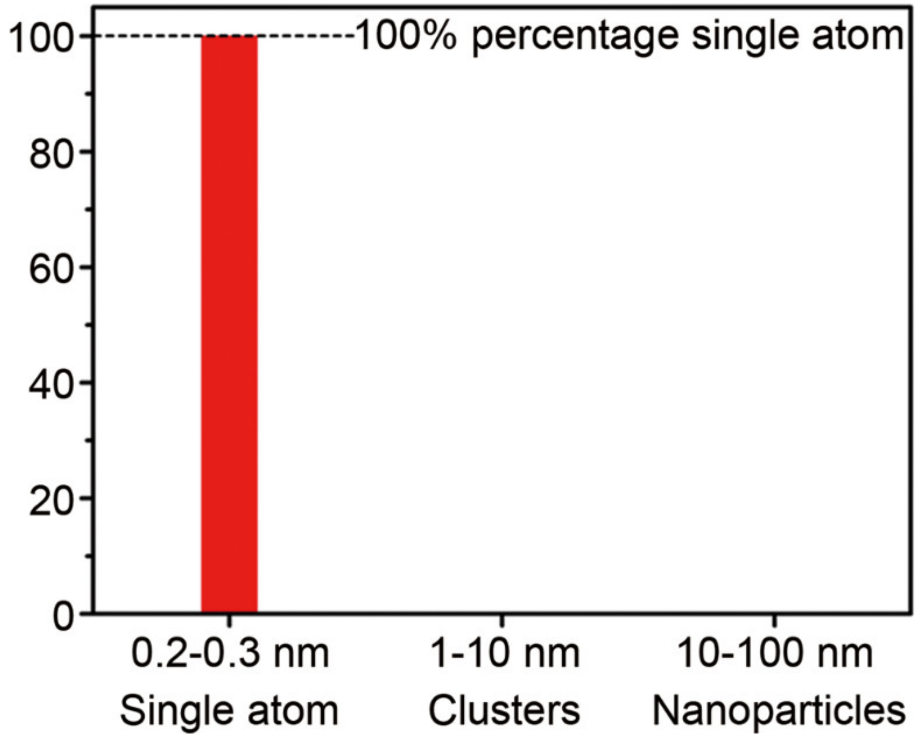


**Supplementary Figure 11.** Statistical data for the Co size distribution determined from the DF-STEM image.

## Supplementary Note 9

HAADF-STEM image of SA Co-D 1T $MoS_2$ revealed the Z-contrast of the site scattered across the basal plane labeled as Co&Mo is higher than that of the surrounding Mo and S sites. The *Z*-contrast nature of HAADF image implies that this is due to heavier constituent atom in the scanned atomic column. A very bright image feature appears on top of the middle Mo position. This can be attributed to the single Co atom sitting on top of the trigonal prismatic Mo atom (Mo atop site).[8,9]

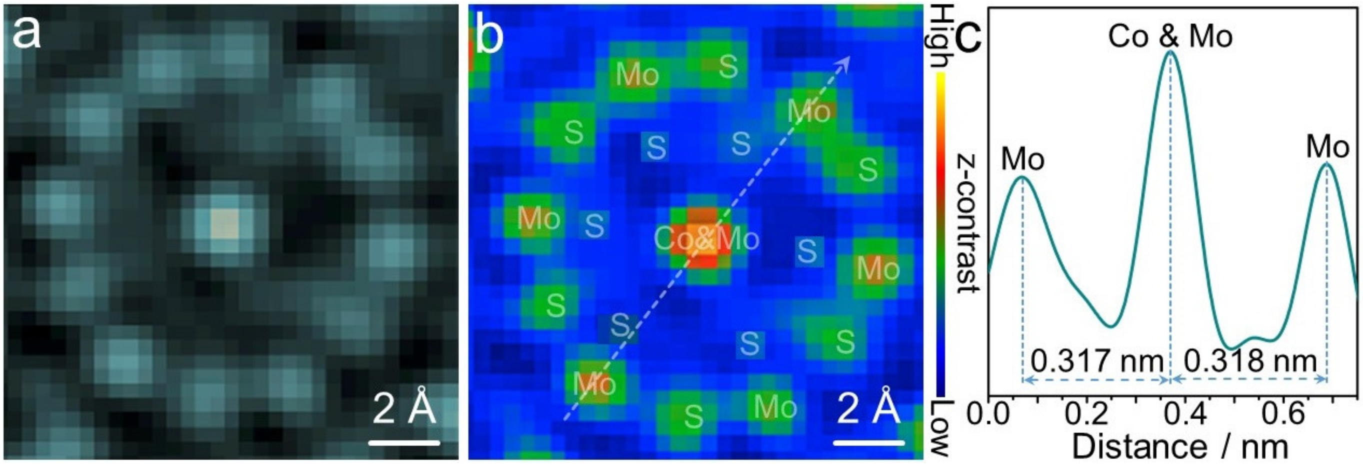


**Supplementary Figure 12.** Atomic structural analysis for a selected region on SA Co-D 1T $MoS_2$ catalyst. **a,** HAADF-STEM image. **b,** *Z*-contrast distribution mapping image, **c,** *Z*-contrast distribution cross-section image.

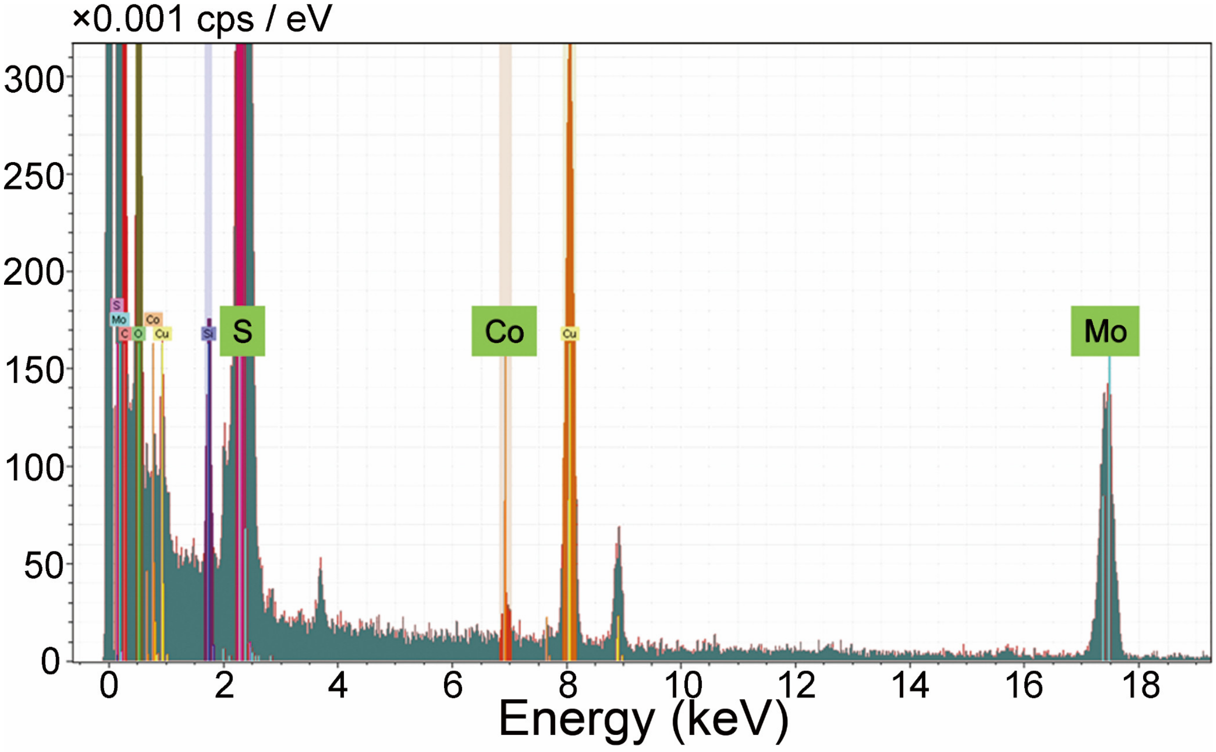


**Supplementary Figure 13.** EDX spectrum of the SA Co-D 1T $MoS_2$.

## Supplementary Note 10

We further determine the Co loading amount using the XAFS simulation. We used one cobalt dopant on 2×2, 3×3, 4×4 and 5×5 $MoS_2$ supercell as the models to present different Co doping coverage, corresponding to 8.33%, 3.70%, 2.08% and 1.33%, respectively. As can be seen from the simulation, both the pre-edge and the white line of the experimental data are close to the loading amount of 3×3 (3.70%). Therefore, the Co mass loading of 3×3 can be calculated as:

$$0.037\times59 / (0.037\times59 + 0.321\times96 + 0.642\times32) = 4.07\ \text{wt.\%}$$

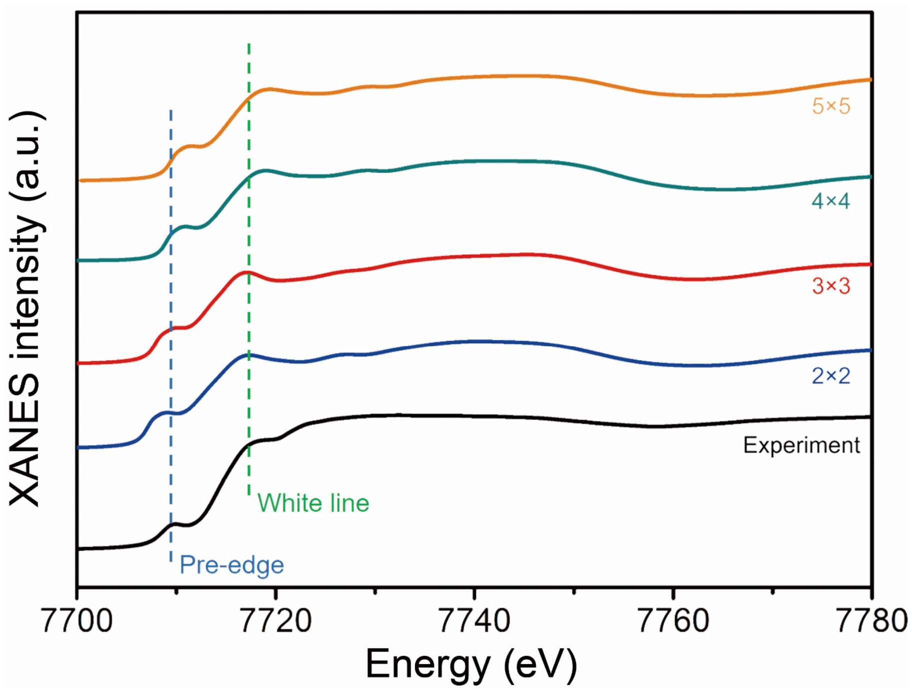


**Supplementary Figure 14.** XAFS spectrum simulation for different Co loading amount.

## Supplementary Note 11

The Ni NDs were synthesized according to the previous reported method and then characterized by TEM and AFM. The average diameter and thickness of the Ni NDs are 21 nm and 4.8 nm, respectively.

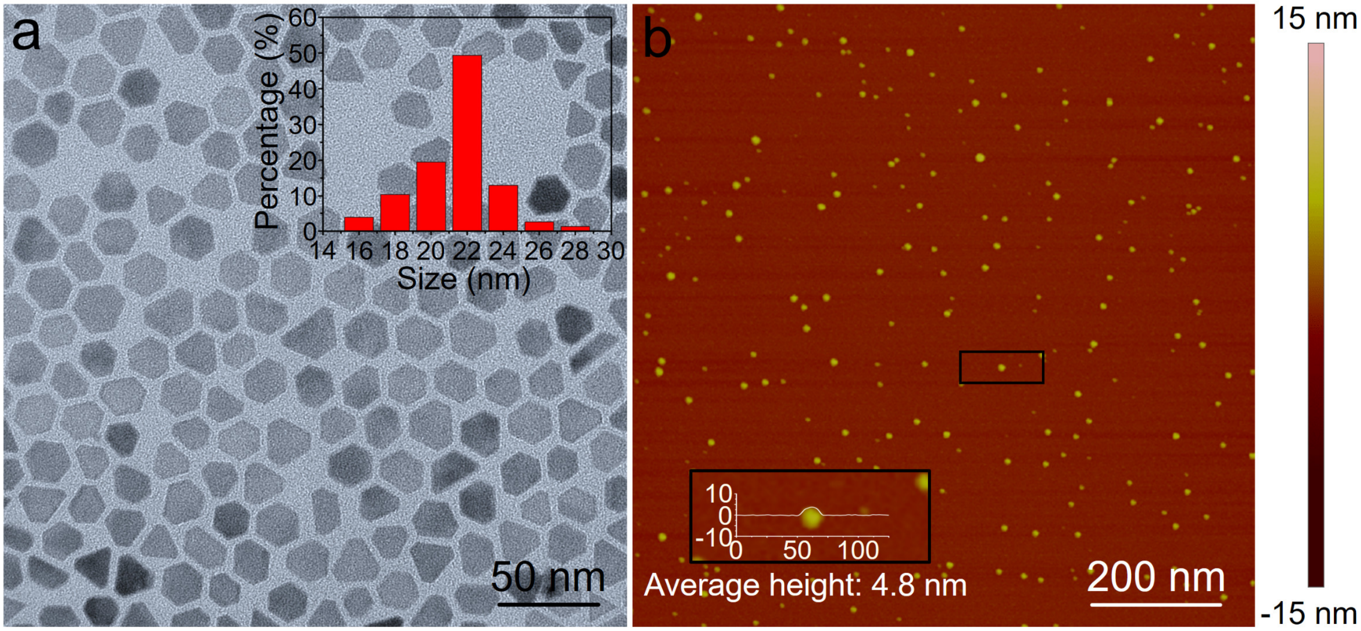


**Supplementary Figure 15.** Characterization of the Ni nanodisks (Ni NDs). **a,** TEM image and **b,** AFM image.

## Supplementary Note 12

The $Fe_2O_3$ NDs were synthesized according to the previous reported method and then characterized by SEM and TEM. The average diameter and thickness of the $Fe_2O_3$ NDs are 150 nm and 20 nm, respectively.

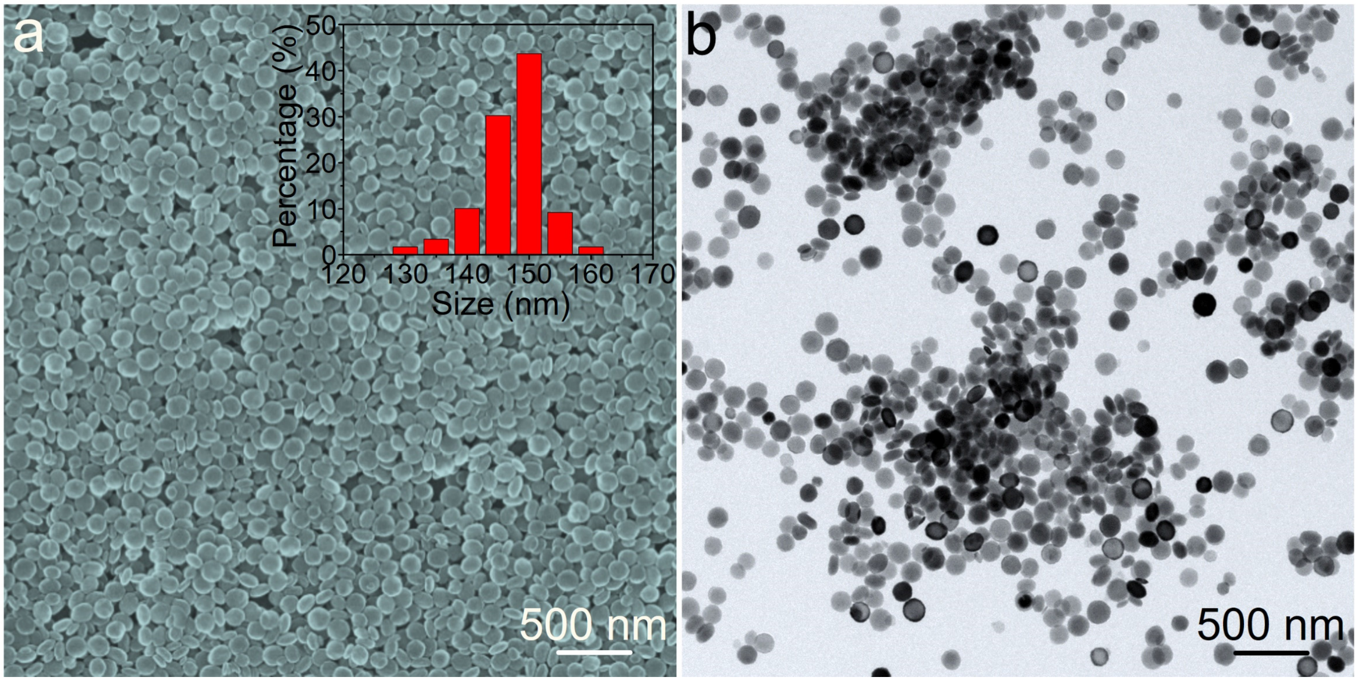


**Supplementary Figure 16.** Characterization of the $Fe_2O_3$ nanodisks ($Fe_2O_3$ NDs). **a,** SEM image and **b,** TEM image.

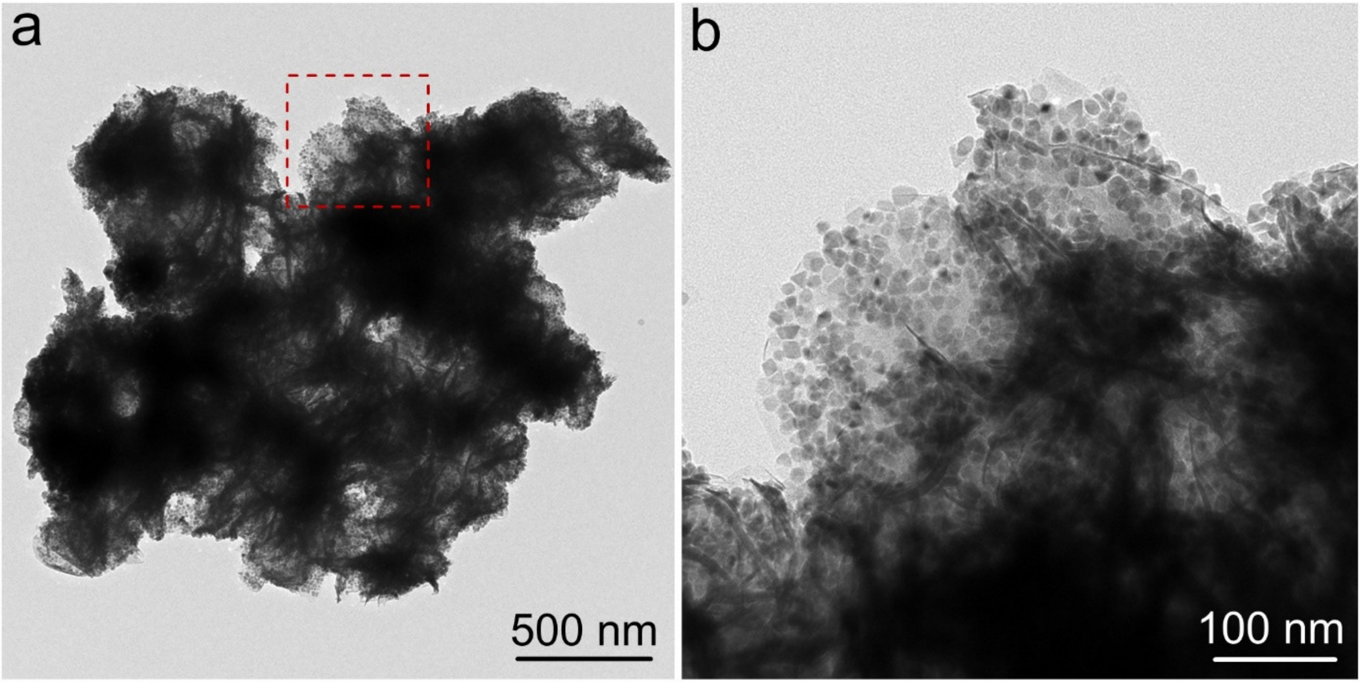


**Supplementary Figure 17.** TEM image of the Ni NDs modified $MoS_2$ nanosheets with different magnification.

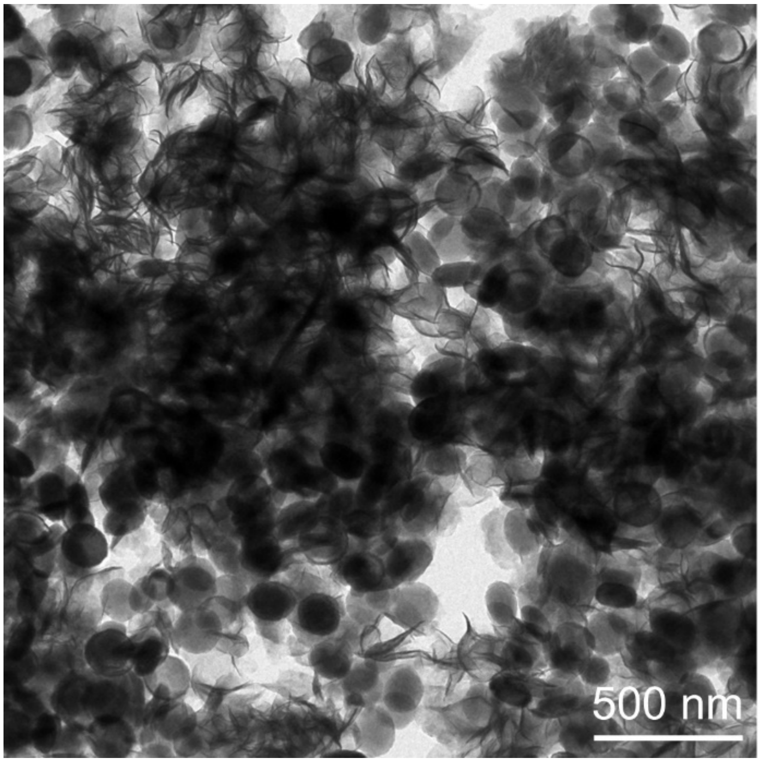


**Supplementary Figure 18.** TEM image of the $Fe_2O_3$ NDs modified $MoS_2$ nanosheets.

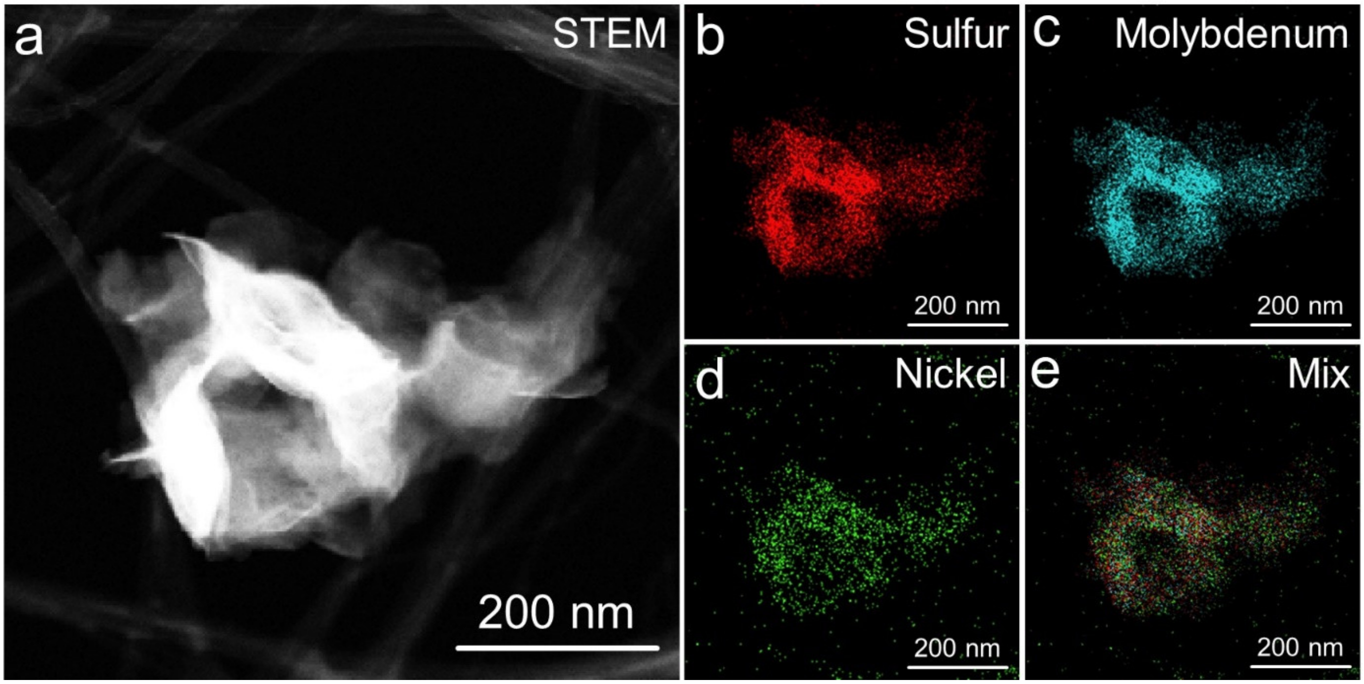


**Supplementary Figure 19. a,** HAADF-STEM and **b-e,** EDX-mapping images of the fresh prepared SA Ni-D 1T $MoS_2$.

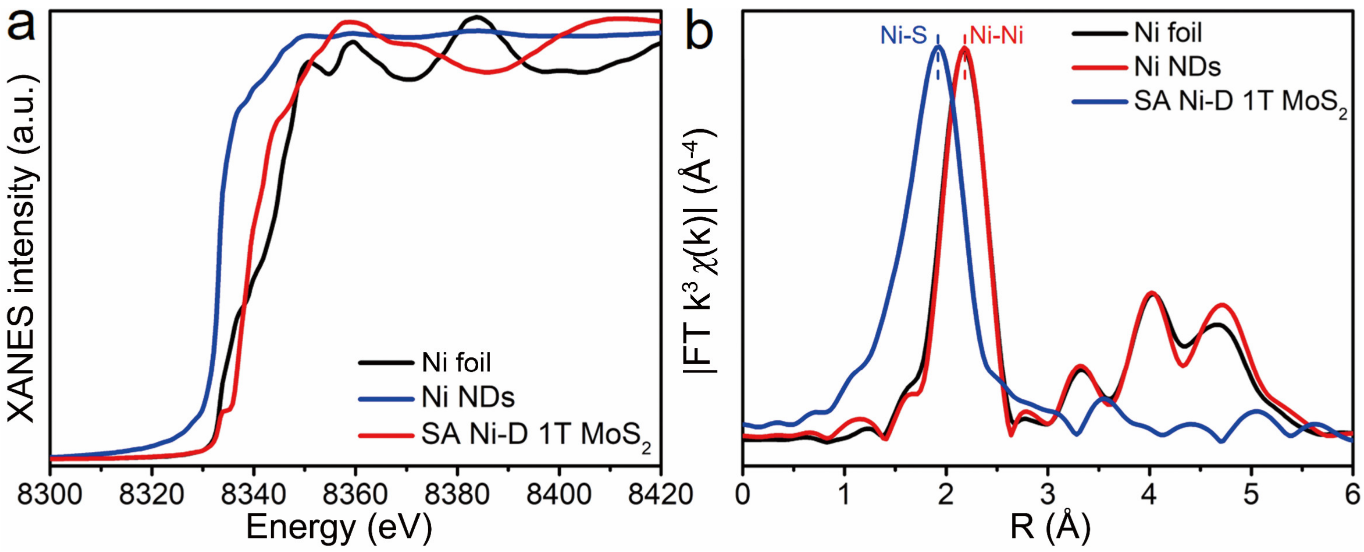


**Supplementary Figure 20.** Ni K-edge **a,** XANES and **b,** FT-EXAFS spectra of SA Ni-D 1T $MoS_2$, Ni NDs and Ni foil. The corresponding results confirm the successful synthesis of SA Ni-D 1T $MoS_2$.

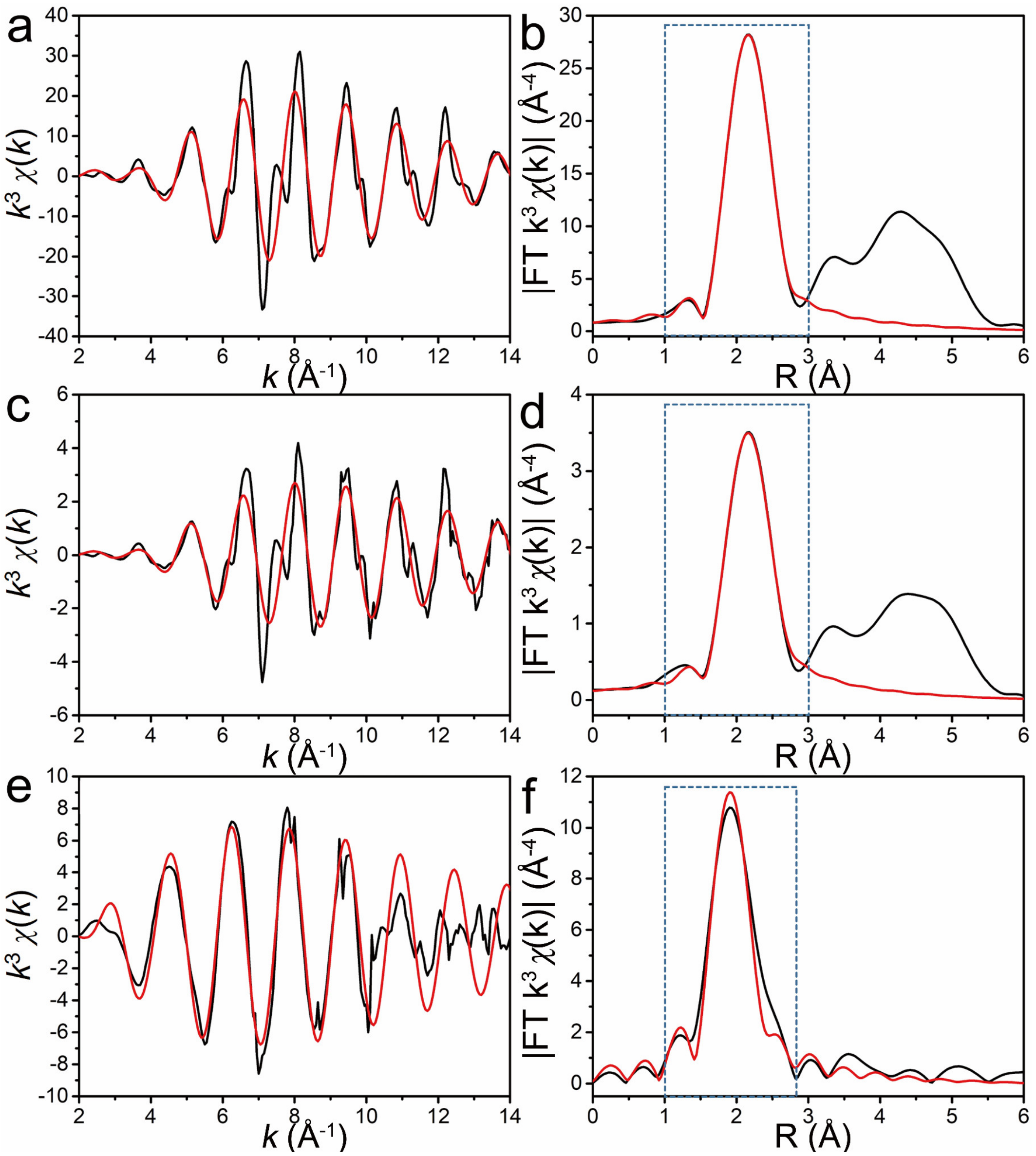


**Supplementary Figure 21.** Fourier-transformed magnitude of Ni K-edge EXAFS spectra in k and R space for **a-b,** Ni foil, **c-d,** Ni NDs, **e-f,** SA Ni-D 1T $MoS_2$. The measured and simulated spectra are well matched for all samples. The best-fit parameters are shown in Supplementary Table 3.

**Supplementary Table 3.** The Ni K-edge EXAFS curve Fitting Parameters obtained from Supplementary Figure 21 for Ni foil, Ni NDs and SA Ni-D 1T $MoS_2$.[a]

| Sample | Path | CN | R(Å) | $\sigma^2$($\times 10^{-3}$Å$^2$) | $\Delta E_0$ (eV) | R,% |
|---|---|---|---|---|---|---|
| Ni foil[b] | Ni-Ni | 12.0 | 2.483 | 6.2 | 7.8 | 0.1 |
| Ni NDs[c] | Ni-Ni | 12 | 2.480 | 4.0 | 7.3 | 0.3 |
| SA Ni-D 1T $MoS_2$[d] | Ni-S | 5.1 | 2.415 | 3.0 | 4.5 | 3.4 |

[a]N, coordination number; R, distance between absorber and backscatter atoms; $\sigma^2$, Debye-Waller factor to account for both thermal and structural disorders; $\Delta E_0$, inner potential correction; R factor (%) indicates the goodness of the fit. Error bounds (accuracies) that characterize the structural parameters obtained by EXAFS spectroscopy were estimated as N ± 20%; R ± 1%; $\sigma^2$ ± 20%; $\Delta E_0$ ± 20%. $S_0^2$ was fixed to 0.807 as determined from Co foil fitting. [b]Fitting range: 3.0 ⩽ k (Å$^{-1}$) ⩽ 10.0 and 1.0 ⩽ R (Å) ⩽ 3.0. [c]Fitting range: 3.0 ⩽ k (Å$^{-1}$) ⩽ 10.0 and 1.0 ⩽ R (Å) ⩽ 3.0. [d]Fitting range: 3.0 ⩽ k (Å$^{-1}$) ⩽ 10.0 and 1.0 ⩽ R (Å) ⩽ 2.8.

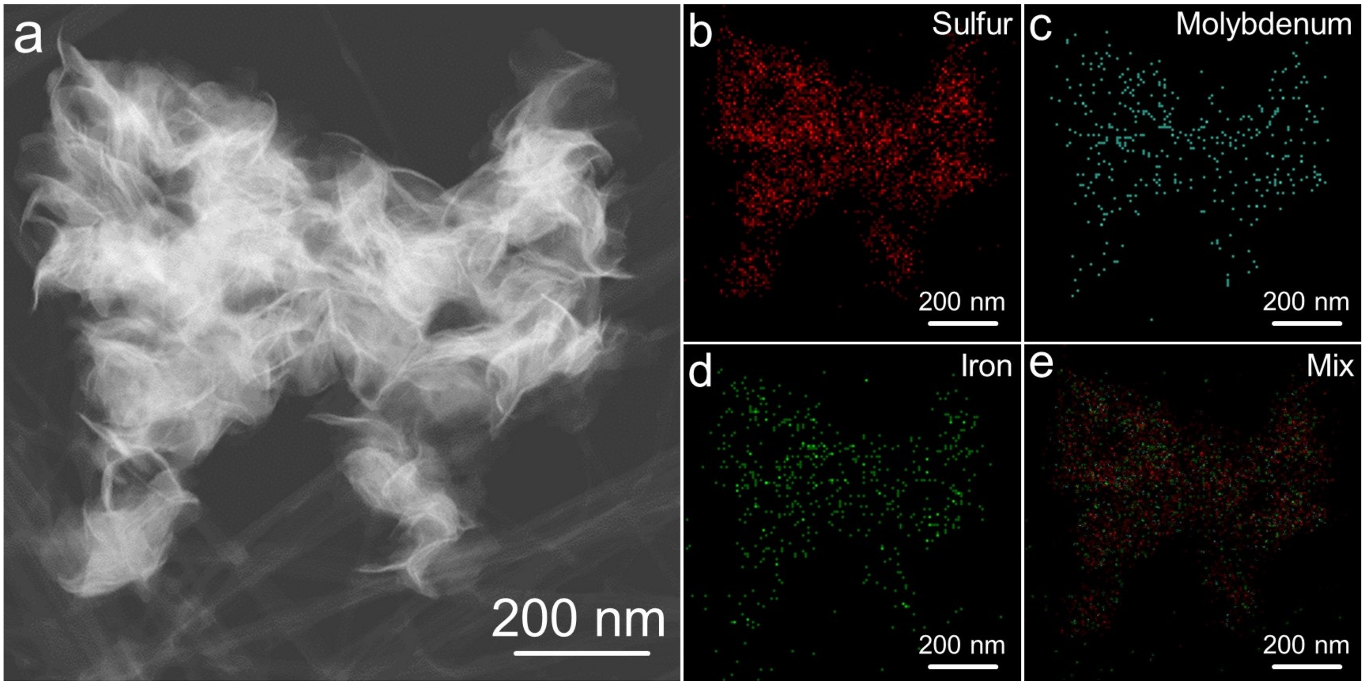


**Supplementary Figure 22. a,** HAADF-STEM and **b-e,** EDX-mapping images of the fresh prepared SA Fe-D 1T $MoS_2$ catalyst.

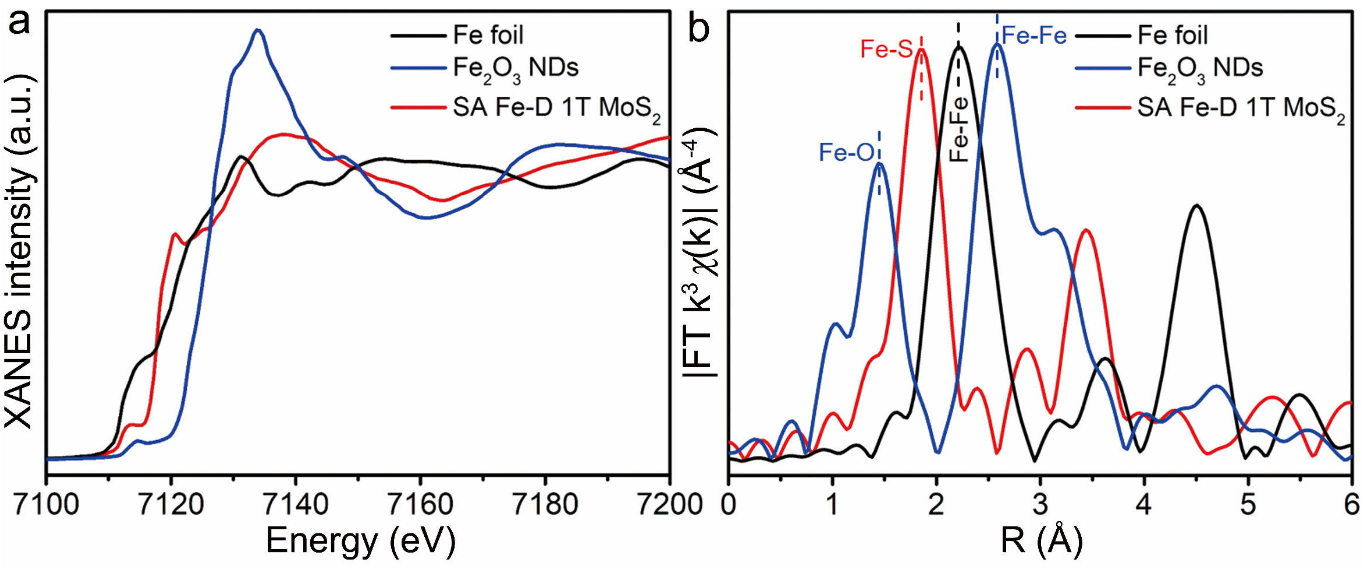


**Supplementary Figure 23.** Fe K-edge **a,** XANES and **b,** FT-EXAFS spectra of SA Fe-D 1T $MoS_2$, $Fe_2O_3$ NDs and Fe foil. The corresponding results confirm the successful synthesis of SA Fe-D 1T $MoS_2$.

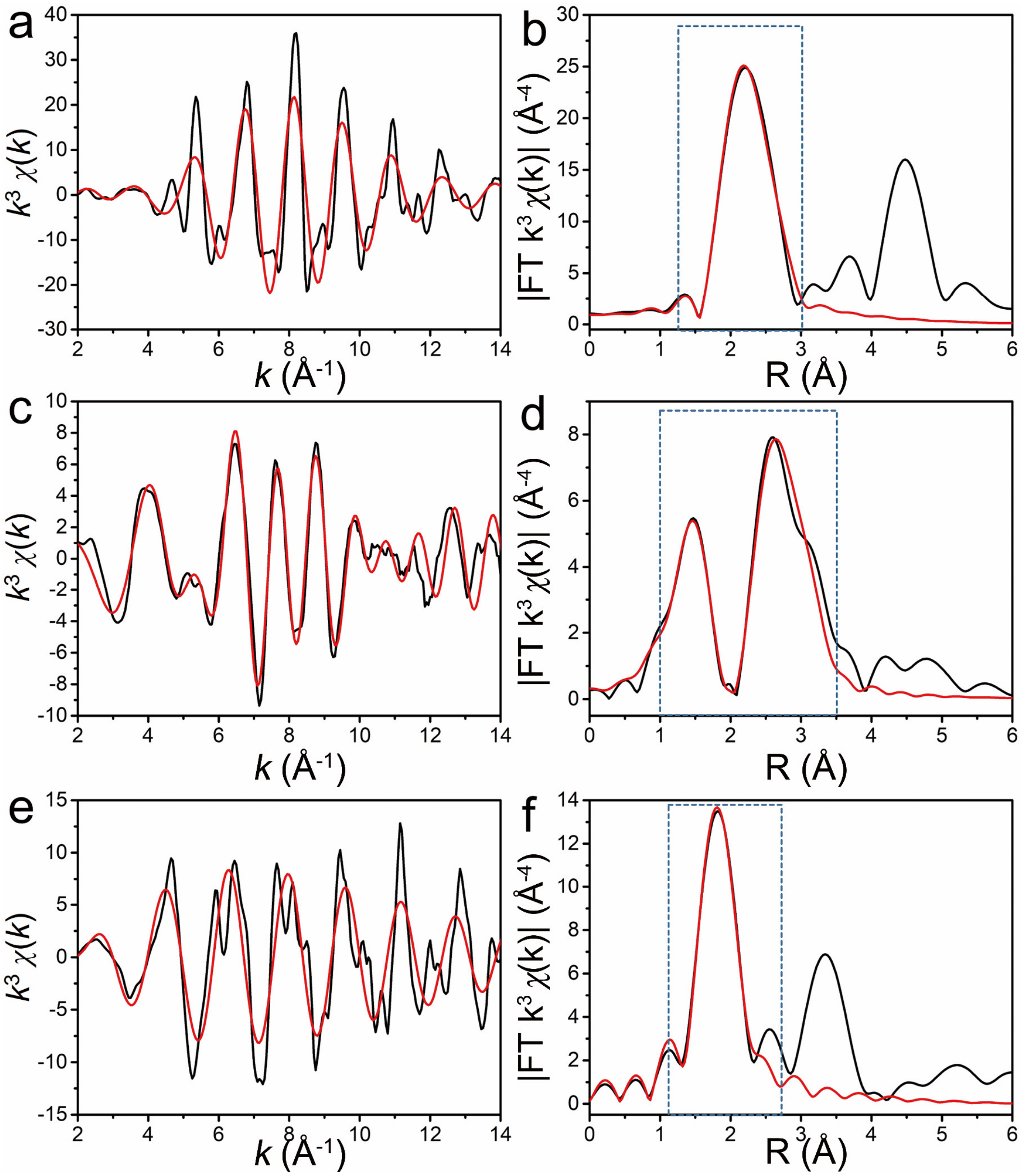


**Supplementary Figure 24.** Fourier-transformed magnitude of Fe K-edge EXAFS spectra in k and R space for **a-b,** Fe foil, **c-d,** $Fe_2O_3$ NDs and **e-f,** SA Fe-D 1T $MoS_2$. The measured and calculated spectra are well matched for all samples. The best-fit parameters are shown in Supplementary Table 4.

**Supplementary Table 4.** Fe K-edge EXAFS curve Fitting Parameters obtained from Supplementary Figure 24 for Fe foil, $Fe_2O_3$ NDs and SA Fe-D 1T $MoS_2$.[a]

| Sample | Path | CN | R(Å) | $\sigma^2(\times10^{-3}Å^2)$ | $\Delta E_0$ (eV) | R,% |
|---|---|---|---|---|---|---|
| Fe-foil[b] | Fe-Fe1 | 6 | 2.846 | 8.8 | 7.4 | 0.5 |
| | Fe-Fe2 | 8 | 2.481 | 6.8 | | |
| $Fe_2O_3$ NDs[c] | Fe-O1 | 2.4 | 1.915 | 6.1 | -6.0 | 1.4 |
| | Fe-O2 | 2.4 | 2.034 | 8.0 | | |
| | Fe-Fe1 | 0.8 | 3.651 | 4.8 | | |
| | Fe-Fe2 | 2.4 | 2.925 | 4.8 | | |
| | Fe-Fe3 | 2.4 | 3.350 | 3.1 | | |
| SA Fe-D 1T $MoS_2$[d] | Fe-S | 4.0 | 2.356 | 4.0 | -3.7 | 1.5 |

[a]N, coordination number; R, distance between absorber and backscatter atoms; $\sigma^2$, Debye-Waller factor to account for both thermal and structural disorders; $\Delta E_0$, inner potential correction; R factor (%) indicates the goodness of the fit. Error bounds (accuracies) that characterize the structural parameters obtained by EXAFS spectroscopy were estimated as N ± 20%; R ± 1%; $\sigma^2$ ± 20%; $\Delta E_0$ ± 20%. $S_0^2$ was fixed to 0.792 as determined from Co foil fitting. [b]Fitting range: 3.0 ⩽ k ($Å^{-1}$) ⩽ 10.0 and 1.25 ⩽ R (Å) ⩽ 3.0. [c]Fitting range: 3.0 ⩽ k ($Å^{-1}$) ⩽ 11.0 and 1.0 ⩽ R (Å) ⩽ 3.5. [d]Fitting range: 3.0 ⩽ k ($Å^{-1}$) ⩽ 10.0 and 1.15 ⩽ R (Å) ⩽ 2.7.

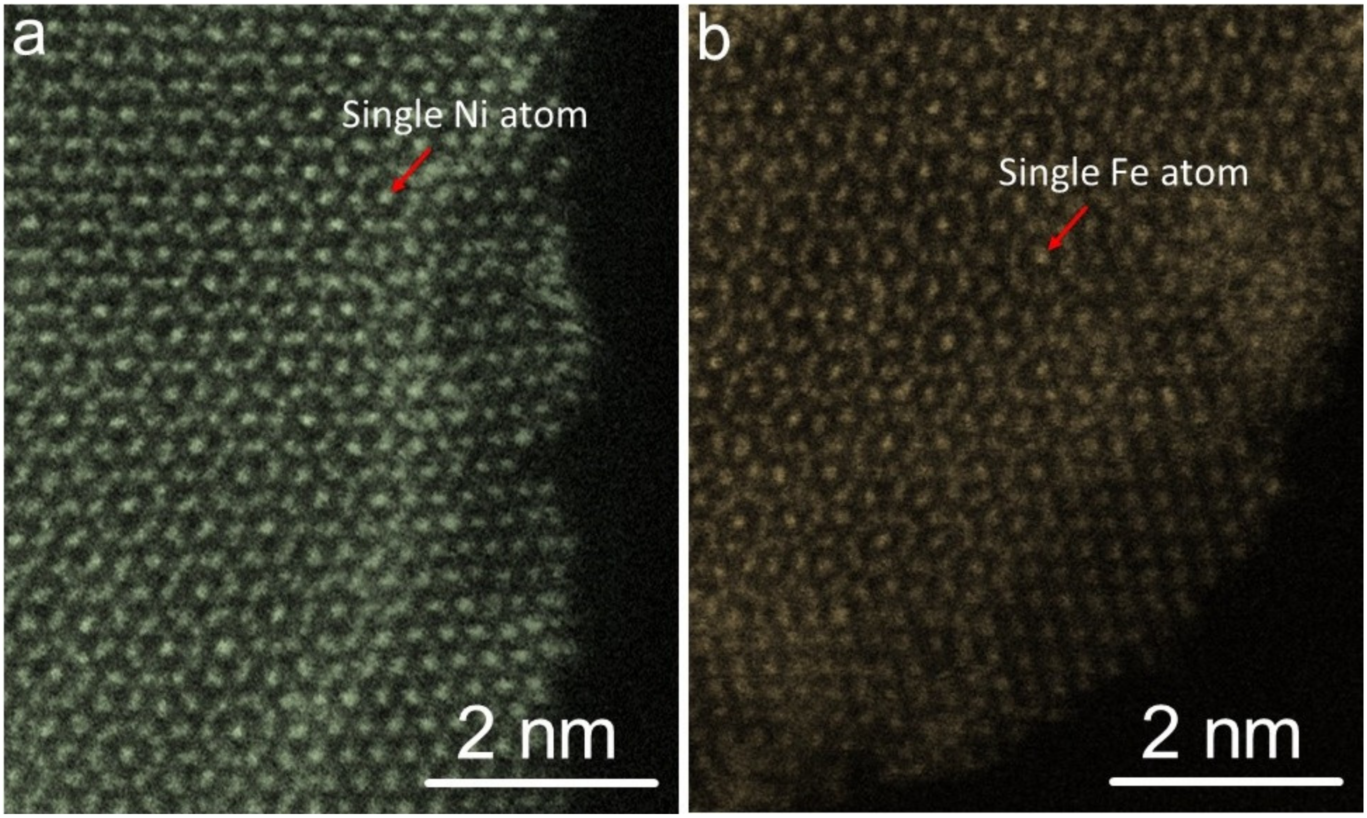


**Supplementary Figure 25.** HAADF-STEM images of **a,** SA Ni-D 1T $MoS_2$ and **b,** SA Fe-D 1T $MoS_2$.

## Supplementary Note 12

The charge density difference of Co atom on the surface of distorted 1T $MoS_2$ was calculated by:

$$\Delta\rho(r) = \rho_{Co\,/\,MoS_2}(r) - \rho_{MoS_2}(r) - \rho_{Co}(r)$$

where $\rho_{Co\,/\,MoS_2}(r)$ and $\rho_{MoS_2}(r)$ are the charge densities of the distorted 1T $MoS_2$ with and without Co adsorption, respectively. $\rho_{Co}(r)$ is the charge density of an isolated Co atom at the same position of the supercell. It reveals that the electron density surrounding Co atom increases as shown in Supplementary Figure 26. There is a charge accumulation between the adhering Co and S atoms, indicating the characteristics of covalent bonding at the Co adsorption site.[10]

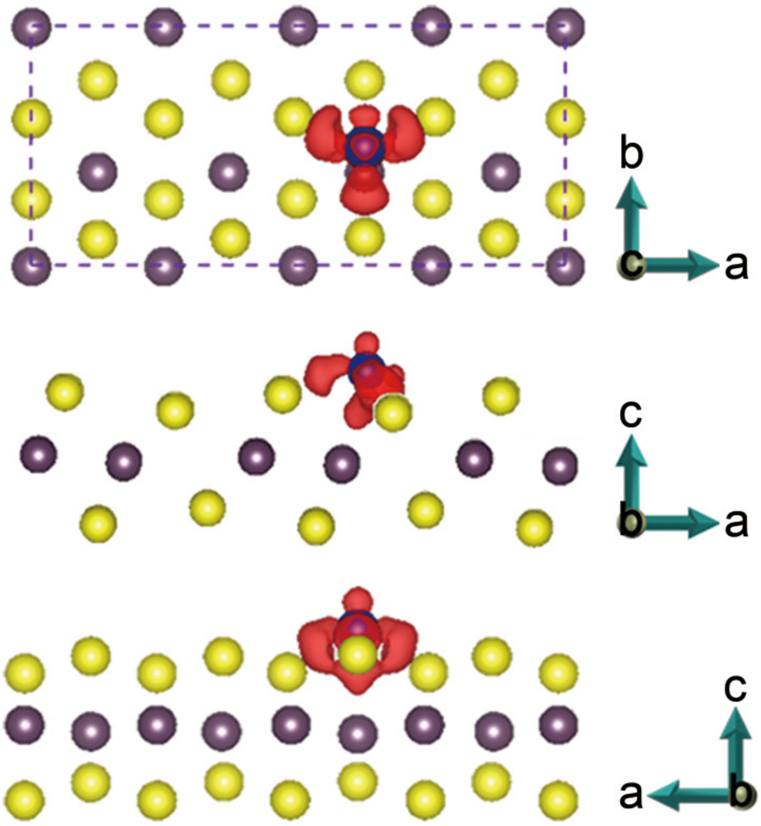


**Supplementary Figure 26.** The different views of charge density difference of Co atom on the surface of distorted 1T $MoS_2$.

## Supplementary Note 13

We have characterized the cross-section structure and interactions for the side view of the heterostructures. Supplementary Figure 27 show the TEM and HRTEM image of the Co NDs-$MoS_2$. The crystal alignment between Co nanodisks and $MoS_2$ nanosheets is that the contact layer of Co was covalently bonded with the S atom on the surface of $MoS_2$, also the contact layer of $MoS_2$ nearest to Co nanodisks shows a phase transformation from 2H to distorted 1T phase. We can also observe from the cross-section views of Co nanodisks-$MoS_2$ that there is a well-defined covalently bounded interface between the counterparts. As can be seen from the HRTEM in Supplementary Figure 27a, the Co nanodisk was loaded on the right top site of $MoS_2$ and close integration with it. In addition, the HRTEM image in Supplementary Figure 27b, there is a clearly well-defined covalently bounded interface between the Co atoms on the bottom layer of Co nanodisks and the S atoms on the top layer of $MoS_2$. The alignments and the interface between Co and $MoS_2$ are shown as the atomic model in Supplementary Figure 27c.

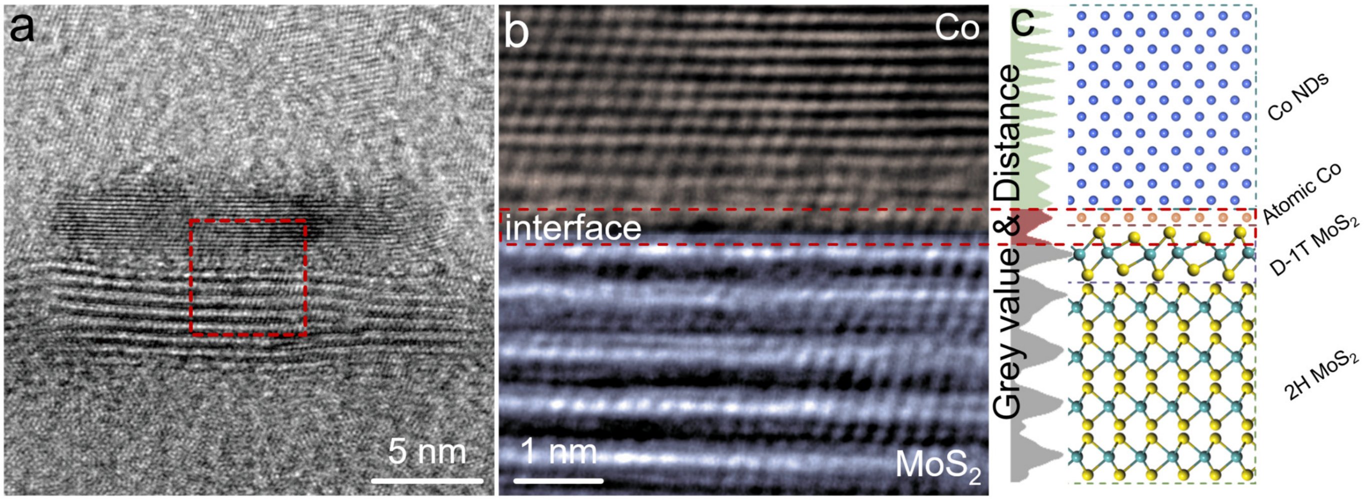


**Supplementary Figure 27.** Cross-section views of Co nanodisks-$MoS_2$ heterostructure show interface between Co nanodisks and $MoS_2$ nanosheets. **a,** HRTEM image and **b,** HRTEM image and **c,** relation between the grey value and distance and the atomic schematic for the interface.

## Supplementary Note 14

Supplementary Figure 28 shows that small new peaks ranging from 100 to 350 $cm^{-1}$ start to emerge from 6 h sonication.[11] After 10 h sonication, the peaks between 100 and 350 $cm^{-1}$ become more prominent, indicating the phase transformation of $MoS_2$ from pristine 2H to D-1T.[12]

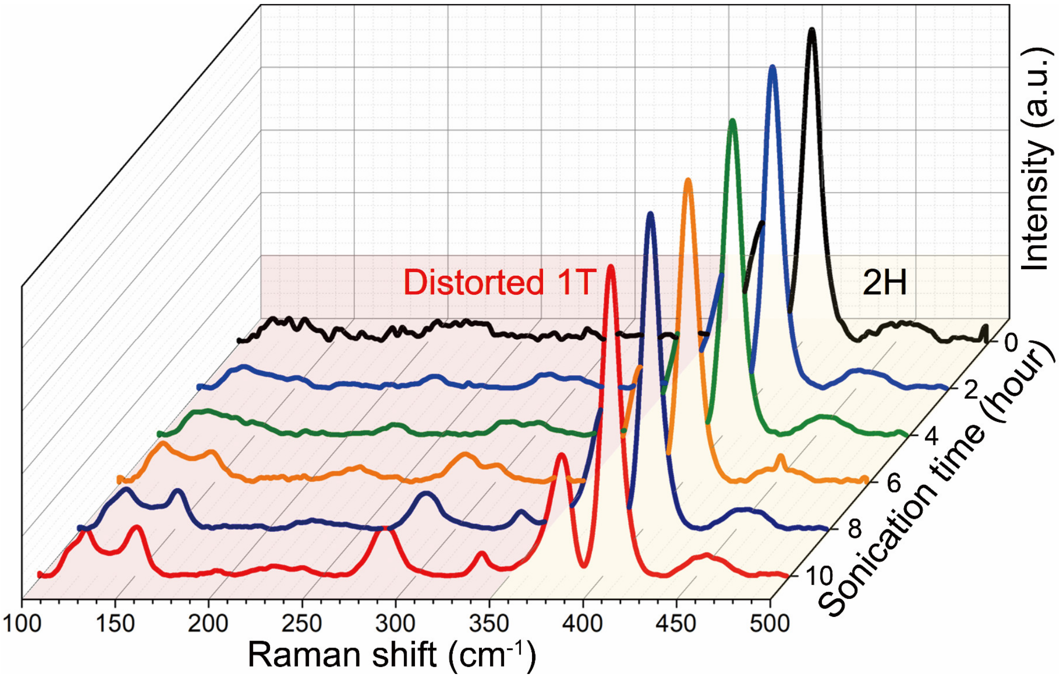


**Supplementary Figure 28.** Raman spectroscopy evolution when Co NDs and $MoS_2$ nanosheet were mixed together and sonicating for 0, 2, 4, 6, 8, and 10 h.

## Supplementary Note 15

Compared with the pristine $MoS_2$, the SA Co-D 1T $MoS_2$ showed two peaks at 161.6 and 162.7 eV for the S 2p, indicating the formation of metallic distorted 1T phase.[13]

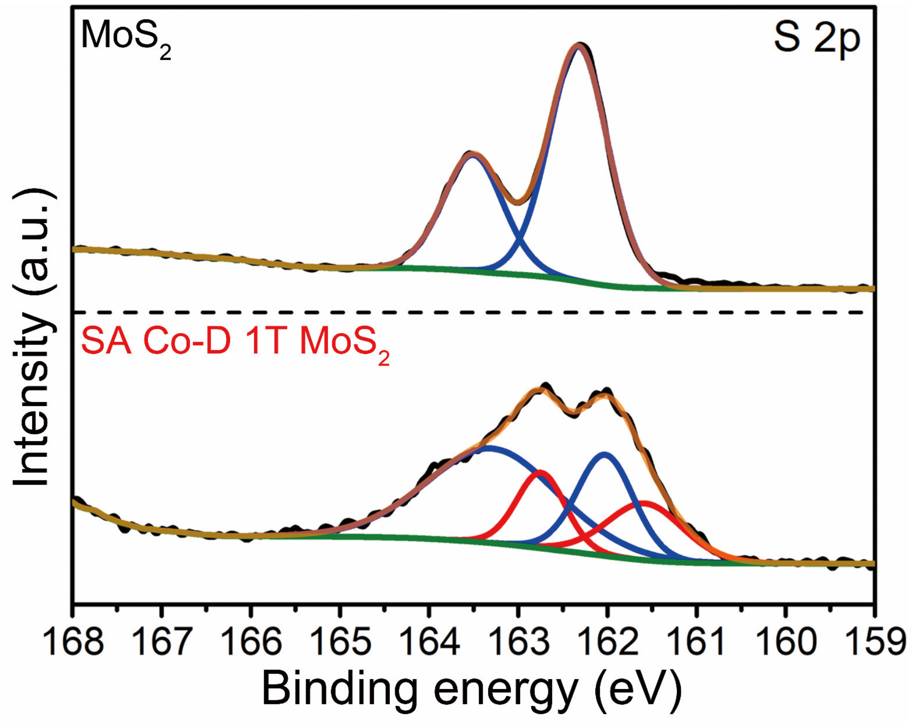


**Supplementary Figure 29.** S 2p XPS spectra show the phase transformation induced surface binding state variation after the SA Co formation.

## Supplementary Note 16

The results in Supplementary Figure 30 show that Co-S bond between Co NDs and $MoS_2$ was formed at 1 h sonication. The Co-S bond was getting dominant during the formation process of SA Co-D 1T $MoS_2$.[14,15]

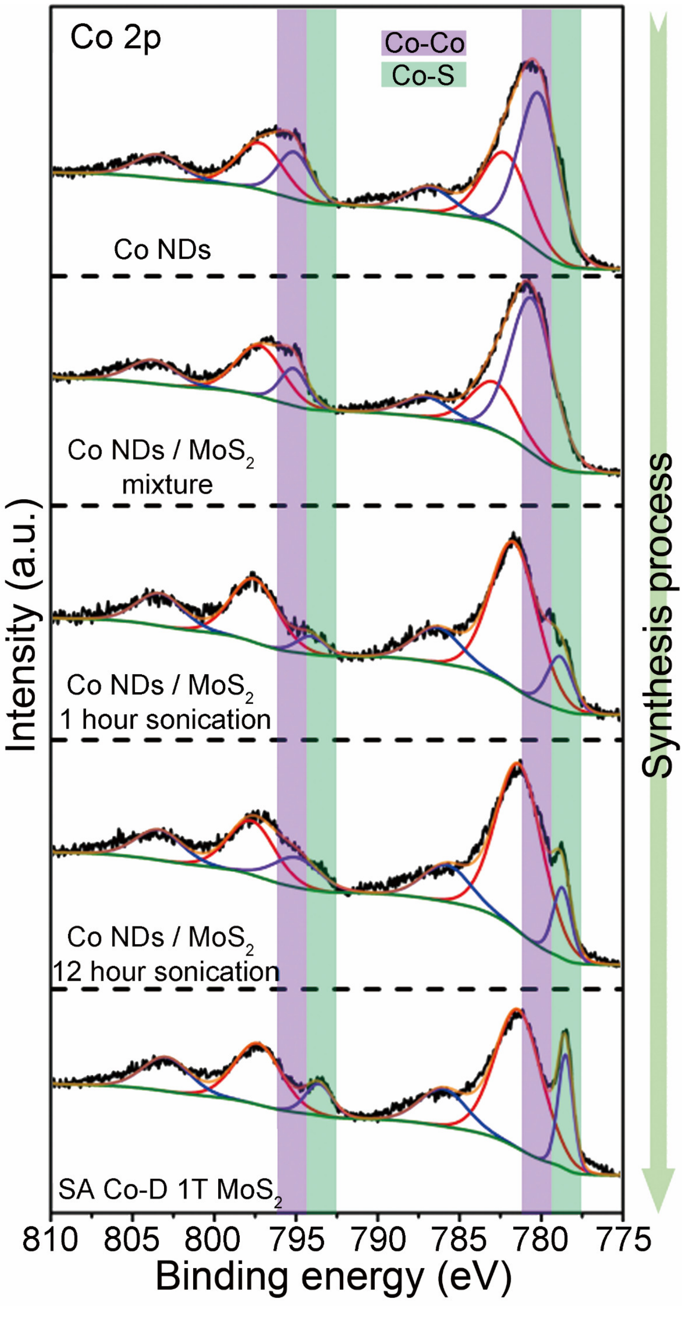


**Supplementary Figure 30.** Co 2p XPS spectra show the surface binding state variation of Co during the synthesis process.

## Supplementary Note 17

In the Supplementary Figure 31, the magnitude of strain was calculated as $\varepsilon = (a_{Co} - a_{Mo}) / a_{Mo}$, where $a_{Co}$ and $a_{Mo}$ are the distance between Co and Co atoms in Co NDs and the distance between Mo and Mo atoms in 2H $MoS_2$, respectively.[16,17] The lowest lattice strain between Co and $MoS_2$ can be estimated as 9.5%, which is much larger than 3.7% (The smallest strain need be triggered the phase transformation of $MoS_2$ from pristine 2H to 1T).

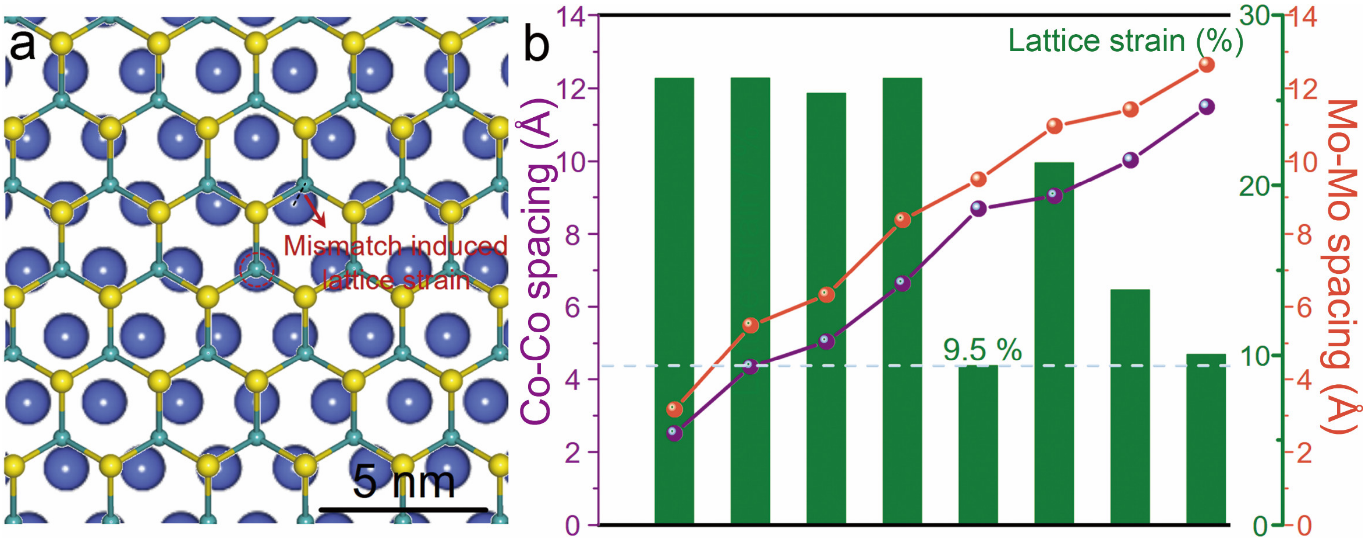


**Supplementary Figure 31.** Atomic structure and the theoretical lattice strain between Co and 2H $MoS_2$. **a,** Atomic structure illustration of Co and 2H $MoS_2$ and **b,** Co-Co and Mo-Mo spacings and their strain.

## Supplementary Note 18

In order to explore the hydrogen adsorption on single Co atom decorated 1T-$MoS_2$ substrate, we have studied the electronic and geometric structures of single Co atom decorated 1T-$MoS_2$ substrate. After geometry optimizations, the results show that the Co adatom can induce the coordination reconstruction of 1T-$MoS_2$ substrate, resulting in the charge-density wave (CDW) structures in Supplementary Figure 32. For 2×2 and 6×6 cases, we observed the formation of isolated Mo trimers in 1T-$MoS_2$ substrate, exhibiting orthorhombic structure marked by the color model. For 5×5 case, the Mo trimers coexist with some chains. But, around the Co adatom, the local configuration is essentially similar with 2×2 and 6×6 cases. For 3×3 case, the CDW structure presents the ribbon-like configuration. For 4×4 case, we observed the zigzag chain-like CDW structure. Above all, Co adsorbed 1T-$MoS_2$ substrates exhibiting the different stable CDW structures as derived from different Co adatom coverage.

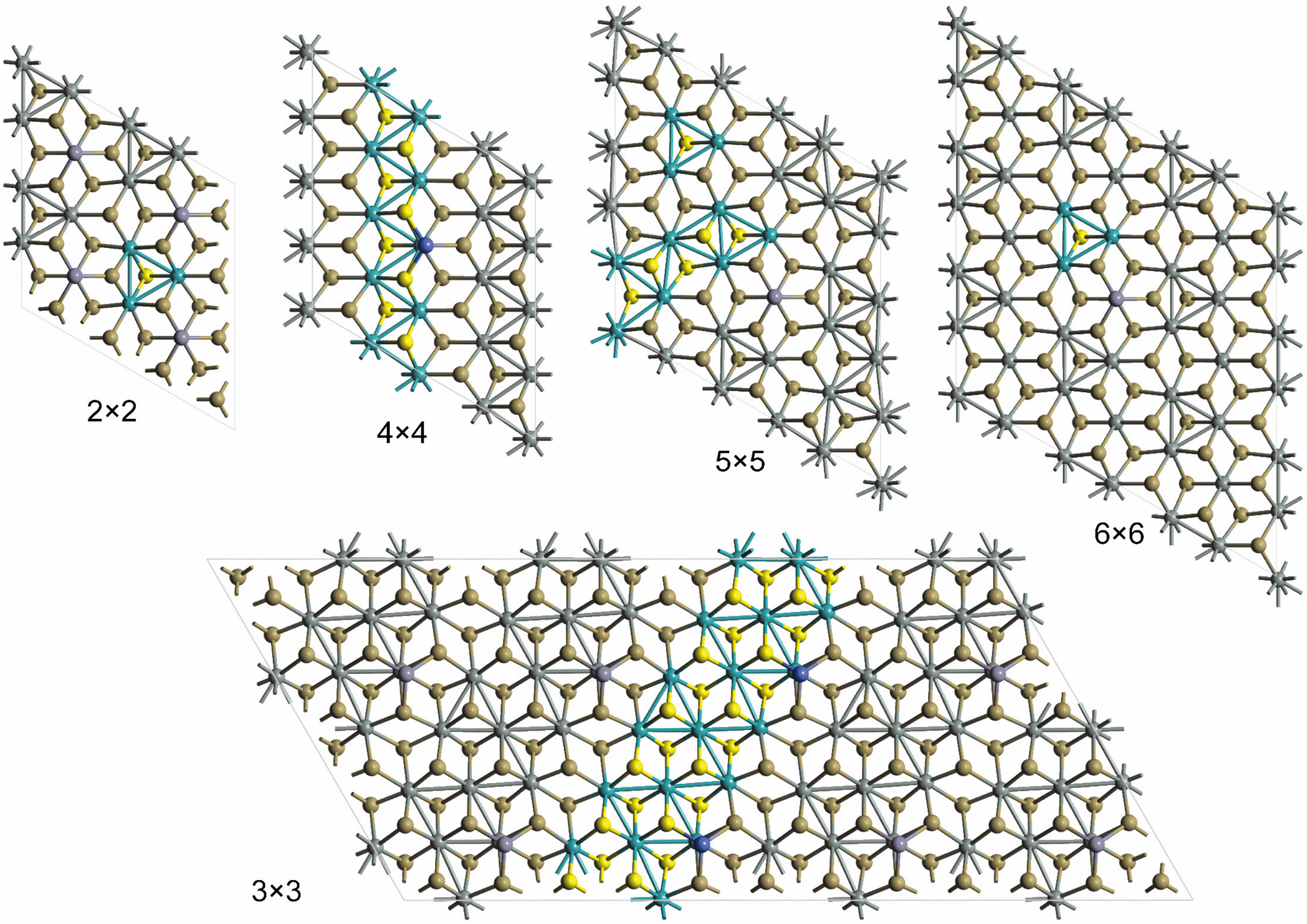


**Supplementary Figure 32.** The top views of CDW structures of $MoS_2$ with different Co coverages.

## Supplementary Note 19

The best HER activity of the catalyst was obtained by adding 800 μL of Co NDs into 1 mL $MoS_2$ that resulted in a 3.54% loading amount of Co in SA Co-D 1T $MoS_2$ based on the analysis of ICP.

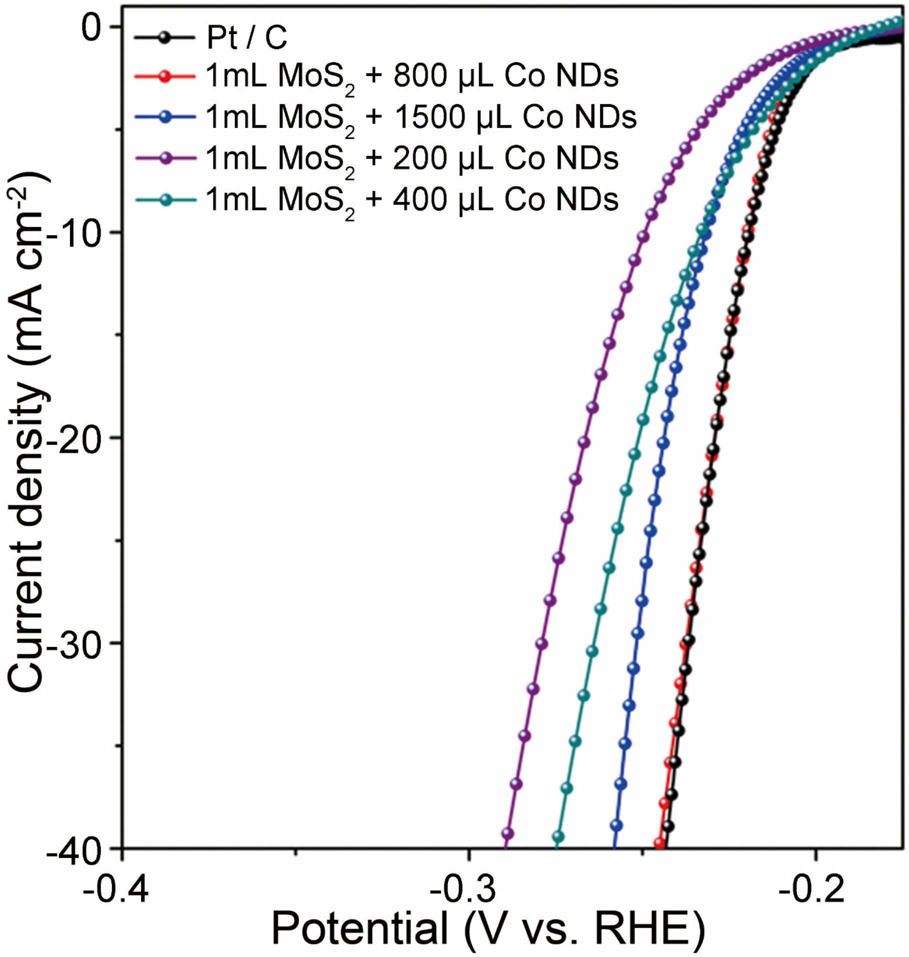


**Supplementary Figure 33.** HER polarization curves of SA Co-D 1T $MoS_2$ with different loading amount of Co nanodisks in Ar-saturated 0.5 M $H_2SO_4$.

**Supplementary Table 5.** ICP-MS data for the Co NDs on $MoS_2$ and SA Co-D 1T $MoS_2$.

| Sample | Co content / μg | Mo content / μg | Co mass percentage / % |
|---|---|---|---|
| Co NDs on $MoS_2$ | 226.93 | 350.14 | 20.71 |
| SA Co-D 1T $MoS_2$ | 21.72 | 355.12 | 3.54 |

**Supplementary Table 6.** Comparisons of the HER activity of SA Co-D 1T $MoS_2$ with other recently reported catalysts.

| Catalyst | $\eta$ at j = 10 mA $cm^{-2}$/ mV | Tafel slope / mV $dec^{-1}$ | Ref. |
|---|---|---|---|
| Sa Co-D 1T $MoS_2$ | **42** | **32** | **This work** |
| Pt/C | **30** | **31** | **This work** |
| Ni/GD | 86 | 45.8 | 18 |
| Fe/GD | 66 | 37.8 | 18 |
| Rh/$MoS_2$ | 50 | 24 | 19 |
| Ni-Graphene | 160 | 45 | 20 |
| NiO@1T $MoS_2$ | 46 | 52 | 21 |
| Li-PPS NDs | 110 | 29 | 22 |
| $MoSe_2$-NiSe | 210 | 56 | 23 |
| α-$MoB_2$ | 120 | 74.2 | 24 |
| IrCo@NC-500 | 24 | 23 | 25 |
| IrCo@NC-800 | 52 | 74 | 25 |
| Co-$Co_2P$@NPC/rGO | 136 | 50.64 | 26 |
| 17.7 wt. % Ir/SiNW | 22 | 20 | 27 |
| IrHNC | 4.5 | Not mentioned | 28 |
| Pt SA/m-$WO_{3-x}$ | 50 | 45 | 29 |
| RhPx@NPC | 22 | 32 | 30 |
| CoP/$Ni_5P_4$/CoP | 33 | 43 | 31 |

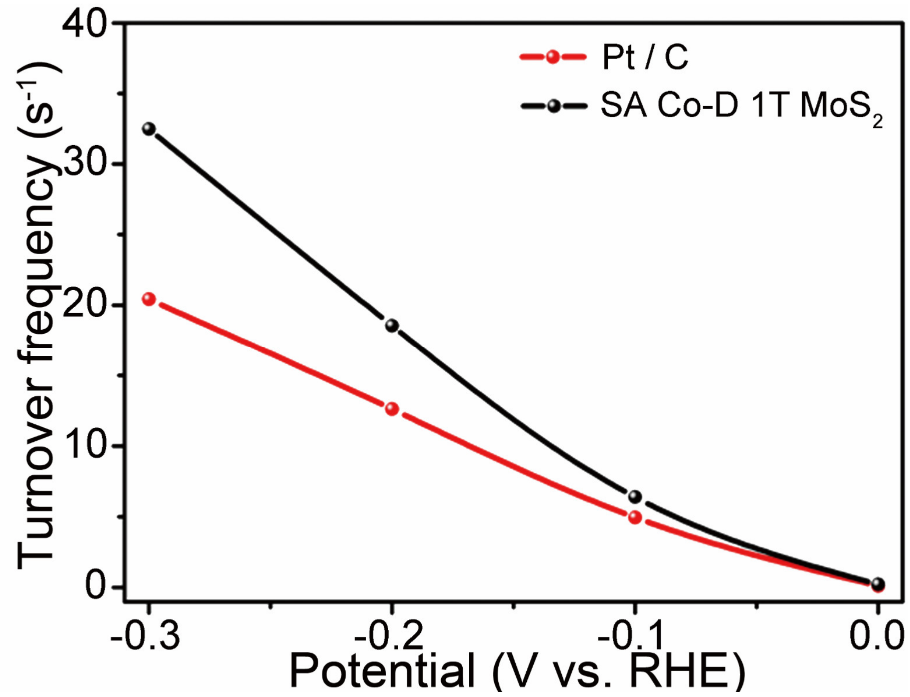


**Supplementary Figure 34.** The TOF plots of the SA Co-D 1T $MoS_2$ and commercial Pt/C catalysts.

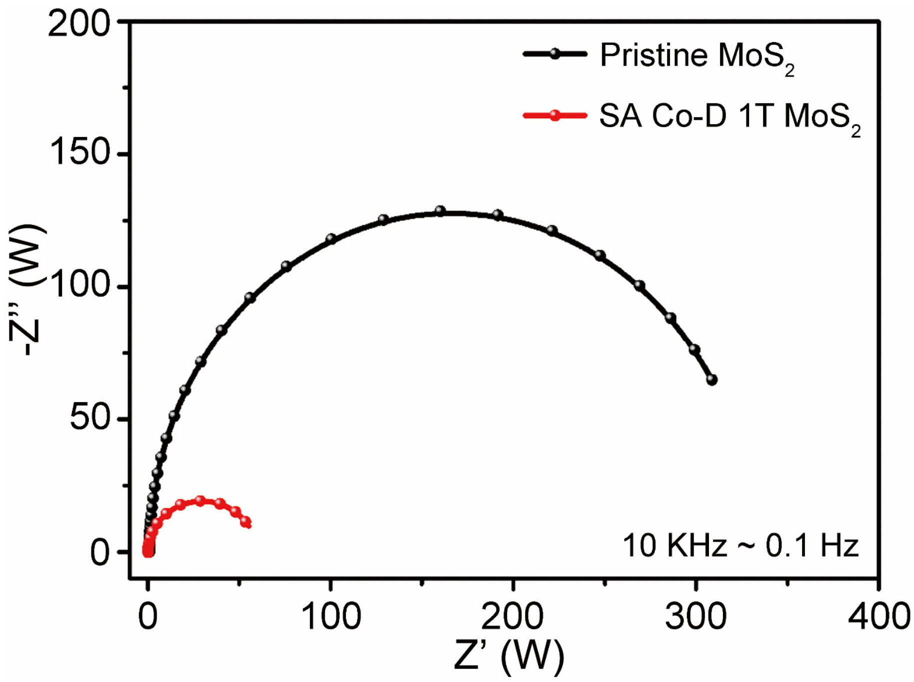


**Supplementary Figure 35.** Nyquist plots of EIS responses for the catalysts applied in 0.5 M $H_2SO_4$ solution at -0.2 V.

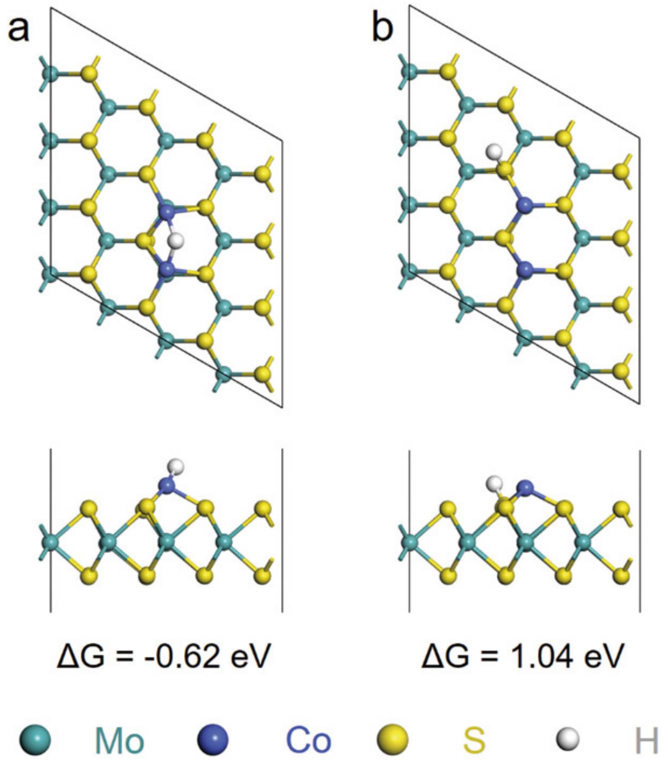


**Supplementary Figure 36.** The H* adsorption model on different adsorption sites with two adjacent Co atoms (dimer) on 2H $MoS_2$. **a,** Middle site of two Co atoms, **b,** the nearest S atom. Bottom is the corresponding energy of **a** and **b**, respectively.

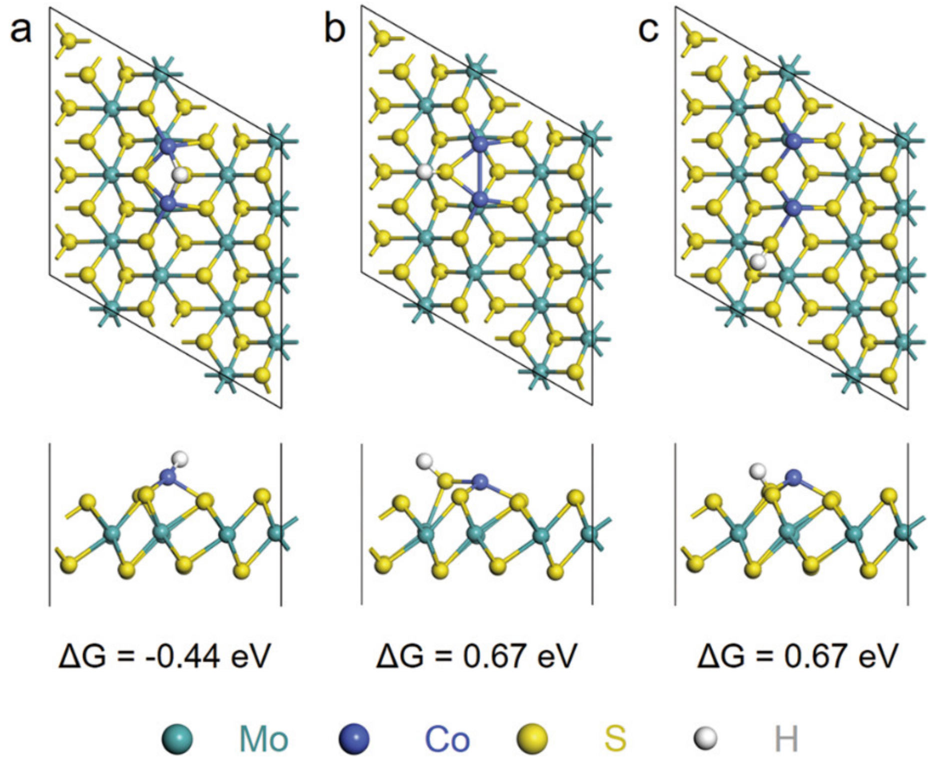


**Supplementary Figure 37.** The H* adsorption model on different adsorption sites with two adjacent Co atoms (dimer) on D-1T $MoS_2$. **a,** H was adsorbed on the middle site of two Co atoms, **b** and **c**, H was adsorbed on the nearest S atom. Bottom is the corresponding energy of **a, b** and **c**, respectively.

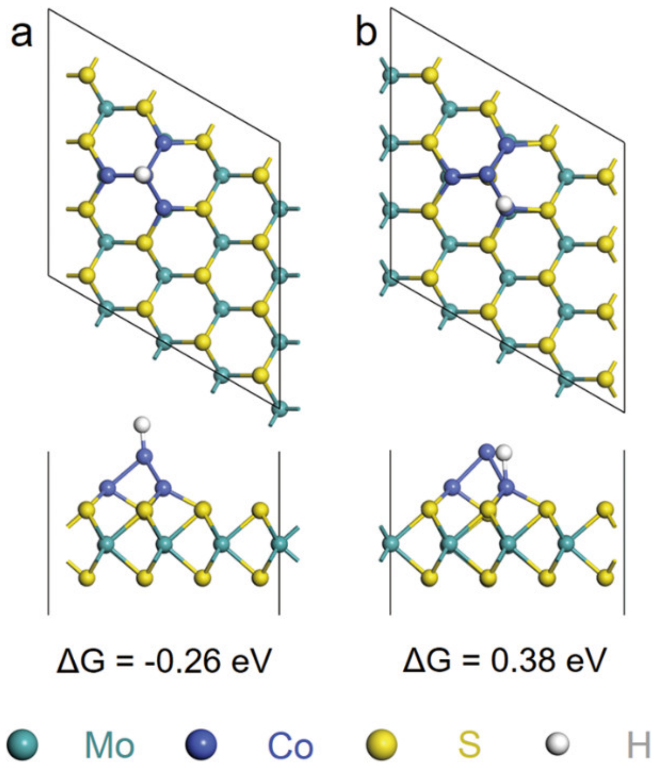


**Supplementary Figure 38.** The H* adsorption on different adsorption sites with four Co atoms clusters on 2H $MoS_2$. **a,** H was adsorbed on the middle site on the top of Co cluster, **b,** H was adsorbed on the bottomed Co atom. Bottom is the corresponding energy of **a** and **b**, respectively.

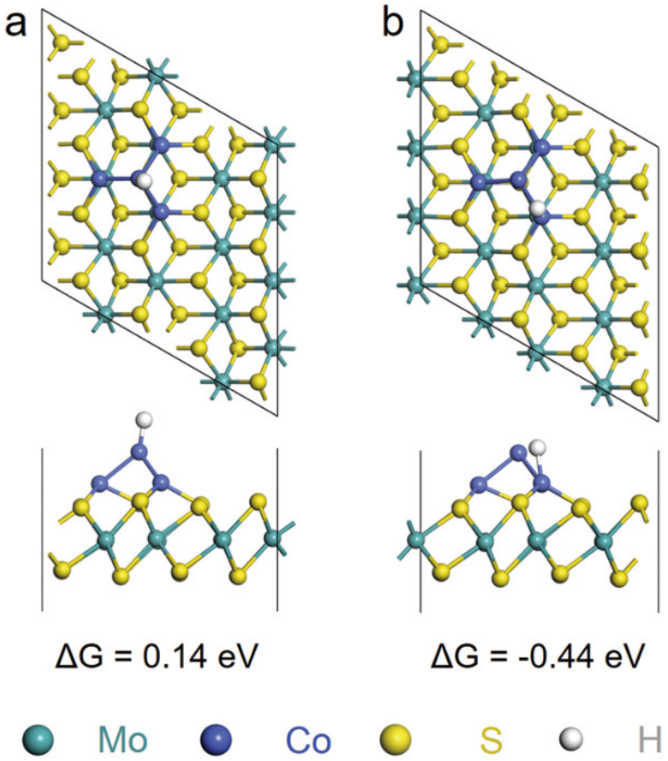


**Supplementary Figure 39.** The H* adsorption model on different adsorption sites with four Co atoms formed cluster on D-1T $MoS_2$. **a,** H was adsorbed on the middle site on the top of Co cluster, **b,** H was adsorbed on the bottomed Co atom. Bottom is the corresponding energy of **a** and **b**, respectively.

## Supplementary Note 20

Supplementary Figure 40 show that Co cluster/2H $MoS_2$ catalyst prepared by *in-situ* reducing low concentration Co salt precursor is neither able to induce the phase transition, nor to improve the hydrogen evolution reaction activity.

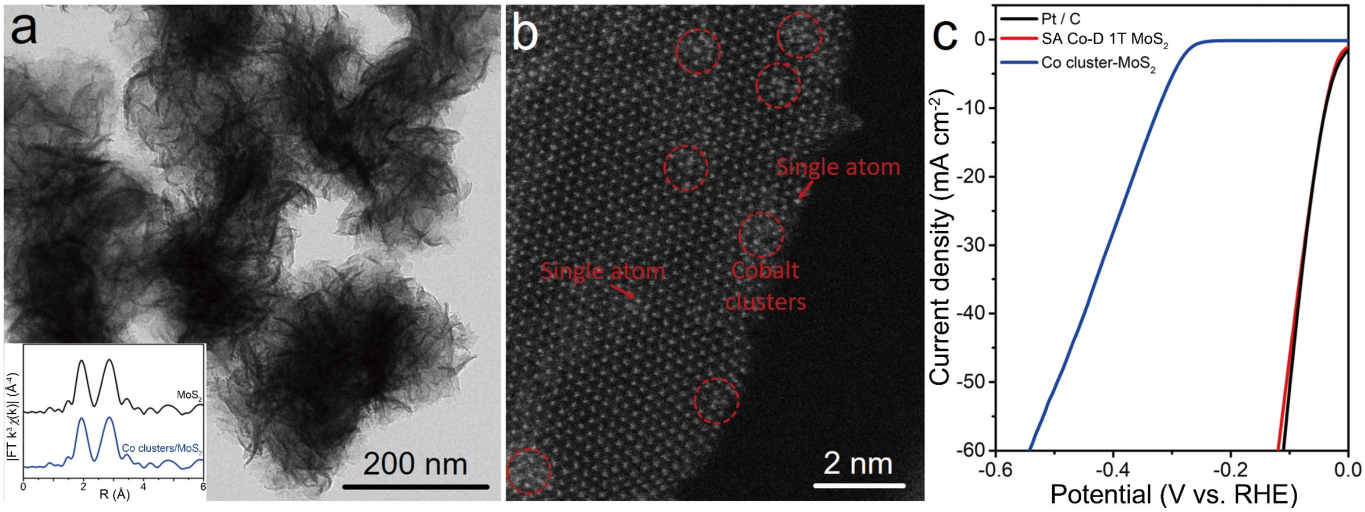


**Supplementary Figure 40.** The structure characterization and HER activity measurement of Co cluster (with some single atom) 2H $MoS_2$ catalyst. **a,** TEM image, *inset* shows the Mo K-edge EXAFS spectrum. **b,** HAADF-STEM image (cobalt clusters were observed as bright spots, mainly located at the edge sites of 2H phase $MoS_2$). **c,** HER LSV plot of the catalysts.

## Supplementary Note 21

The Co K-edge FT-EXAFS spectra of Co clusters/$MoS_2$ shows slight emergence of Co-S bond compared with Co NDs, but the main content of bonding is still Co-Co, assigned to the feature of Co clusters.[32]

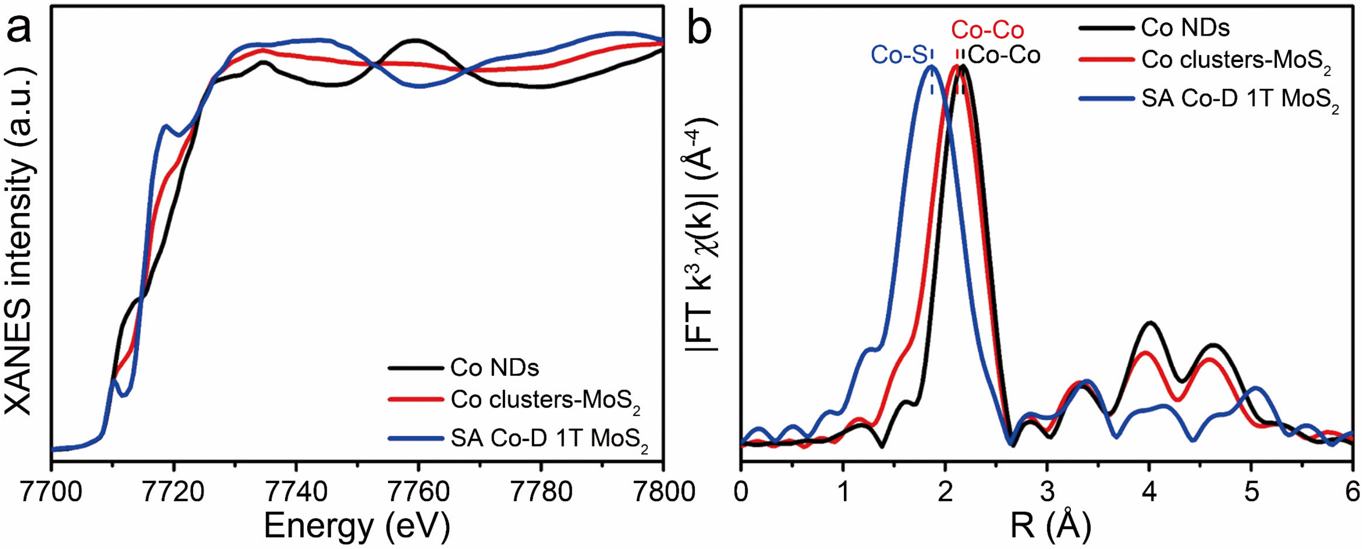


**Supplementary Figure 41.** Co K-edge **a,** XANES and **b,** FT-EXAFS spectra of SA Co-D 1T $MoS_2$, Co clusters/$MoS_2$ and Co NDs.

## Supplementary Note 22

We tried Co nanoparticles and Co ions for comparison. Two strategies were used for the decoration of Co nanoparticles: 1) Co nanoparticles were first prepared and then assembled on $MoS_2$ by sonication (Supplementary Figure 42b); 2) Co nanoparticles were *in-situ* grown on $MoS_2$ by reducing cobalt precursors (Supplementary Figure 42c). Co nanoparticles in both cases were located mainly on the edge and defects sites of $MoS_2$, which did not induce the phase transition (Supplementary Figure 42e). Same thing happened for Co ions (Supplementary Figure 42d-42e). Only Co nanodisks could be effectively assembled on the basal plane of $MoS_2$ and induce the phase transition (Supplementary Figure 42a). The above results show that other methods could not induce the phase transformation of $MoS_2$.

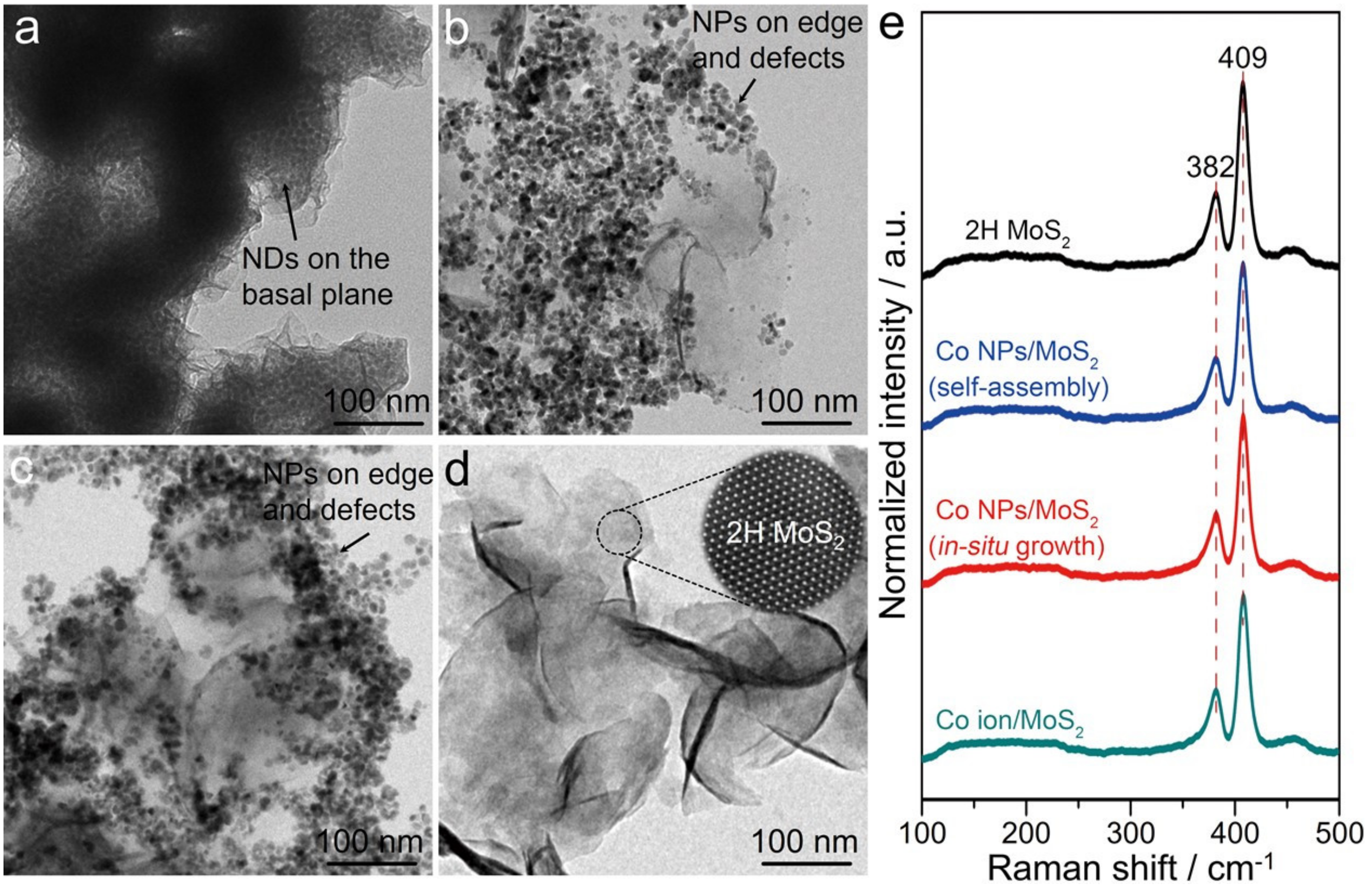


**Supplementary Figure 42.** Structural characterization of the Co-$MoS_2$ catalysts synthesized by different method and their corresponding Raman spectra. **a,** Co NDs/$MoS_2$. **b,** Co NPs/$MoS_2$ by self-assembly method. **c,** Co NPs/$MoS_2$ by *in-situ* growth method. **d,** Co ion/$MoS_2$ and **e,** Raman spectrum of the Co/$MoS_2$ catalysts synthesized by different methods.

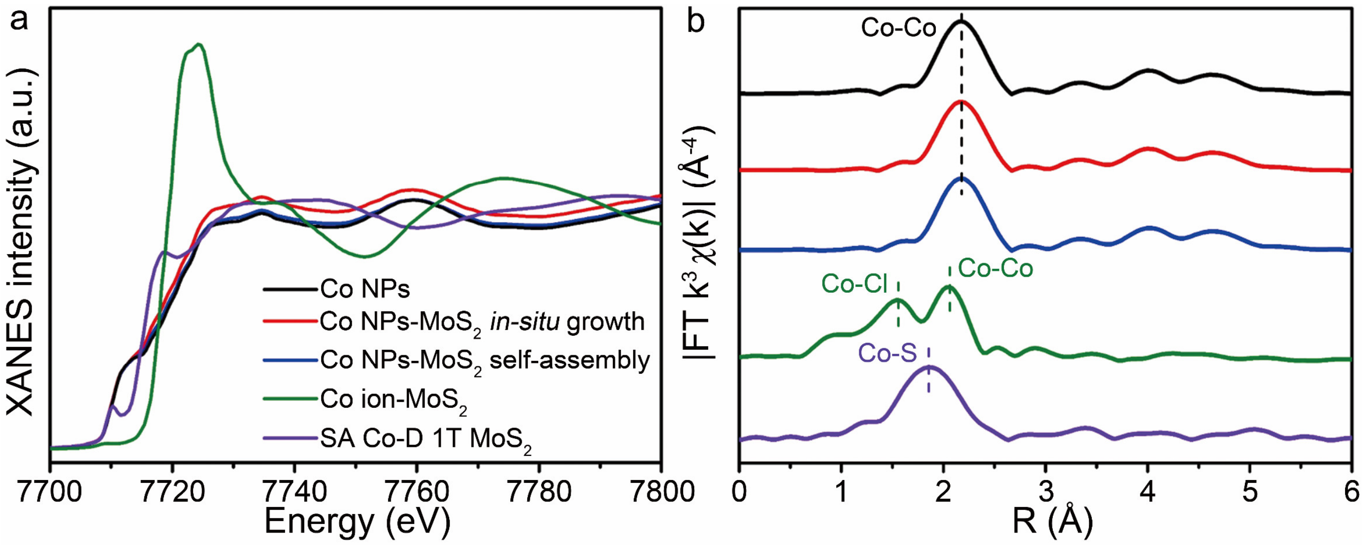


**Supplementary Figure 43.** Co K-edge **a,** XANES and **b,** FT-EXAFS spectra of the Co/$MoS_2$ catalysts prepared by different methods.

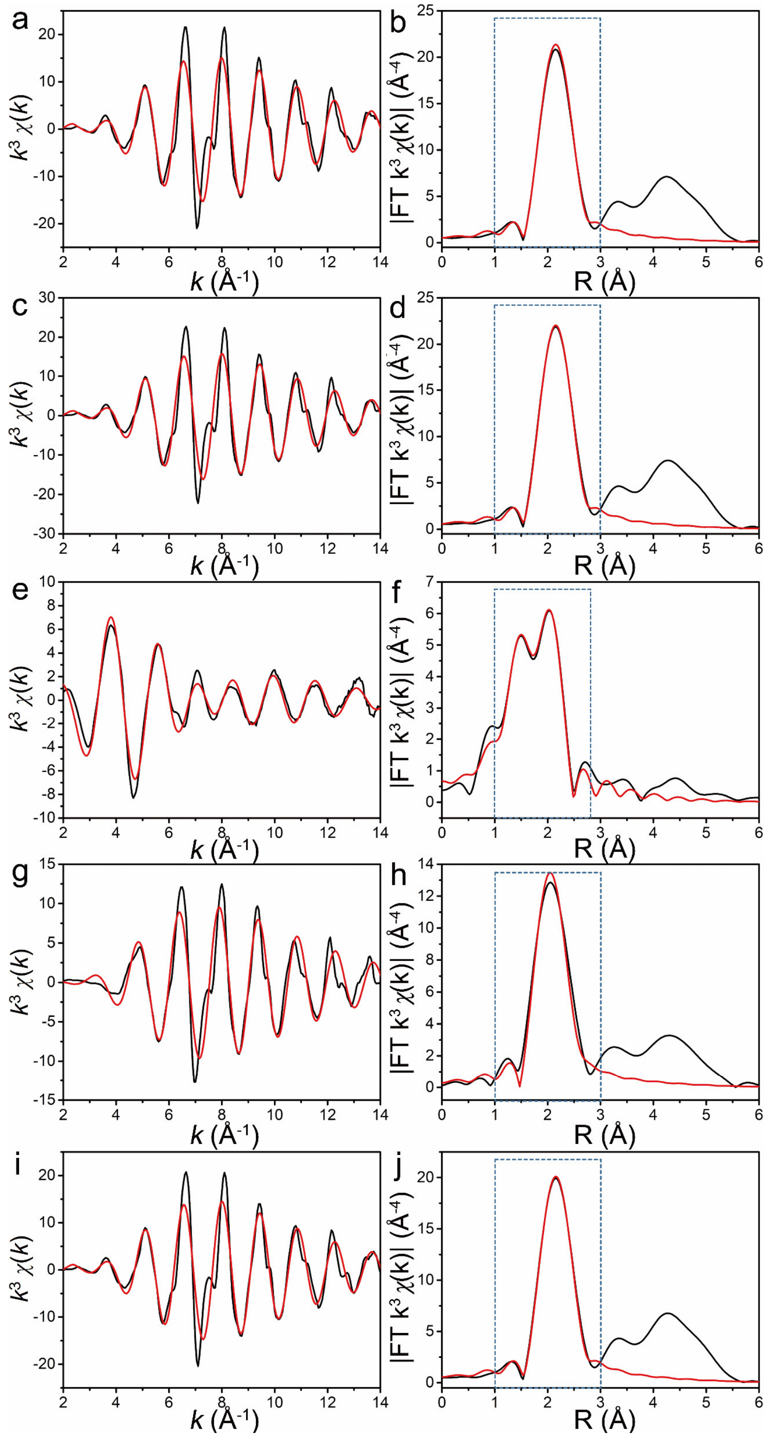


**Supplementary Figure 44.** Fourier-transformed magnitude of Co K-edge EXAFS spectra in *k* and R space for **a-b,** Co NPs/$MoS_2$ (self-assembly), **c-d,** Co NPs/$MoS_2$ (*in-situ* growth), **e-f,** Co ion/$MoS_2$, **g-h,** Co clusters/$MoS_2$ and **i-j,** Co NDs/$MoS_2$ heating. The measured and calculated spectra are well matched for all samples. The best-fit parameters are shown in Supplementary Table 7.

**Supplementary Table 7.** Co K-edge EXAFS curve Fitting Parameters obtained from Supplementary Figure 44 for Co/$MoS_2$ catalysts synthesized by different methods.[a]

| Sample | Path | CN | R(Å) | $\sigma^2$($\times10^{-3}$Å$^2$) | $\triangle E_0$(ev) | R,% |
|---|---|---|---|---|---|---|
| Co NPs/$MoS_2$ (self-assembly)[b] | Co-Co | 9.5 | 2.489 | 6.1 | 6.0 | 0.2 |
| Co NPs/$MoS_2$ (*in-situ* growth)[c] | Co-Co | 10.0 | 2.486 | 6.2 | 6.4 | 0.2 |
| Co ion/$MoS_2$[d] | Co-Co | 5.8 | 2.308 | 8.4 | -12.8 | 0.3 |
| | Co-Cl | 12.1 | 2.263 | 11.7 | | |
| Co clusters/$MoS_2$[e] | Co-Co | 6.2 | 2.458 | 5.9 | -6.4 | 1.4 |
| Co NDs/$MoS_2$ heating[f] | Co-Co | 9.0 | 2.487 | 6.0 | 6.6 | 0.2 |

[a]N, coordination number; R, distance between absorber and backscatter atoms; $\sigma^2$, Debye-Waller factor to account for both thermal and structural disorders; $\Delta E_0$, inner potential correction; R factor (%) indicates the goodness of the fit. Error bounds (accuracies) that characterize the structural parameters obtained by EXAFS spectroscopy were estimated as N ± 20%; R ± 1%; $\sigma^2$ ± 20%; $\Delta E_0$ ± 20%. $S_0^2$ was fixed to 0.707 as determined from Co foil fitting. [b]Fitting range: 3.0 ⩽ k (Å$^{-1}$) ⩽ 10.0 and 1.0 ⩽ R (Å) ⩽ 3.0. [c]Fitting range: 3.0 ⩽ k (Å$^{-1}$) ⩽ 10.0 and 1.0 ⩽ R (Å) ⩽ 3.0. [d]Fitting range: 3.0 ⩽ k (Å$^{-1}$) ⩽ 10.0 and 1.0 ⩽ R (Å) ⩽ 2.8. [e]Fitting range: 3.0 ⩽ k (Å$^{-1}$) ⩽ 10.0 and 1.0 ⩽ R (Å) ⩽ 3.0. [f]Fitting range: 3.0 ⩽ k (Å$^{-1}$) ⩽ 10.0 and 1.0 ⩽ R (Å) ⩽ 3.0.

## Supplementary Note 23

Electrochemical results in Supplementary Figure 46 show that Co/$MoS_2$ catalysts synthesized by other methods could not enhance the HER activity. The reason for the poor performances is explained by the fact that the direct epitaxial growth or assembly of small Co nanoparticles would occur on the uncoordinated S atoms of the boundary defect sites, which will definitely block the HER active sites.[33]

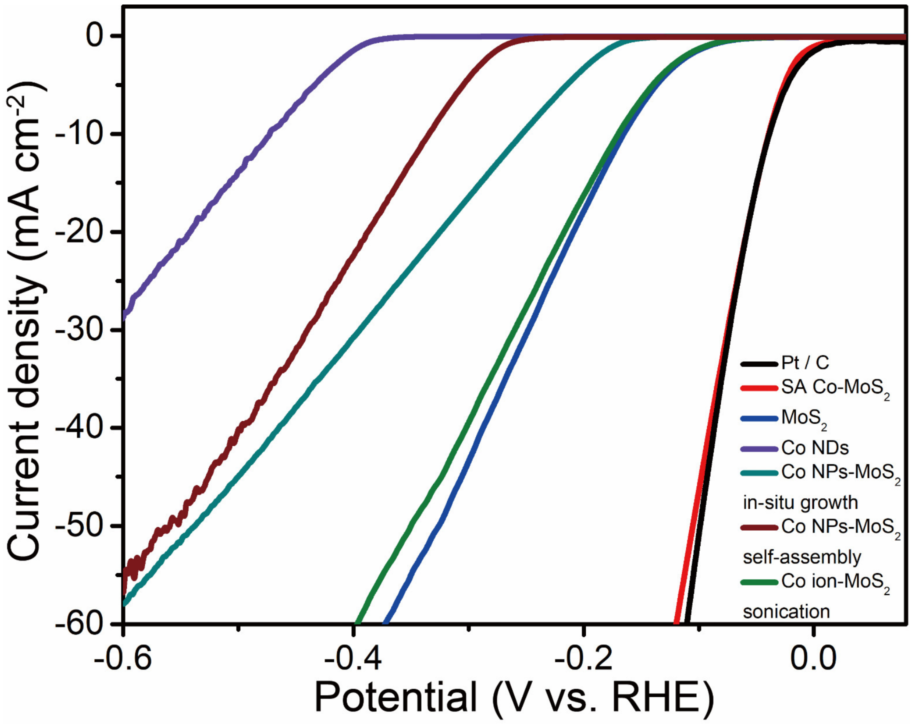


**Supplementary Figure 45.** HER polarization curves of the Co/$MoS_2$ catalysts synthesized by different methods.

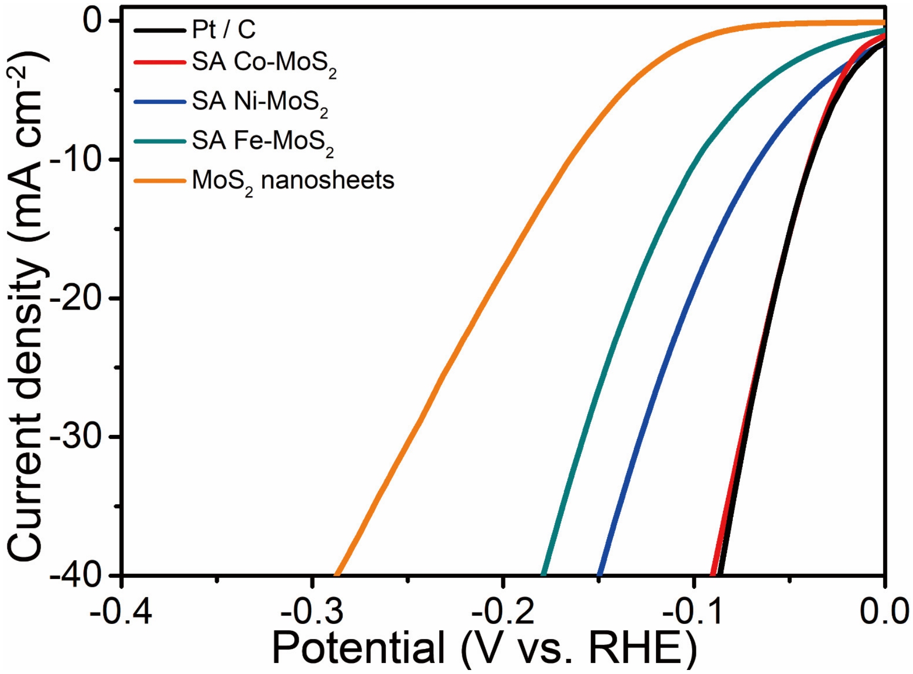


**Supplementary Figure 46.** Polarization curves of SA Ni-D 1T $MoS_2$, SA Fe-D 1T $MoS_2$ and other catalysts tested in Ar-saturated 0.5 M $H_2SO_4$.

## Supplementary Note 24

As shown in the LSV curve in Supplementary Figure 46, both Ni, Fe and Co single atoms on $MoS_2$ show improved HER activity than the pristine $MoS_2$. Among these catalysts, SA Co on D-1T $MoS_2$ shows the best performance. These experimental results are in good agreement with the DFT calculation results of the hydrogen adsorption free energy of single atom Ni, Fe and Co on 2H and D-1T $MoS_2$ as shown in Supplementary Table 8.

**Supplementary Table 8.** Comparison of the DFT hydrogen adsorption free energy of single metal atom loaded on 2H and D-1T $MoS_2$.

| ΔG / eV | Coverage | | | |
|---|---|---|---|---|
| | 2 × 2 | 3 × 3 | 4 × 4 | 5 × 5 |
| SA Co-2H $MoS_2$ | 0.11 | 0.07 | 0.11 | 0.24 |
| SA Ni-2H $MoS_2$ | 0.36 | 0.42 | 0.46 | 0.50 |
| SA Fe-2H $MoS_2$ | 0.41 | 0.52 | 0.06 | 0.10 |
| SA Co-D 1T $MoS_2$ | 0.24 | **0.03** | -0.18 | 0.32 |
| SA Ni-D 1T $MoS_2$ | 0.44 | 0.27 | 0.79 | 0.49 |
| SA Fe-D 1T $MoS_2$ | 0.12 | 0.06 | 0.13 | 0.20 |

## Supplementary Note 25

After accelerated degradation studies, no Co nanoparticles or clusters were observed and the Co elements are uniformly distributed. Both the Co single atom and the phase transition of $MoS_2$ can be observed clearly from the HAADF-STEM image, further confirm the high atomic structure and element stability of as prepared SA Co-D 1T $MoS_2$ catalyst.[34]

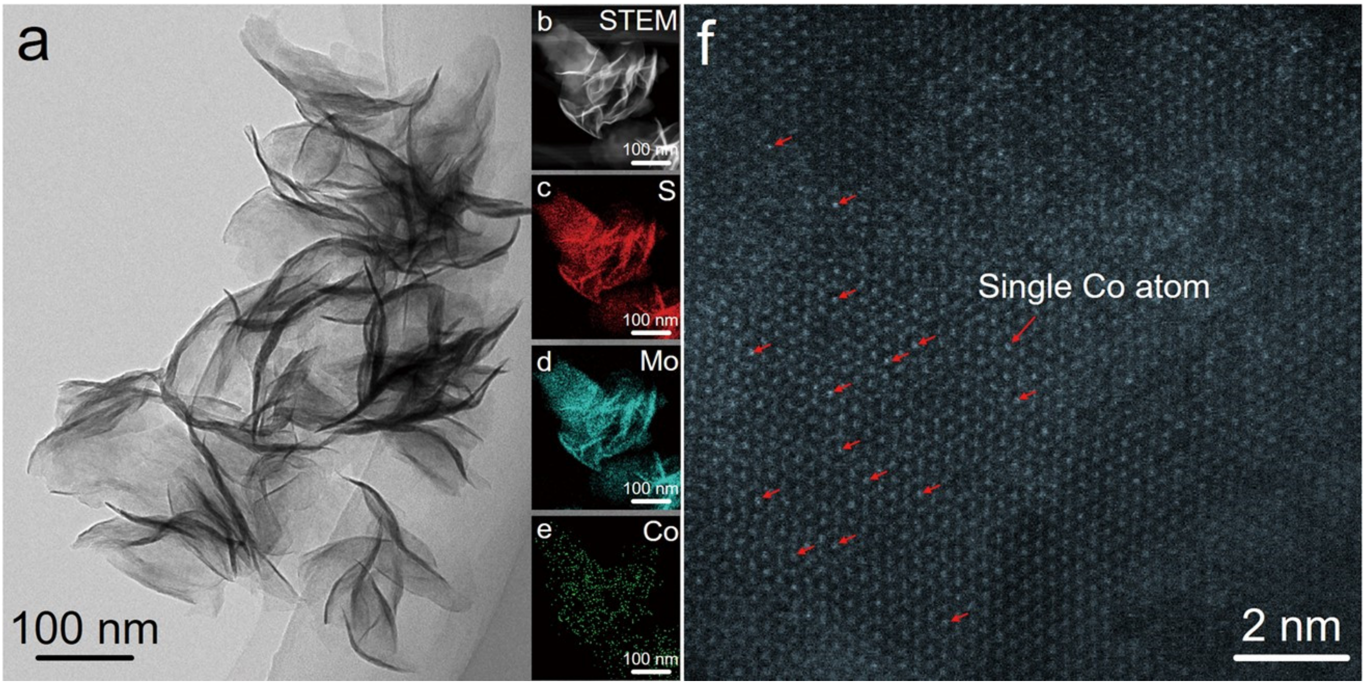


**Supplementary Figure 47.** TEM, EDX-mapping and HAADF-STEM images of the SA Co-D 1T $MoS_2$ catalyst after 10 h long-term test at the overpotential of 100 mV *vs.* RHE.

## Supplementary Note 26

There is no obvious change in XPS of both Mo 3d and Co 2p, indicating the high valence state and phase stability of as prepared SA Co-D 1T $MoS_2$ catalyst.[35]

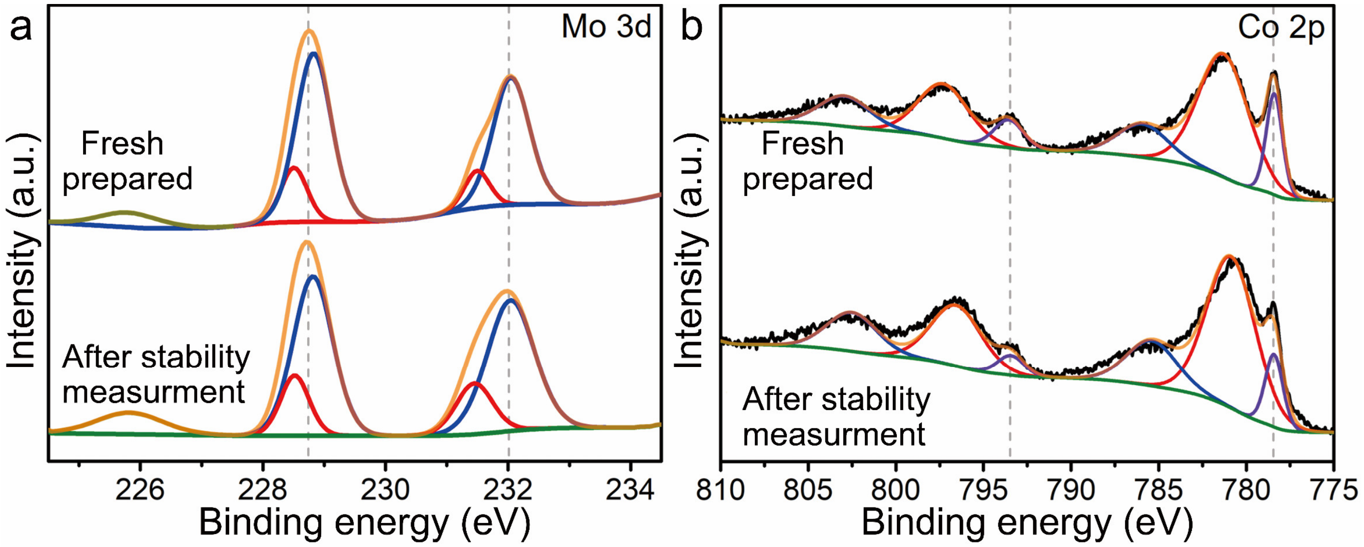


**Supplementary Figure 48.** XPS spectra of the SA Co-D 1T $MoS_2$ catalyst before and after 10 h electrochemical stability measurement at the overpotential of 100 mV *vs*. RHE. **a,** Mo 3d and **b,** Co 2p.

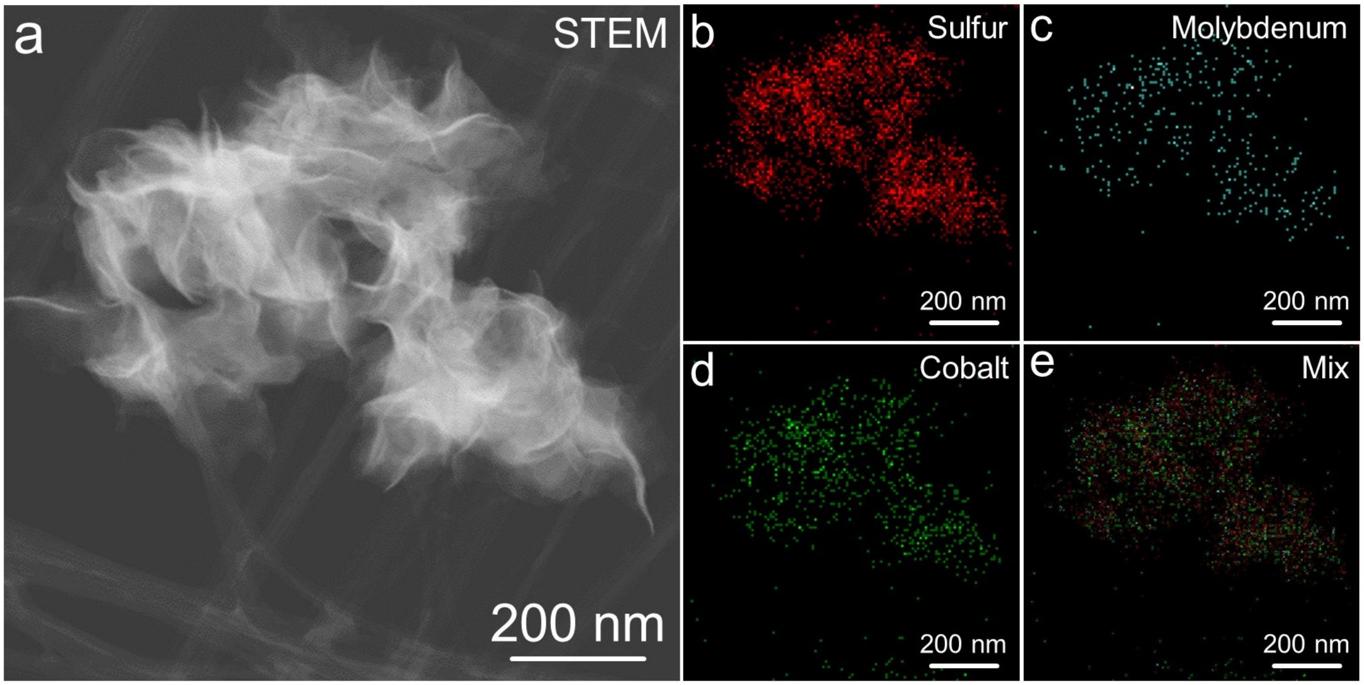


**Supplementary Figure 49. a,** STEM and **b-e,** EDX mapping images of the SA Co-D 1T $MoS_2$ catalyst after its exposed in air for 3 months.

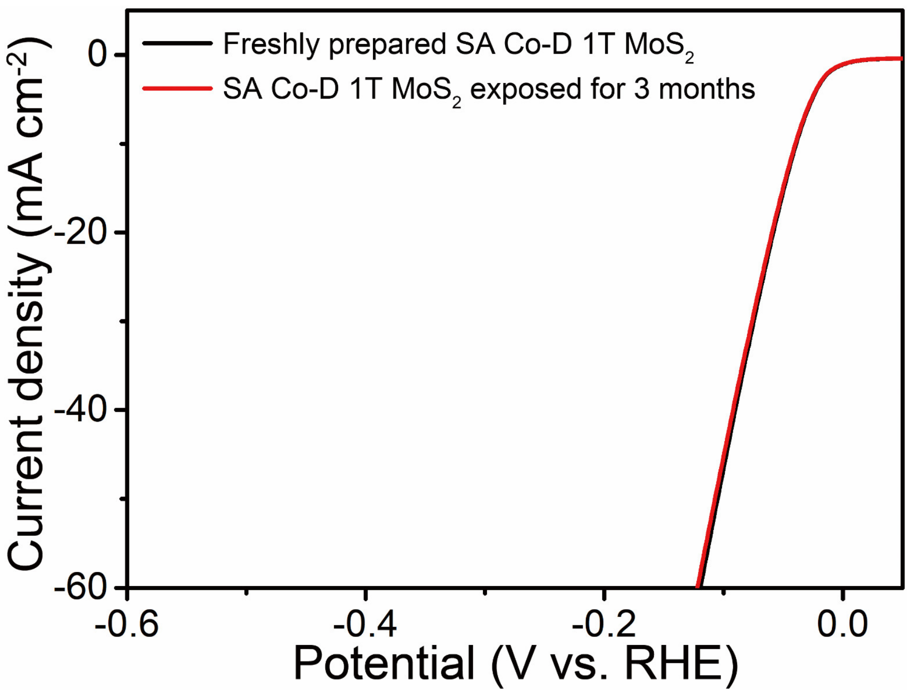


**Supplementary Figure 50.** Polarization curves of the SA Co-D 1T $MoS_2$ catalyst before and after its exposed in air for 3 months.

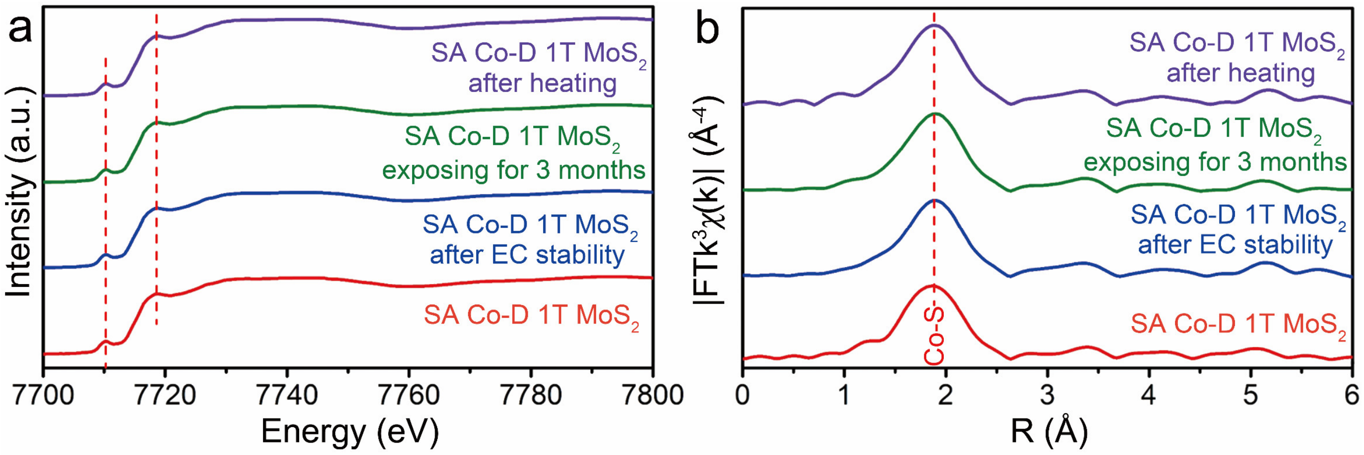


**Supplementary Figure 51.** SA Co-D 1T $MoS_2$ before and after HER reaction at the overpotential of 200 mV for 25 h, 3-month storage in an ambient atmosphere at room temperature and heating at 80 °C for 12 h. **a,** Co K edge XANES spectra and **b,** FT-EXAFS spectra.

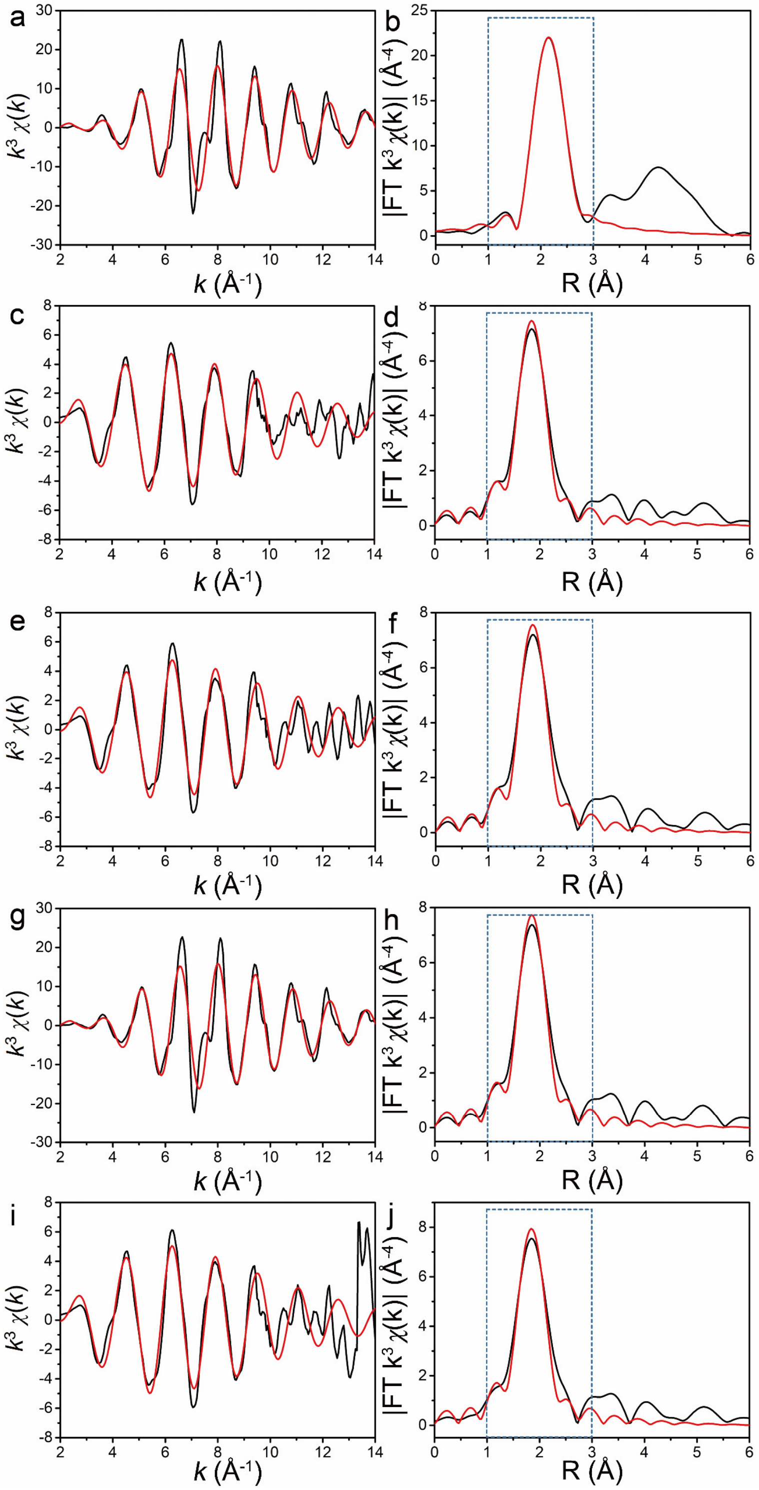


**Supplementary Figure 52.** Fourier-transformed magnitude of Co K-edge EXAFS spectra in k and R space for **a-b,** Co foil, **c-d,** SA Co-D 1T $MoS_2$, **e-f,** SA Co-D 1T $MoS_2$ after the electrochemical stability, **g-h,** SA Co-D 1T $MoS_2$ after exposing for 3 months and **i-j,** SA Co-D 1T $MoS_2$ after heating at 80 °C for 12 h. The measured and calculated spectra are well matched for all samples. The best-fit parameters are shown in Supplementary Table 9.

## Supplementary Note 27

The Supplementary Figure 51-52 and Supplementary Table 9 show that no obvious structural changes were observed from HAADF-STEM, XPS, XANES and EXAFS spectra of the used and the fresh catalysts. These observations suggest that there was no significant Co aggregation and no apparent structural changes for the coordination environment of cobalt in these samples, further confirming the excellent coordination environment stability of as prepared SA Co-D 1T $MoS_2$ catalyst.[36,37]

**Supplementary Table 9.** Co K-edge EXAFS curve Fitting Parameters obtained from Supplementary Figure 52 for SA Co-D 1T $MoS_2$ catalysts after the stability measurement.

| Sample | Path | CN | R(Å) | $\sigma^2$(×$10^{-3}$Å$^2$) | $\Delta E_0$ (eV) | R,% |
|---|---|---|---|---|---|---|
| Co foil[b] | Co-S | 12.0 | 2.488 | 6.1 | 6.4 | 0.1 |
| SA-Co[c] | Co-S | 3.8 | 2.384 | 6.3 | -0.5 | 1.4 |
| SA-Co heating[d] | Co-S | 4.0 | 2.384 | 6.2 | -0.3 | 2.3 |
| SA-Co EC stability[e] | Co-S | 3.6 | 2.384 | 5.7 | 0.1 | 2.4 |
| SA-Co exposing for 3 months[f] | Co-S | 3.8 | 2.384 | 6.0 | -0.1 | 1.9 |

[a]N, coordination number; R, distance between absorber and backscatter atoms; $\sigma^2$, Debye-Waller factor to account for both thermal and structural disorders; $\Delta E_0$, inner potential correction; R factor (%) indicates the goodness of the fit. Error bounds (accuracies) that characterize the structural parameters obtained by EXAFS spectroscopy were estimated as N ± 20%; R ± 1%; $\sigma^2$ ± 20%; $\Delta E_0$ ± 20%. $S_0^2$ was fixed to 1.000 as determined from Co foil fitting. [b]Fitting range: 3.0 $\leqslant$ k (Å$^{-1}$) $\leqslant$ 10.0 and 1.0 $\leqslant$ R (Å) $\leqslant$ 3.0. [c]Fitting range: 3.0 $\leqslant$ k (Å$^{-1}$) $\leqslant$ 10.0 and 1.0 $\leqslant$ R (Å) $\leqslant$ 3.0. [d]Fitting range: 3.0 $\leqslant$ k (Å$^{-1}$) $\leqslant$ 10.0 and 1.0 $\leqslant$ R (Å) $\leqslant$ 3.0. [e]Fitting range: 3.0 $\leqslant$ k (Å$^{-1}$) $\leqslant$ 10.0 and 1.0 $\leqslant$ R (Å) $\leqslant$ 3.0. [f]Fitting range: 3.0 $\leqslant$ k (Å$^{-1}$) $\leqslant$ 10.0 and 1.0 $\leqslant$ R (Å) $\leqslant$ 3.0.

## Supplementary Note 28

The 3×3 supercell was adopted to search the Co-adsorbed stable structure. The geometrical optimizations of Co absorbed $MoS_2$ monolayer were performed for one cobalt atom located on the surface of $MoS_2$ monolayer. The three sites of high symmetry are considered, which are top of Mo, S and hexagon center. It is found when the cobalt atom is on the top of S, it will spontaneously fall into its next top of Mo. Therefore, there exist two stable geometrical structures, as shown in Supplementary Figure 53. The cobalt adsorption energy can be calculated for evaluating the structural stability using the following equation:

$$E_{ads} = E_{MoS_2+Co} - E_{MoS_2} - E_{Co}$$

where $E_{MoS_2+Co}$ is the total energy for the $MoS_2$ monolayer absorbed with one cobalt atom. $E_{MoS2}$ is the total energy for the pristine $MoS_2$ monolayer, and $E_{Co}$ is the energy for one cobalt atom. The calculated results show that 1T phased $MoS_2$ monolayer is distorted due to the introduction of cobalt adatom in Supplementary Figure 53. The adsorption energies indicate that the cobalt-adsorbed 1T-$MoS_2$ is much more stable than cobalt-adsorbed 2H-$MoS_2$ and the cobalt atom prefers to lie on the top of Mo atom.

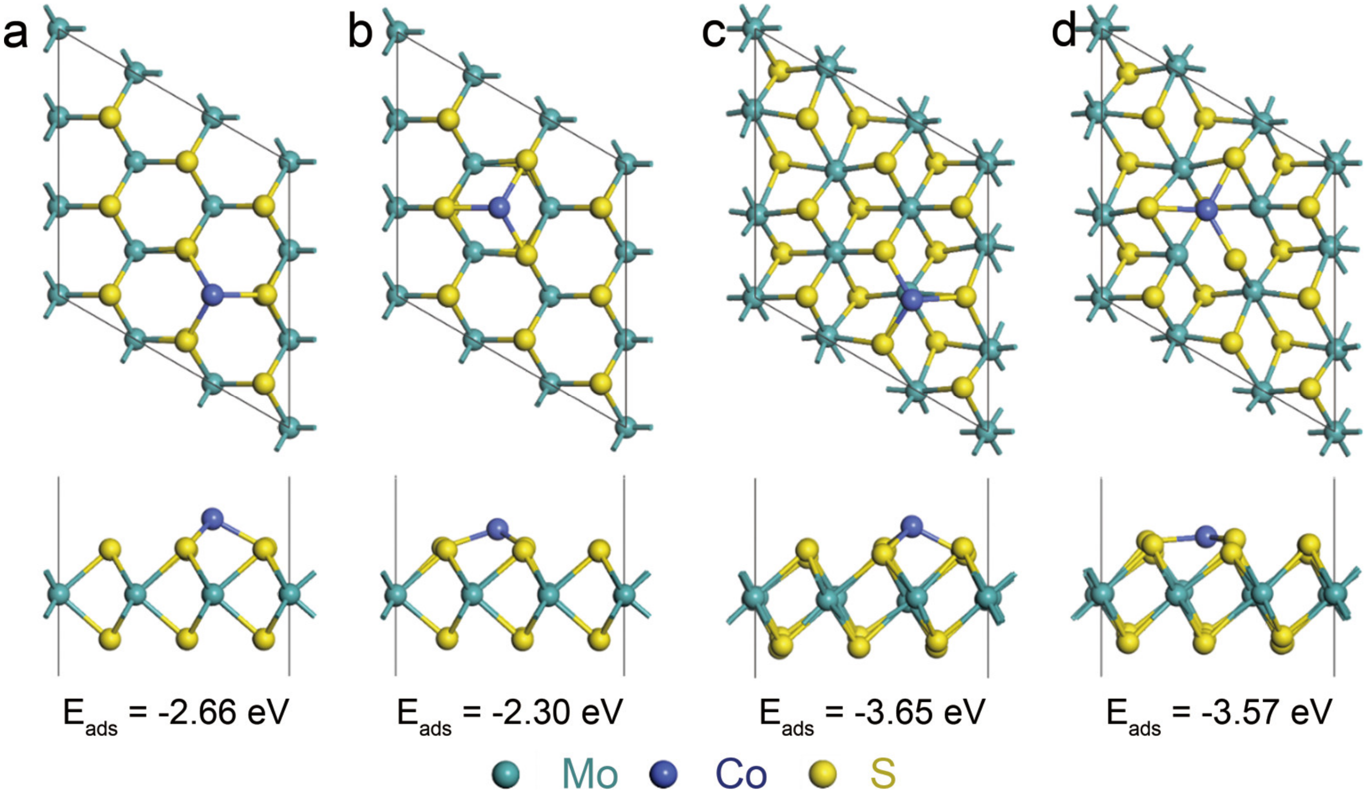


**Supplementary Figure 53.** Co adsorption models. **a-b,** 2H phased $MoS_2$ and **c-d,** D-1T $MoS_2$ structures with Co atoms on a and c Mo atop site and b and d hollow site.

## Supplementary Note 29

The stability of hydrogen can be defined by the differential hydrogen adsorption energy as described by previous studies,[38,39] which is calculated as follow:

$$\Delta E_H = E_{MoS_2+H} - E_{MoS_2} - 1/2E_{H_2}$$

where $E_{MoS2+H}$ is the total energy for the pristine or Co-adsorbed $MoS_2$ monolayer with one absorbed hydrogen atom on the surface. $E_{H_2}$ is the energy for a hydrogen molecule in the gas phase. The $\Delta E_H$ obtained from the ground state calculations does not include the contributions from the vibrational motion and the entropy. The calculation methodology for the Gibbs free energies of hydrogen adsorption is specified in literature work.[40] Therefore, the differential hydrogen binding free energies can be roughly determined as $\Delta G_H = \Delta E_H + 0.24$ eV for our models. The differential hydrogen binding free energy describes the energy needed to increase the coverage by one hydrogen atom, which is a good descriptor for hydrogen evolution reaction (HER).

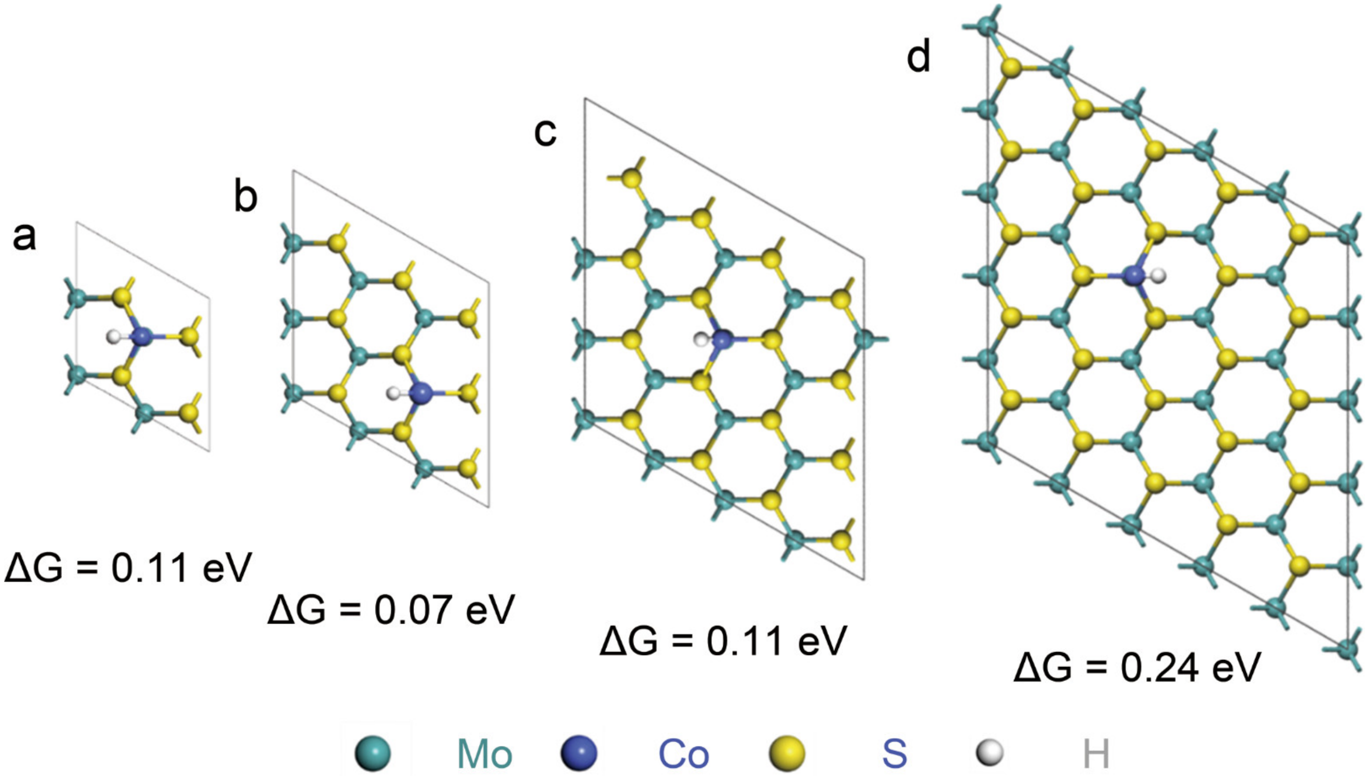


**Supplementary Figure 54.** H* adsorption models of 2H $MoS_2$ with different crystal slabs and Co adatom coverage. **a,** 2 × 2 (8.33%), **b,** 3 × 3 (3.70%), **c,** 4 × 4 (2.08%) and **d,** 5 × 5 (1.33%).

## Supplementary Note 30

Considered that Co adsorbed D-1T $MoS_2$ possesses a small value of $\Delta G_H$ of H adsorption on 3.70% Co coverage and a good stability compared with Co adsorbed 2H $MoS_2$, we have systemically calculated the free energies of H adsorption at different sites in the same D-1T $MoS_2$ model. Supplementary Figure 56 indicates that the H adsorbed pristine 1T-$MoS_2$ is the most stable (very large negative $\Delta G_H$ = -0.60 eV) whereas $\Delta G_H$ of H adsorbed on the top of Co adatom is the closest to zero ($\Delta G_H$ = 0.03 eV). More importantly, we noted that the values of $\Delta G_H$ are positive and large for other H adsorbed system, viz., H adsorbed on the top of S atom around the Co adatom, implying that the single Co adatom may be higher active site for the Co (3.70%) adsorbed on D-1T $MoS_2$.

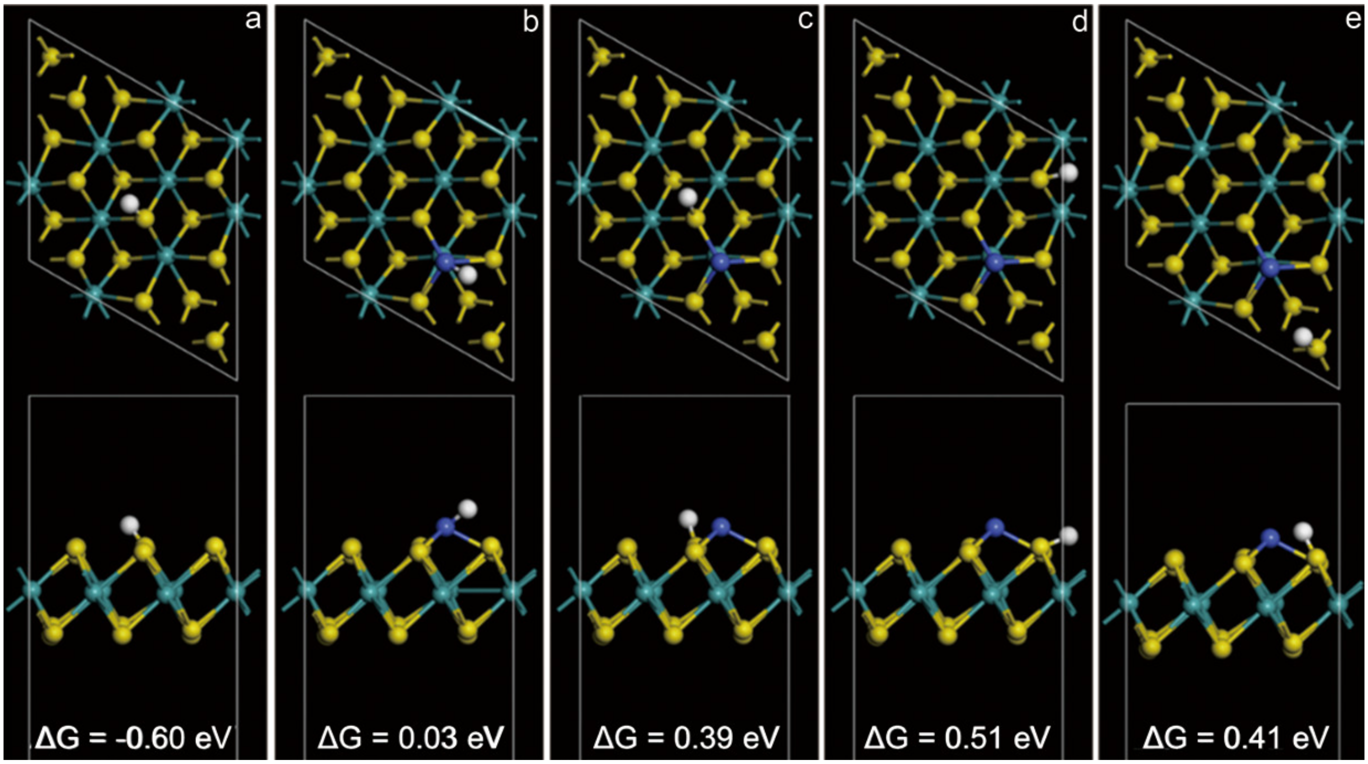


**Supplementary Figure 55.** A 3 × 3 D-1T $MoS_2$ slab with Co single atoms and H atom adsorbed on different sites. The top and side views of the geometric fragments adsorbed with hydrogen atoms and their adsorption energies $\Delta G_H^*$ are shown.

**Supplementary References:**


1 Staszak-Jirkovský, J. *et al.* Design of active and stable Co-Mo-$S_x$ chalcogels as pH-universal catalysts for the hydrogen evolution reaction. *Nat. Mater.* **15**, 197-203 (2016).

2 Fan, L. L. *et al.* Atomically isolated nickel species anchored on graphitized carbon for efficient hydrogen evolution electrocatalysis. *Nat. Commun.* **7**, 10667 (2016).

3 Liu, Q. *et al.* Gram scale aqueous synthesis of stable few layered 1T-$MoS_2$: Applications for visible light driven photocatalytic hydrogen evolution. *Small* **11**, 5556-5564 (2015).

4 Abe, E., Kawamura, Y., Hayashi, K. & Inoue, A. Long period ordered structure in a high strength nanocrystalline Mg-1 at% Zn-2 at% Y alloy studied by atomic resolution Z-contrast STEM. *Acta Mater.* **50**, 3845-3857 (2002).

5 Zhang, W. *et al.* Evolution of microstructure in TiC/NiCr cermet induced by electropulsing. *J. Mater. Res.* **18**, 1543-1550 (2003).

6 Oster, G., Wasserman, M. & Zwerling, C. Theoretical interpretation of moiré patterns. *JOSA* **54**, 169-175 (1964).

7 Li, X. G. *et al.* Single atom Pt as co-catalyst for enhanced photocatalytic $H_2$ evolution. *Adv. Mater.* **28**, 2427-2431 (2016).

8 Liu, G. L. *et al.* $MoS_2$ monolayer catalyst doped with isolated Co atoms for the hydrodeoxygenation reaction. *Nat. Chem.* **9**, 810-816 (2017).

9 Lau, T. H. M. *et al.* Transition metal atom doping of the basal plane of $MoS_2$ monolayer nanosheets for electrochemical hydrogen evolution. *Chem. Sci.* **9**, 4769-4776 (2018).

10 Kasai, H. *et al.* X-ray electron density investigation of chemical bonding in van der Waals materials. *Nat. Mater.* **17**, 249-252 (2018).

11 Fan, X. B. *et al.* Fast and efficient preparation of exfoliated 2H $MoS_2$ nanosheets by sonication assisted lithium intercalation and infrared laser induced 1T to 2H phase reversion. *Nano Lett.* **15**, 5956-5960 (2015).

12 Bai, G. X. *et al.* 2D layered materials of rare earth Er doped $MoS_2$ with NIR to NIR down and up conversion photoluminescence. *Adv. Mater.* **28**, 7472-7477 (2016).

13 Chen, Z. X. *et al.* Interface confined hydrogen evolution reaction in zero valent metal nanoparticles intercalated molybdenum disulfide. *Nat. Commun.* **8**, 14548 (2017).

14 Zhong, Y. R. *et al.* Surface chemistry in cobalt phosphide stabilized lithium sulfur batteries. *J. Am. Chem. Soc.* **140**, 1455-1459 (2018).

15 Nandi, S. *et al.* Nitrogen rich graphitic carbon stabilized cobalt nanoparticles for chemoselective hydrogenation of nitroarenes at milder conditions. *Inorg. Chem. Front.* **5**, 806-813 (2018).

16 Azcatl, A. *et al.* Covalent nitrogen doping and compressive strain in $MoS_2$ by remote $N_2$ plasma exposure. *Nano Lett.* **16**, 5437-5443 (2016).

17 Wang, H. T. *et al.* Direct and continuous strain control of catalysts with tunable battery electrode materials. *Science* **354**, 1031-1036 (2016).

18 Xue, Y. *et al.* Anchoring zero valence single atoms of nickel and iron on graphdiyne for hydrogen evolution. *Nat. Commun.* **9**, 1460 (2018).

19 Cheng, Y. F. *et al.* Rh-$MoS_2$ Nanocomposite Catalysts with Pt-Like Activity for Hydrogen Evolution Reactio. *Adv. Funct. Mater.* **27**, 1700359 (2017).

20 Qiu, H. J. *et al.* Nanoporous graphene with single atom nickel dopants: An efficient and stable catalyst for electrochemical hydrogen production. *Angew. Chem. Int. Ed.* **54**, 14031-14035 (2015).

21 Huang, Y. C. *et al.* Atomically engineering activation sites onto metallic 1T-$MoS_2$ catalysts for enhanced electrochemical hydrogen evolution. *Nat. Commun.* **10**, 982 (2019).

22 Zhang, X. *et al.* Lithiation-induced amorphization of $Pd_3P_2S_8$ for highly efficient hydrogen evolution. *Nat. Catal.* **1**, 460-486 (2018).

23 Zhou, X. L. *et al.* Design and epitaxial growth of $MoSe_2$-NiSe vertical heteronanostructures with electronic modulation for enhanced hydrogen evolution reaction. *Chem. Mater.* **28**, 1838-1846 (2016).

24 Chen, Y. L. *et al.* Highly active, nonprecious electrocatalyst comprising borophene subunits for the hydrogen evolution reaction. *J. Am. Chem. Soc.* **139**, 12370-12373 (2017).

25 Jiang, P. *et al.* Tuning the activity of carbon for electrocatalytic hydrogen evolution via an iridium-cobalt alloy core encapsulated in nitrogen-doped carbon cages. *Adv. Mater.* **30**, 1705324 (2018).

26 Li, G. X. *et al.* Cobalt-cobalt phosphide nanoparticles@nitrogen-phosphorus doped carbon/graphene derived from cobalt ions adsorbed saccharomycete yeasts as an efficient, stable, and large-current-density electrode for hydrogen evolution reactions. *Adv. Funct. Mater.* **28**, 1801332 (2018).

27 Shen, M. Q. *et al.* Approaching the volcano top: Iridium/silicon nanocomposites as efficient electrocatalysts for the hydrogen evolution reaction. *ACS Nano* **13**, 2786-2794 (2019).

28 Li, F. *et al.* Balancing hydrogen adsorption/desorption by orbital modulation for efficient hydrogen evolution catalysis. *Nat. Commun.* **10**, 4060 (2019).

29 Park, J. Y. *et al.* Investigation of the Support Effect in Atomically Dispersed Pt on $WO_{3-x}$ for Utilization of Pt in the Hydrogen Evolution Reaction. *Angew. Chem.* **131**, 1-6 (2019).

30 Chi, J. Q. *et al.* Embedding RhPx in N, P Co-doped carbon nanoshells through synergetic phosphorization and pyrolysis for efficient hydrogen evolution. *Adv. Funct. Mater.* **29**, 1901790 (2019).

31 Mishra, I. K. *et al.* Hierarchical CoP/$Ni_5P_4$/CoP microsheet arrays as a robust pH-universal electrocatalyst for efficient hydrogen generation. *Energy Environ. Sci.* **11**, 2246-2252 (2018).

32 Yan, H. *et al.* Bottom up precise synthesis of stable platinum dimers on graphene. *Nat. Commun.* **8**, 1070 (2017).

33 Qi, K. *et al.* Decoration of the inert basal plane of defect-rich $MoS_2$ with Pd atoms for achieving Pt-similar HER activity. *J. Mater. Chem. A* **4**, 4025-4031 (2016).

34 Pan, Y. *et al.* Design of single atom Co-$N_5$ catalytic site: A robust electrocatalyst for $CO_2$ reduction with nearly 100% CO selectivity and remarkable stability. *J. Am. Chem. Soc.* **140**, 4218-4221 (2018).

35 Chen, Y. X. *et al.* Fabrication, characterization, and stability of supported single atom catalysts. *Catal. Sci. Technol.* **7**, 4250-4258 (2017).

36 DeRita, L. *et al.* Catalyst architecture for stable single atom dispersion enables site specific spectroscopic and reactivity measurements of CO adsorbed to Pt atoms, oxidized Pt clusters, and metallic Pt clusters on $TiO_2$. *J. Am. Chem. Soc.* **139**, 14150-14165 (2017).

37 Zhang, Z. L. *et al.* Thermally stable single atom Pt/m-$Al_2O_3$ for selective hydrogenation and CO oxidation. *Nat. Commun.* **8**, 16100 (2017).

38 Hinnemann, B. *et al.* Biomimetic hydrogen evolution: $MoS_2$ nanoparticles as catalyst for hydrogen evolution. *J. Am. Chem. Soc.* **127**, 5308-5309 (2005).

39 Voiry, D. *et al.* Enhanced catalytic activity in strained chemically exfoliated $WS_2$ nanosheets for hydrogen evolution. *Nat. Mater.* **12**, 850-855 (2013).

40 Jaramillo, T. F. *et al.* Identification of active edge sites for electrochemical $H_2$ evolution from $MoS_2$ nanocatalysts. *Science* **317**, 100-102 (2007).
