# Supplementary material for: Single-atom cobalt array bound to distorted 1T MoS2 with ensemble effect for hydrogen evolution catalysis
Source: Nat Commun. 2019 Nov 19;10:5231. doi: 10.1038/s41467-019-12997-7 (PMC6863867; doi:10.1038/s41467-019-12997-7)
Supplement: Supplementary file 2 — Peer Review File [file 41467_2019_12997_MOESM2_ESM.pdf]

REVIEWERS' COMMENTS:

Reviewer #1 (Remarks to the Author):

I have carefully read their response and the revised manuscript. I believe the authors have addressed most of the concerns. However, I have noticed one issue. The authors provided Figure R3-3b and claimed that it is a HAADF-STEM image. Can the authors double check that? It looks like a HRTEM image not a HAADF-STEM image.

To Reviewer 1:

I have carefully read their response and the revised manuscript. I believe the authors have addressed most of the concerns. However, I have noticed one issue. The authors provided Figure R3-3b and claimed that it is a HAADF-STEM image. Can the authors double check that? It looks like a HRTEM image not a HAADF-STEM image.

Response: We acknowledge the reviewer's comment on "the author have addressed most of the concerns". For Supplementary Figure 27b (Figure R3-3b), we have double checked with it. Yes, it is an FFT refined HRTEM image, we have modified this clerical mistake in both the revised manuscript and supplementary information.
